# Global, regional, and national cataract burden attributable to household air pollution and smoking (1990–2021) and projection to 2050

**DOI:** 10.3389/fpubh.2026.1842269

**Published:** 2026-07-01

**Authors:** Qunwu Tan, Xin Gong

**Affiliations:** 1 Department of Ophthalmology, Wushan County People's Hospital of Chongqing, Wushan, China; 2 Department of Otolaryngology, Wushan County People's Hospital of Chongqing, Wushan, China

**Keywords:** ARIMA, cataract, global burden of disease, health inequality, household air pollution, smoking, YLDs

## Abstract

**Background:**

Household air pollution (HAP) and smoking, as key environmental risk factors, substantially drive the growing global cataract burden and pose serious public health threats, especially in low- and middle-income regions. This study aimed to identify the trends, spatiotemporal patterns, and key influencing factors of cataract burden attributable to HAP and smoking during 1990–2021, and further project its future trends until 2050.

**Methods:**

Based on Global Burden of Disease Study (GBD) 2021 data, we computed years lived with disability (YLDs) and age-standardized YLD rates (ASYRs) by age, gender and the Socio-demographic Index (SDI). We employed decomposition analysis to identify drivers of temporal changes, frontier analysis to assess achievable health outcomes, and inequality metrics to evaluate disparities. Future burdens were forecasted using the Autoregressive Integrated Moving Average (ARIMA) model.

**Results:**

Globally, there were 1,956,323.97 (95% UI: 612,092.84–4,033,705.23) and 225,174.47 (95% UI: 160,701.43–315,104.99) YLDs due to cataract attributable to HAP and smoking in 2021, respectively, both more than in 1990. Moreover, the ASYRs of cataract attributable to HAP and smoking were 22.84 (95% UI: 7.15–47.05) and 2.60 (95% UI: 1.85–3.63) per 100,000 population in 2021, respectively. Regionally, the highest ASYRs of cataract attributable to HAP and smoking were in Oceania (75.98) and South Asia (5.64), respectively. The cataract YLDs linked to HAP and smoking were predominantly found in middle-aged and older adult populations. Males had a higher smoking-attributable cataract burden than females, whereas females faced a substantial HAP-attributable burden. Low SDI regions exhibited a higher disease burden, with persistent cross-country disparities in health outcomes. Decomposition analysis pinpointed population growth as the core contributing factor. Frontier analysis revealed that countries such as Pakistan were farthest from the cataract burden frontier. ARIMA projections show HAP-related cataract ASYR will fall from 22.71 per 100,000 (2022) to 11.18 per 100,000 (2050, −50.77%), and smoking-attributable ASYR from 2.53 per 100,000 to 1.08 per 100,000 (−57.31%).

**Conclusion:**

Despite the observed decline in cataract burden attributable to HAP and smoking from 1990 to 2021, disparities among different sociodemographic regions and sexes were remarkable, and the potential future risks underscore the necessity for continued air quality management and tobacco control measures.

## 1 Introduction

### 1.1 Global burden and etiology of cataract

Cataract, which affects the eye’s natural crystalline lens and is a leading cause of visual impairment and blindness worldwide, constitutes a major global public health challenge (1, 2). In 2021, approximately 101 million people worldwide had blindness or visual impairment due to cataract, with an age-standardized prevalence rate (ASPR) of 1,181 per 100,000 population; notably, both the ASPR and the number of affected individuals were higher in females than in males (3). The prevalence of cataracts is positively associated with age, such that over two-thirds of the population aged 80 and older are affected by the condition (2). Currently, cataract has no effective pharmacological management, and surgery remains the sole curative method for addressing this condition (4). Most cataracts occur in low- and middle-income countries (LMICs), and cataract-associated visual impairment remains both a cause and an outcome of material poverty in resource-constrained settings (3, 5). As a result of inadequate resources and insufficient surgical workforce, treatable cataracts account for up to 50% of blindness, whereas this proportion is only 5% in high-income countries (6). Cataract may progress to cause visual impairment over time, increasing the risk of anxiety, depression, and falls in older adults and significantly impairing an individual’s quality of life and productivity (7–9).

The etiology of cataracts is multi-factorial, including aging (10), smoking (11), air pollution (12), comorbid ocular diseases (such as myopia and uveitis) (13), systemic diseases (such as diabetes, hypertension, and obesity) (14), among other factors. Aging and oxidative stress are the predominant drivers of cataract genesis. Since lens transparency depends on stable metabolic nourishment and antioxidant protection supplied by the aqueous humour, aging-related impairment of ocular transport and redox function reduces soluble crystallin synthesis and induces insoluble protein aggregation, while persistent oxidative damage and peptide degradation further disrupt protein structure and function (15). The progressive accumulation of chromophores ultimately leads to lens brunescence and subsequent cataract development (16). Unlike irreversible aging-induced pathological changes, most behavioral and environmental risk factors are modifiable and preventable. Among these, smoking and air pollution represent key preventable environmental determinants, providing crucial targets for global cataract burden reduction.

### 1.2 Household air pollution and cataract risk

Growing epidemiological evidence indicates that air pollution exerts substantial adverse effects on human health, responsible for 6.67 million global deaths in 2019 per the State of Global Air 2020 (17). Unlike ambient air pollution that is largely affected by external environmental factors, household air pollution (HAP) is more susceptible to individual daily behaviors and living habits, making it more amenable to targeted prevention and intervention at the personal level. Globally, approximately 3 billion individuals are exposed to hazardous concentrations of HAP generated by the use of solid cooking fuels (18). These solid fuels are often the only viable cooking option in LMICs, where cleaner energy alternatives such as liquefied petroleum gas and electricity remain scarce or financially unaffordable (19). However, the combustion of solid fuels generates numerous toxic pollutants, including high levels of fine particulate matter (PM_2.5_), a well-documented hazard to human health (20). HAP is responsible for 3.11 million deaths and 111 million disability-adjusted life years (DALYs) in 2021 (18). Long-term exposure to HAP is associated with a spectrum of ocular complications, from ocular surface conditions such as conjunctivitis and dry eye disease to cataracts, which can eventually culminate in vision loss and blindness (12, 21, 22). It can induce reactive oxygen species and nitrogen-mediated oxidative stress, which disrupts membrane integrity, impairs protein secretion, and promotes the accumulation of damaged lens proteins upon prolonged exposure, thereby precipitating cataract onset (23, 24). According to WHO estimates, 63.1 million (95% uncertainty intervals [UI]: 37.8–120.4) individuals worldwide relied mainly on kerosene for cooking in 2021 (18). As reported in an Indian study, women using kerosene for domestic energy exhibited a significantly elevated risk of cataracts (OR = 1.76, 95% confidence intervals [CI]: 1.04–2.97) (25).

### 1.3 Smoking and cataract risk

Furthermore, smoking represents another significant and modifiable risk factor (26). In 2019, smoking contributed to 7.69 million (95% UI: 7.16–8.20) deaths and 200 million (95% UI: 185–214) DALYs globally (27). The WHO’s 2019 seventh report on the global tobacco epidemic noted that approximately 80% of smokers reside in LMICs, highlighting these regions as the core priority for current tobacco cessation interventions (28). Although smoking prevalence has declined in recent years, the global tobacco epidemic still imposes substantial health and economic burdens worldwide, making tobacco control an urgent public health priority (29). Cigarette smoke is a complex mixture of over 7,000 chemicals, including particulates (e.g., nicotine), gases (e.g., carbon monoxide), and volatile compounds (e.g., formaldehyde) (30). These harmful substances and free radicals can directly or indirectly damage ocular tissues, contributing to various blinding eye diseases, including cataracts (31, 32). Extensive research demonstrates that smokers have a 2–3fold higher risk of developing cataracts than non-smokers (33, 34). Smoking impairs lenticular function and induces lens opacification through multiple mechanisms, including direct oxidative damage via free radicals, indirect oxidative stress from depleted antioxidants, and protein modification by isocyanates and aldehydes (35–38). Tobacco-related heavy metals (e.g., cadmium, lead, copper) accumulate in the lens and exert direct toxicity; notably, cadmium levels in the blood and lens are significantly higher in smokers, with a 3–4fold increase in cataractous lenses of smokers relative to non-smokers and a strong correlation between blood and lens cadmium concentrations in heavy smokers (39–41).

### 1.4 Study objective and significance

Existing studies based on GBD 2021 data have explored cataract burden attributable to smoking or HAP separately. However, these studies present prominent limitations. Some solely focus on smoking-related cataract burden, whereas others only assess HAP-associated visual impairment and blindness (32, 42). Few studies have integrated health inequality analysis, frontier analysis, trend decomposition, and future forecasting, so the temporal drivers, socioeconomic disparities, and long-term trajectories of cataract burden linked to these two risk factors remain insufficiently understood. To fill these gaps, this study systematically evaluated the global cataract burden attributable to both HAP and smoking from 1990 to 2021. We adopted decomposition, inequality, frontier, and ARIMA forecasting analyses to clarify the temporal dynamics, socioeconomic disparities, and future trends of this burden. ARIMA model specification was determined via ACF and PACF pattern identification, complemented by comprehensive residual diagnostics comprising Ljung–Box white-noise testing and normality assessment to ensure reliable forecast outputs. These two modifiable environmental risks frequently coexist in LMICs. Populations exposed to HAP from solid cooking fuels often have higher smoking prevalence, leading to dual exposure that may generate synergistic adverse effects on cataract risk. Understanding these long-term trends is critical for evaluating household air quality and tobacco control interventions and informing evidence-based eye health policies. This study further characterized the global, regional, and national distribution patterns and driving factors of HAP- and smoking-related cataract burden and projected their trends to 2050, aiming to support targeted prevention among high-risk regions and vulnerable populations and provide practical evidence for public health decision-making.

## 2 Methods

### 2.1 Overview

This study performed a systematic secondary analysis based on data retrieved from the GBD 2021 database—a comprehensive resource curated by the Institute for Health Metrics and Evaluation (IHME) at the University of Washington. This database systematically compiles the nonfatal burden associated with 371 diseases and injuries, alongside 88 risk factors, across 204 countries and territories worldwide (43). Data sources encompassed national censuses, surveys, administrative registries, and hospital records—all processed via the Bayesian meta-regression tool DisMod-MR 2.1 to generate consistent estimates. This study retrieved the number and rate of years lived with disability (YLDs) linked to the cataract burden attributable to HAP and smoking across different age groups, sexes, Sociodemographic Index (SDI) levels, regions, and nations from 1990 to 2021 via the accessible online Global Health Data Exchange (GHDx) platform, which is available at https://gbd2021.healthdata.org/gbd-results/. A sequential multi-dimensional analytical framework was adopted in this study, consisting of temporal trend analysis, driving factor decomposition analysis, health efficiency frontier analysis, socioeconomic inequality assessment, and ARIMA-based future projection. This integrated workflow comprehensively quantified the spatiotemporal patterns, multidimensional disparities, dynamic driving factors, and future evolutionary trajectories of the global cataract burden attributable to HAP and smoking.

### 2.2 Definition and data sources

Cataract is defined as the opacification of the lens caused by denaturation and aggregation of lens proteins, thereby impairing vision (1–3). The definition of cataract was based on the International Classification of Diseases (ICD) coding system, primarily using ICD 10 codes H25–H26.9 and H28–H28.8 to ensure consistency for historical data analysis. HAP, classified as a Level 4 risk factor in the GBD study, is predominantly produced by the combustion of solid cooking fuels (e.g., coal, charcoal, wood, and agricultural residues), leading to heightened air pollution exposure in the affected populations (18). Additionally, the 2021 Global Health Guidelines characterize smoking as the current use of any tobacco products or former smokers who have not smoked for at least 6 months (44). In the GBD 2021 framework, cataract burden attributable to HAP and smoking was quantified through the standardized Comparative Risk Assessment (CRA) approach rather than direct observation of individual exposure-outcome pairs. This modeling method calculates the population attributable fraction (PAF) by comparing current population exposure levels with the theoretical minimum risk exposure level (TMREL) (43).

In our data search, we used ‘cataract’ as the cause, while ‘household air pollution from solid fuels’ and ‘smoking’ served as the two risk factors under investigation. We measured ‘YLDs’ and age-standardized YLD rates (ASYRs) from 1990 to 2021, utilizing metrics such as number and rate. YLDs are defined as years lived with any short-term or long-term health loss weighted for severity by the disability weights (45). The number and rate of YLDs, along with their 95% uncertainty intervals (UI), of cataract cases linked to HAP and smoking were gathered, broken down by year, sex, region, and country. Notably, the negative YLD values in our results represent the lower bound of 95% UIs from official GBD estimates rather than real negative disease burden. Extremely low actual burden may cause the intervals to cross zero and produce negative lower limits. We strictly used original GBD data without recalculation or revision, and these values do not affect our overall trends and core conclusions. The 204 countries and territories are further divided into 21 GBD regions according to their geographical locations. For cataract attributable to HAP and smoking, individuals aged 20 to 95+ years and 30 to 95+ years were, respectively, grouped into 16 and 14 categories at 5-year intervals. The SDI represents a metric of a country’s socioeconomic status, with higher values reflecting more advanced socioeconomic development. Constructed from national indicators—including per capita income, mean educational attainment, and total fertility rate—this index varies from 0 to 1. The GBD 2021 study categorizes 204 countries and territories into five development strata based on SDI: high (>0.8103), high-middle (0.7120–0.8103), middle (0.6188–0.7120), low-middle (0.4658–0.6188), and low (≤0.4658) (46).

### 2.3 Estimation of disease burden

To gain a more intuitive understanding of the temporal trends in cataract burden linked to HAP and smoking, estimated annual percentage changes (EAPCs) were calculated. A linear regression line was fitted to the natural logarithm of ASYR values, based on the equation *y* = *α* + *β*x + e, where y represents ln (ASYR) and x represents the calendar year. Subsequently, EAPCs and its corresponding 95% CI were derived using the formula EAPC = 100 × (exp[β] − 1), wherein β stands for the slope of the log-linear regression model. An upward trend in ASYR was indicated when the 95% CI of EAPC exceeded 0, whereas a downward trend was observed if the CI was below 0. The indicator remained stable if the 95% CI encompassed 0. Spearman correlation was employed to assess the association between the cataract burden and the SDI.

### 2.4 Cross-country inequality analysis

To evaluate absolute and relative disparities in the cataract burden attributable to HAP and smoking, cross-country health inequality analysis was conducted via the calculation of the slope index of inequality (SII) and the concentration index (CI) (47). The SII was derived by regressing country-level ASYRs for cataract related to HAP and smoking (across all age groups) against the sociodemographic development-associated relative position scale, which is determined by the midpoint of the cumulative class range of populations ranked by SDI. To alleviate the effects of outliers and homoscedasticity violations on the results, a robust linear model (RLM) was utilized for the estimation of the SII. The CI was computed by fitting a Lorenz concentration curve to the observed cumulative relative distribution of SDI-ranked populations and ASYRs of disease, along with numerical integration of the area under the curve. Negative SII and CI values imply a higher SDI corresponds to a lower ASYR (vice versa), with larger absolute values of these indices indicating more significant health inequality.

### 2.5 Decomposition analysis

To identify the key drivers of temporal changes in global HAP- and smoking-attributable cataract burden between 1990 and 2021, we employed the Das Gupta decomposition method, which decomposes overall YLDs changes into contributions from aging, population growth, and epidemiological shifts (48). This analytical framework enabled us to quantify the relative magnitude of each factor’s impact, clarify how demographic dynamics and epidemiological trends collectively shaped the burden trajectory over the three-decade period, and ultimately provide evidence to inform targeted public health interventions for mitigating cataract-related health losses associated with HAP and smoking.

### 2.6 Frontier analysis

To investigate the link between sociodemographic development levels and the cataract burden caused by HAP and smoking, frontier analysis was used to build a frontier model based on ASYR, with reference to the SDI (49). Frontier analysis determines the theoretical minimum ASYR value for each country/territory (based on current development level and serving as an optimal performance benchmark), quantifies the current-potential burden gap, and identifies improvement areas. Locally weighted regression (LOESS) was combined with local polynomial regression to generate smooth frontier lines, with three distinct smoothing spans (0.3, 0.4, 0.5) utilized for estimating the nonlinear association between SDI and cataract ASYR. The absolute vertical distance between each country/territory’s 2021 ASYR and the frontier line represented the efficiency difference, with a larger distance indicating lower public health efficiency and greater potential for disease prevention. Countries distant from the frontier, such as Pakistan, had higher HAP- and smoking-attributable cataract burden than the global optimal level at similar SDI levels.

### 2.7 ARIMA model projection

To estimate the future trajectory of the cataract burden attributable to HAP and smoking, we performed projections from 2022 to 2050 using the autoregressive integrated moving average (ARIMA) model. Selected for its suitability for time-series data, this model—typically denoted as ARIMA (*p*, *d*, *q*), where *p* = autoregressive order, *d* = differencing for stationarity, and *q* = moving average order—integrates three key components (autoregression, differencing, and moving average) to effectively capture temporal trends (50, 51). We applied the auto.arima() function in the R forecast package to select the optimal ARIMA (p, d, q) model based on the minimum Akaike information criterion (AIC). A stepwise algorithm was adopted (stepwise = TRUE), and approximation was disabled (approximation = FALSE) to ensure estimation accuracy. The autocorrelation function (ACF) and partial autocorrelation function (PACF) of the original 1990–2021 time series were calculated. The ACF reflects the temporal dependence structure of the data, while the PACF identifies the direct effect of each lag after controlling for intermediate lagged variables. An ACF cutoff at lag q implies an MA(q) (Moving Average) specification, whereas a PACF truncation at lag p points to an AR(p) (Autoregressive) structure. Residual ACF and PACF were also examined to confirm the absence of significant autocorrelation after model fitting. The Ljung–Box test was used to verify whether model residuals conformed to a white noise distribution. The null hypothesis (H₀) assumed that the residual series was independently and identically distributed without autocorrelation, under which the test statistic Q follows a chi-square distribution. H₀ could not be rejected at *p* > 0.05, indicating that the model fully extracted the temporal structural information of the original series with no remaining systematic residual patterns. The test lag order was adaptively determined as lag = min(10, floor(n/5)) ≈ 6 (*n* = 32) to balance test power and degrees of freedom. The model was fitted on the full 1990–2021 training dataset, and the forecast() function was used to generate predictions up to 2050. Notably, prediction intervals widen significantly in the long term due to the cumulative uncertainty of long-term projections.

### 2.8 Statistical analysis

Spearman correlation analysis was utilized to examine the relationship between the cataract burden and the SDI. Our study was performed in accordance with the Guidelines for Accurate and Transparent Health Estimates Reporting (GATHER) (52). Statistical significance was set at *p* < 0.05. Database construction, collation and analysis were performed using the R software (version 4.3.3). R software (version 4.6.0) was adopted for the in-depth ARIMA analyses conducted during revision.

## 3 Results

### 3.1 Global level

In 2021, global cataract YLDs attributable to HAP and smoking were 1,956,323.97 (95% UI: 612,092.84–4,033,705.23) and 225,174.47 (95% UI: 160,701.43–315,104.99), respectively, representing increases of 38.35 and 38.93%, respectively, from 1990 (Tables 1 and 2).

**Table 1:** The number of YLDs and ASYRs of cataract attributable to HAP in 1990 and 2021, stratified by sex and SDI, with corresponding EAPCs (1990–2021) at global and regional levels.

Location	1990		2021		1990–2021
	Number of YLDs, (95% UI)	ASYR per 100,000, (95% UI)	Number of YLDs, (95% UI)	ASYR per 100,000, (95% UI)	EAPC, %, (95% CI)
Global	1,414,029.27 (564,248.53–2,610,528.48)	37.09 (14.86–68.78)	1,956,323.97 (612,092.84–4,033,705.23)	22.84 (7.15–47.05)	−1.51 (−1.71 to −1.30)
Sex					
Male	620,868.66 (248,033.77–1,143,757.97)	36.90 (14.88–68.52)	812,838.91 (256,095.08–1,678,678.64)	20.66 (6.52–42.57)	−1.84(−2.04 to −1.64)
Female	793,160.60 (316,214.77–1,473,985.62)	37.66 (15.04–70.04)	1,143,485.06 (355,997.77–2,351,333.89)	24.75 (7.71–50.92)	−1.28(−1.50 to −1.07)
SDI regions					
High SDI	16,617.13 (3,620.72–39,349.32)	1.50 (0.32–3.55)	10,506.53 (1,508.40–32,030.26)	0.48 (0.07–1.46)	−3.83 (−4.02 to −3.64)
High-middle SDI	130,009.16 (43,845.35–264,760.84)	13.98 (4.71–28.74)	151,131.55 (30,128.62–363,678.82)	7.66 (1.53–18.48)	−1.83 (−2.24 to −1.43)
Middle SDI	434,879.10 (155,246.05–835,151.40)	49.06 (17.85–94.92)	554,077.66 (123,509.86–1,255,528.24)	21.97 (4.94–49.73)	−2.49 (−2.80 to −2.19)
Low-middle SDI	635,812.06 (273,581.00–1,145,701.54)	117.98 (51.29–213.22)	902,564.95 (313,107.7–1,739,868.98)	68.31 (23.83–132.18)	−1.73 (−1.87 to −1.6)
Low SDI	195,953.69 (87,185.56–351,151.97)	99.35 (45.19–177.44)	337,167.31 (143,480.36–614,216.94)	74.26 (31.93–135.59)	−0.91 (−1.02 to −0.80)
GBD regions					
East Asia	229,951.41 (95,347.61–424,443.51)	32.48 (13.79–59.87)	342,038.31 (79,162.99–760,631.96)	16.52 (3.85–36.93)	−1.96 (−2.44 to −1.47)
Oceania	2,445.69 (1,036.83–4,478.85)	96.41 (41.65–177.28)	5,021.32 (2,037.81–9,344.86)	75.98 (31.18–142.86)	−0.77 (−0.88 to −0.65)
Southeast Asia	197,981.93 (77,211.92–370,996.34)	88.56 (35.11–165.11)	238,192.66 (66,853.44–516,767.94)	40.49 (11.50–87.18)	−2.65 (−2.85 to −2.45)
Central Sub-Saharan Africa	3,184.85 (1,397.52–5,921.63)	17.65 (8.00–32.33)	5,195.78 (1,868.21–10,161.51)	11.68 (4.59–22.71)	−1.26 (−1.37 to −1.15)
Eastern Sub-Saharan Africa	55,014.29 (24,333–99,780.17)	78.50 (35.54–140.47)	101,294.83 (44,833.94–181,474.82)	63.92 (28.95–115.91)	−0.57 (−0.61 to −0.52)
Southern Sub-Saharan Africa	11,697.89 (3,665.18–23,922.25)	45.72 (14.49–93.95)	9,361.08 (2,137.65–20,827.09)	17.78 (4.07–40.02)	−3.44 (−3.58 to −3.30)
Western Sub-Saharan Africa	71,078.68 (31,890.64–128,344.28)	89.39 (40.59–163.33)	128,053.27 (49,236.59–241,756.59)	70.91 (27.55–134.48)	−0.85 (−0.96 to −0.73)
South Asia	707,906.62 (296,519.93–1,279,271.98)	142.40 (60.57–258.32)	1,030,038.29 (347,130.34–2,022,065.08)	75.62 (25.61–149.39)	−1.97 (−2.14 to −1.81)
Andean Latin America	11,176.04 (3,527.68–22,267.13)	59.68 (18.98–119.99)	10,265.88 (1,830–26,298.74)	17.93 (3.21–45.78)	−3.95 (−4.26 to −3.65)
Caribbean	3,038.23 (988.74–6,667.41)	12.16 (3.94–26.75)	2,773.76 (916.13–6,166.78)	5.19 (1.72–11.49)	−2.72 (−2.86 to −2.58)
Central Latin America	18,346.31 (4,681.70–40,225.93)	24.77 (6.40–53.67)	22,650.62 (4,292.99–59,220.13)	9.47 (1.80–24.68)	−3.11 (−3.21 to −3.01)
Tropical Latin America	23,128.91 (5,641.94–49,738.77)	29.30 (7.28–63.17)	16,824.78 (2,805.54–46,687.83)	6.77 (1.13–18.80)	−4.46 (−4.57 to −4.34)
North Africa and Middle East	40,214.68 (9,817.72–93,597.90)	27.83 (6.83–65.09)	20,930.51 (48,46.23–48,503.29)	5.09 (1.18–11.93)	−5.45 (−5.63 to −5.27)
Central Asia	10,017.08 (2,523.45–21,975.83)	23.24 (5.89–50.64)	7,001.66 (1,195.30–18,648.69)	9.70 (1.68–25.80)	−3.18 (−3.46 to −2.90)
Central Europe	9,070.88 (2,235.83–19,914.19)	6.51 (1.62–14.22)	7,808.98 (1,386.38–19,884.9)	3.42 (0.60–8.70)	−2.17 (−2.23 to −2.11)
Eastern Europe	10,299.87 (1,791.60–28,103.43)	3.96 (0.69–10.77)	4,553 (571.24–14,441.04)	1.29 (0.16–4.10)	−4.12 (−4.39 to −3.85)
Australasia	245.40 (26.61–783.76)	1.08 (0.12–3.49)	89.13 (9.98–310.37)	0.16 (0.02–0.55)	−6.45 (−6.73 to −6.17)
High-income Asia Pacific	1,094.72 (127.82–3,745.98)	0.57 (0.07–1.96)	452.23 (41.07–1,619.46)	0.09 (0.01–0.32)	−6.14 (−6.55 to −5.73)
High-income North America	393.63 (41.18–1,362.30)	0.11 (0.01–0.38)	415.77 (42.06–1,518.09)	0.06 (0.01–0.23)	−2.27 (−2.46 to −2.07)
Southern Latin America	2,938.26 (485.32–7,717.88)	6.73 (1.12–17.70)	1,694.78 (210.66–5,782.00)	1.90 (0.24–6.51)	−4.35 (−4.44 to −4.26)
Western Europe	4,803.90 (588.55–15,947.97)	0.83 (0.10–2.77)	1,667.33 (189.29–6,357.26)	0.17 (0.02–0.62)	−5.28 (−5.47 to −5.10)

YLDs, years lived with disability; ASYR, age standardized YLD rate; HAP, household air pollution; SDI, socio-demographic index; EAPC, estimated annual percentage change; UI, uncertainty intervals; CI, confidence intervals.

**Table 2:** The number of YLDs and ASYRs of cataract attributable to smoking in 1990 and 2021, stratified by sex and SDI, with corresponding EAPCs (1990–2021) at global and regional levels.

Location	1990		2021		1990–2021
	Number of YLDs, (95% UI)	ASYR per 100,000, (95% UI)	Number of YLDs, (95% UI)	ASYR per 100,000, (95% UI)	EAPC, %, (95% CI)
Global	162,083.20 (115,037.07–221,809.41)	4.11 (2.94–5.63)	225,174.47 (160,701.43–315,104.99)	2.60 (1.85–3.63)	−1.41 (−1.48 to −1.33)
Sex					
Male	131,529.18 (93,031.40–180,120.51)	7.38 (5.30–10.1)	184,884.12 (130,572.35–258,505.19)	4.59 (3.24–6.39)	−1.46 (−1.52 to −1.40)
Female	30,554.02 (21,970.69–41,654.07)	1.45 (1.05–1.97)	40,290.35 (28,390.72–56,789.54)	0.87 (0.61–1.23)	−1.60 (−1.77 to −1.44)
SDI regions					
High SDI	15,326.94 (10,710.47–21,854.00)	1.42 (0.99–2.03)	17,228.00 (11,581.39–25,167.73)	0.89 (0.60–1.31)	−1.60 (−1.70 to −1.50)
High-middle SDI	27,352.02 (19,450.60–37,917.28)	2.77 (1.96–3.83)	44,388.01 (31,100.05–63,253.29)	2.25 (1.58–3.19)	−0.41 (−0.55 to −0.27)
Middle SDI	51,857.24 (36,896.84–71,636.82)	5.22 (3.72–7.17)	78,233.64 (55,295.81–110,583.46)	2.93 (2.08–4.13)	−1.77 (−1.88 to −1.67)
Low-middle SDI	57,281.04 (40,924.22–78,506.94)	9.97 (7.16–13.55)	72,051.42 (51,674.44–99,414.75)	5.22 (3.72–7.18)	−2.08 (−2.14 to −2.02)
Low SDI	10,156.74 (7,092.99–13,910.14)	4.87 (3.44–6.55)	13,143.80 (9,350.05–18,244.51)	2.77 (1.97–3.83)	−1.90 (−2.08 to −1.73)
GBD regions					
East Asia	31,913.31 (22,456.94–44,321.03)	3.82 (2.70–5.26)	60,300.70 (41,336.10–85,029.82)	2.74 (1.88–3.87)	−0.60 (−0.83 to −0.37)
Oceania	187.21 (124.55–264.42)	5.83 (3.86–8.17)	372.36 (248.69–553.98)	4.46 (3.02–6.63)	−0.91 (−1.00 to −0.82)
Southeast Asia	19,522.34 (13,921.54–26,799.33)	7.98 (5.68–10.92)	27,757.70 (19,571.69–38,612.75)	4.28 (3.03–5.92)	−2.23 (−2.34 to −2.12)
Central Sub-Saharan Africa	118.55 (78.34–168.27)	0.52 (0.35–0.72)	202.73 (137.66–287.51)	0.34 (0.23–0.48)	−1.16 (−1.30 to −1.01)
Eastern Sub-Saharan Africa	1,792.22 (1,246.15–2,440.10)	2.40 (1.68–3.29)	2,402.77 (1,676.63–3,360.97)	1.36 (0.95–1.90)	−1.81 (−1.90 to −1.73)
Southern Sub-Saharan Africa	1,386.39 (964.78–1,975.70)	5.10(3.53–7.18)	1,001.44 (678.48–1,388.86)	1.67 (1.14–2.31)	−3.89 (−4.09 to −3.68)
Western Sub-Saharan Africa	1,358.48 (929.88–1,908.26)	1.57 (1.08–2.20)	2,570.15 (1,727.63–3,656.11)	1.26 (0.85–1.82)	−0.87 (−1.11 to −0.63)
South Asia	62,500.88 (44,759.57–85,730.68)	11.76 (8.40–15.95)	79,609.34 (56,688.97–110,778.81)	5.64 (4.01–7.85)	−2.38 (−2.47 to −2.30)
Andean Latin America	599.32 (395.15–879.14)	3.05 (2.01–4.50)	1,013.07 (638.08–1,557.58)	1.73 (1.09–2.69)	−2.28 (−2.44 to −2.12)
Caribbean	681.49 (465.74–999.22)	2.64 (1.78–3.88)	738.34 (485.43–1,102.45)	1.37 (0.90–2.04)	−2.23 (−2.32 to −2.14)
Central Latin America	2,546.89 (1,773.91–3,552.03)	3.19 (2.24–4.47)	2,783.28 (1,889.42–3,992.20)	1.12 (0.77–1.62)	−3.62 (−3.72 to −3.52)
Tropical Latin America	5,457.00 (3,690.82–7,701.24)	6.12 (4.13–8.67)	6,408.18 (4,125.17–9,643.30)	2.50 (1.62–3.77)	−2.67 (−2.98 to −2.36)
North Africa and Middle East	9,737.09 (6,766.46–13,559.27)	6.06 (4.19–8.53)	15,315.35 (10,758.89–21,665.26)	3.43 (2.40–4.82)	−1.94 (−2.00 to −1.89)
Central Asia	1,151.05 (786.28–1,636.16)	2.41 (1.66–3.43)	1,771.71 (1,207.74–2,507.19)	2.13 (1.45–3.01)	−0.20 (−0.37 to −0.03)
Central Europe	1,937.79 (1,331.14–2,766.27)	1.31 (0.90–1.86)	1,701.34 (1,145.29–2,452.11)	0.83 (0.56–1.20)	−1.58 (−1.63 to −1.54)
Eastern Europe	2,996.35 (2,126.71–4,271.98)	1.09 (0.77–1.54)	3,391.83 (2,355.99–4,813.76)	1.01 (0.71–1.44)	−0.20 (−0.48 to 0.09)
Australasia	238.64 (162.54–337.91)	1.04 (0.70–1.48)	305.65 (200.50–459.43)	0.62 (0.41–0.94)	−1.55 (−1.60 to −1.50)
High-income Asia Pacific	2,570.18 (1,803.90–3,628.93)	1.27 (0.90–1.80)	2,732.36 (1,853.59–3,974.78)	0.67 (0.46–0.96)	−2.20 (−2.27 to −2.14)
High-income North America	4,656.09 (3,170.72–6,753.25)	1.36 (0.93–1.97)	4,986.82 (3,299.80–7,621.00)	0.80 (0.53–1.21)	−1.87 (−2.04 to −1.69)
Southern Latin America	782.82 (527.43–1,145.98)	1.68 (1.13–2.46)	870.26 (562.73–1,295.05)	1.04 (0.67–1.53)	−1.54 (−1.62 to −1.47)
Western Europe	9,949.12 (6,813.75–14,294.53)	1.84 (1.26–2.61)	8,939.10 (5,888.84–12,969.15)	1.08 (0.72–1.61)	−1.73 (−1.79 to −1.67)

YLDs, years lived with disability; ASYR, age standardized YLD rate; SDI, socio-demographic index; EAPC, estimated annual percentage change; UI, uncertainty intervals; CI, confidence intervals.

**Figure 1:**
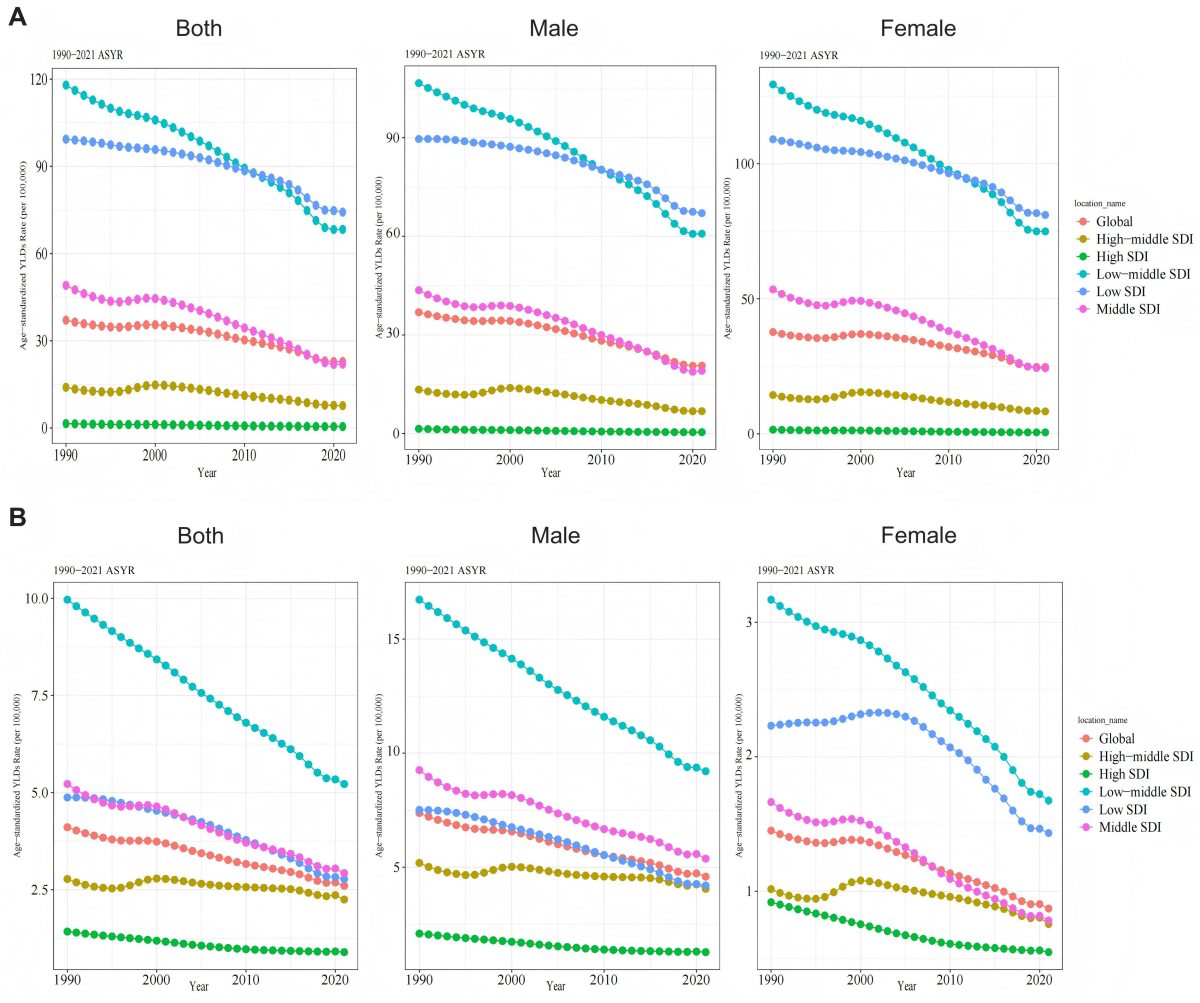
Trends in ASYR for cataract attributable to HAP **(A)** and smoking **(B)**, by sex and SDI stratification, 1990–2021. ASYR: age-standardised YLD rate. SDI, Socio-demographic Index; HAP, household air pollution.

The ASYR of cataract attributable to HAP decreased from 37.09 (95% UI: 14.86–68.78) per 100,000 population in 1990 to 22.84 (95% UI: 7.15–47.05) per 100,000 in 2021 globally, with an EAPC of −1.51 (95% CI: −1.71 to −1.30) (Table 1; Figure 1A). In 2021, the estimated global YLDs of cataract attributable to HAP for males and females were 812,838.91 (95% UI: 256,095.08–1,678,678.64) and 1,143,485.06 (95% UI: 355,997.77–2,351,333.89), respectively. The ASYR for females was greater than that for males (females: 24.75 per 100,000; 95% UI: 7.71–50.92; males: 20.66 per 100,000; 95% UI: 6.52–42.57). Notably, from 1990 to 2021, the EAPC of the ASYR in males was −1.84 (95% CI: −2.04 to −1.64), which represented a greater magnitude of decrease than that in females (−1.28, 95% CI: −1.50 to −1.07) (Table 1; Figure 1A).

The ASYR of cataract attributable to smoking decreased from 4.11 (95% UI: 2.94–5.63) per 100,000 population in 1990 to 2.60 (95% UI: 1.85–3.63) per 100,000 in 2021 globally, with an EAPC of −1.41 (95% CI: −1.48 to −1.33) (Table 2; Figure 1B). In 2021, the estimated global YLDs of cataract attributable to smoking were substantially higher in males than in females, at 184,884.12 (95% UI: 130,572.35–258,505.19) and 40,290.35 (95% UI: 28,390.72–56,789.54), respectively. The ASYR for males was greater than that for females (males: 4.59 per 100,000; 95% UI: 3.24–6.39; females: 0.87 per 100,000; 95% UI: 0.61–1.23). From 1990 to 2021, the EAPC of the ASYR in females was −1.60 (95% CI: −1.77 to −1.44), which represented a greater magnitude of decrease than that in males (−1.46, 95% CI: −1.52 to −1.40) (Table 2; Figure 1B).

### 3.2 Regional level

At the SDI regional level, the global cataract burden attributable to HAP and smoking exhibited significant regional variations. For HAP, the low-middle SDI regions had the highest number of YLDs in both 1990 (635,812.06, 95% UI: 273,581.00–1,145,701.54) and 2021 (902,564.95, 95% UI: 313,107.70–1,739,868.98) (Table 1). Notably, YLDs decreased by 36.77% in high SDI regions (from 16,617.13 [95% UI: 3,620.72–39,349.32] in 1990 to 10,506.53 [95% UI: 1,508.40–32,030.26] in 2021) but increased in all other SDI regions—particularly in low SDI regions, where they rose by 72.06% (from 195,953.69 [95% UI: 87,185.56–351,151.97] in 1990 to 337,167.31 [95% UI: 143,480.36–614,216.94] in 2021). In 2021, ASYR showed substantial disparities across SDI regions. Low SDI regions had the highest rate (74.26 per 100,000 population; 95% UI: 31.93–135.59), whereas high SDI regions had the lowest (0.48 per 100,000 population; 95% UI: 0.07–1.46) (Table 1; Figure 1A). In terms of trends, the ASYR declined to varying degrees across all SDI regions, particularly in low-middle SDI regions; it fell by 42.10% from 117.98 per 100,000 (95% UI: 51.29–213.22) in 1990 to 68.31 per 100,000 (95% UI: 23.83–132.18) in 2021, with an EAPC of −1.73 (95% CI: −1.87 to −1.60). A similar downward trend was observed in both males and females (Figure 1A).

For smoking, the highest number of cataract YLDs occurred in low-middle SDI regions in 1990 (57,281.04, 95% UI: 40,924.22–78,506.94), shifting to middle SDI regions in 2021 (78,233.64, 95% UI: 55,295.81–110,583.46) (Table 2). Notably, YLDs increased in all SDI regions, particularly in middle SDI regions, where they rose by 50.86% (from 51,857.24 [95% UI: 36,896.84–71,636.82] in 1990 to 78,233.64 [95% UI: 55,295.81–110,583.46] in 2021). In 2021, low-middle SDI regions had the highest rate (5.22 per 100,000 population; 95% UI: 3.72–7.18), whereas high SDI regions had the lowest (0.89 per 100,000 population; 95% UI: 0.60–1.31) (Table 2; Figure 1B). Over time, the ASYR declined to varying degrees across all SDI regions, especially in low-middle SDI regions, with an EAPC of −2.08 (95% CI: −2.14 to −2.02). Of note, the ASYR among females in low SDI regions showed a slight increase from 1990 to 2003, followed by a rapid decline thereafter (Figure 1B).

**Figure 2:**
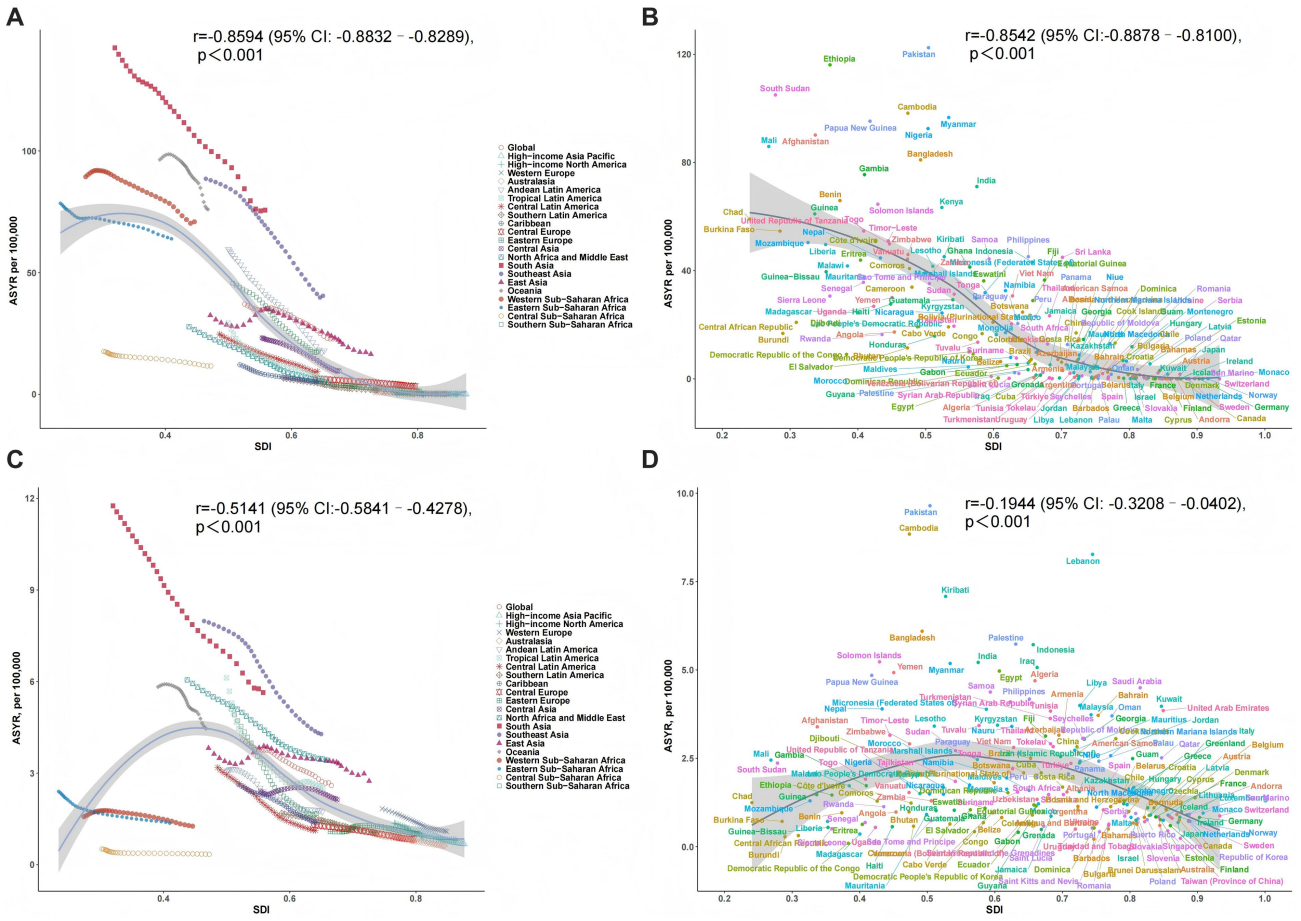
Association between SDI and ASYR for cataract attributable to HAP **(A, B)** and smoking **(C, D)**. **(A, C)** Trends in 21 regions, 1990–2021. **(B, D)** 2021 estimates for 204 countries and territories. The blue line represents the expected ASYR based solely on the SDI. ASYR, age-standardised YLD rate; SDI, Socio-demographic Index; HAP, household air pollution.

At the geographic regional level, for HAP, South Asia had the highest number of YLDs in 2021, reaching 1,030,038.29 (95% UI: 347,130.34–2,022,065.08), followed by East Asia, Southeast Asia, Western Sub-Saharan Africa, and Eastern Sub-Saharan Africa. In 2021, the highest ASYR occurred in Oceania at 75.98 (95% UI: 31.18–142.86) per 100,000, followed by South Asia, Western Sub-Saharan Africa, Eastern Sub-Saharan Africa, and Southeast Asia. From 1990 to 2021, the ASYR of cataracts attributable to HAP decreased across all GBD regions, with the largest reduction observed in Australasia (EAPC –6.45, 95% CI: −6.73 to −6.17) and the smallest reduction in Eastern Sub-Saharan Africa (EAPC –0.57, 95% CI: −0.61 to −0.52) (Table 1). Spearman correlation analysis revealed a significant negative correlation between the ASYR of cataract attributable to HAP and SDI across 21 global regions (*r* = −0.8594, 95% CI: −0.8832 to −0.8289, *p* < 0.001), indicating that regions with lower SDI (e.g., Sub-Saharan Africa) carried a greater disease burden (Figure 2A).

For smoking, South Asia had the highest number of YLDs in 2021, reaching 79,609.34 (95% UI: 56,688.97–110,778.81), followed by East Asia, Southeast Asia, North Africa and Middle East, and Western Europe. In 2021, the highest ASYR occurred in South Asia at 5.64 (95% UI: 4.01–7.85) per 100,000, followed by Oceania, Southeast Asia, North Africa and Middle East, and East Asia. From 1990 to 2021, the ASYR of cataract attributable to smoking decreased across all GBD regions, with the largest reduction observed in Southern Sub-Saharan Africa (EAPC –3.89, 95% CI: −4.09 to −3.68) and the smallest reduction in Eastern Europe (EAPC –0.20, 95% CI: −0.48 to 0.09) (Table 2). Spearman correlation analysis showed a significant negative correlation between the ASYR of cataract attributable to smoking and SDI across the 21 global regions (*r* = −0.5141, 95% CI: −0.5841 to −0.4278, *p* < 0.001) (Figure 2C). Of note, the association exhibited an N-shaped pattern: a positive correlation was observed at an SDI of approximately ≤ 0.45, which shifted to a negative correlation at SDI > 0.45, indicating that regions with low-middle SDI (e.g., South Asia) carried a greater disease burden.

### 3.3 National level

**Figure 3:**
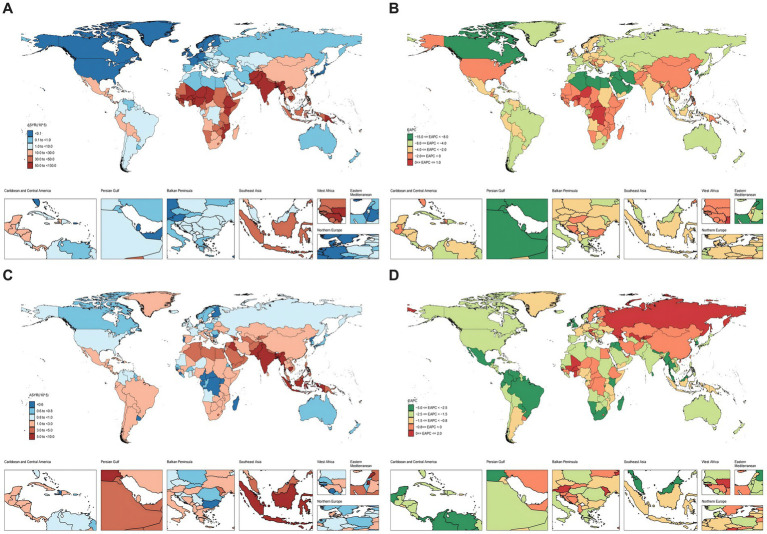
Spatiotemporal distribution maps of cataract attributable to HAP **(A, B)** and smoking **(C, D)** among 204 countries and territories. **(A, C)** ASYR in 2021. **(B, D)** EAPC of ASYR, 1990–2021. ASYR, age-standardised YLD rate; EAPC, estimated annual percentage change; HAP, household air pollution.

For HAP, across 204 countries and territories, SDI was significantly negatively correlated with the cataract burden (*r* = −0.8542, 95% CI: −0.8878 to −0.8100, p < 0.001) (Figure 2B). In 2021, India had the highest number of YLDs at 783,329.99 (95% UI: −267,646.86–1,551,569.17), followed by China, Pakistan, Bangladesh, and Indonesia (Supplementary Table S1). The ASYRs ranged from around 0.02 to 112.45 per 100,000. Among all countries, Pakistan (112.45 per 100,000, 95% UI: −35.33–247.37) had the highest ASYR, with Ethiopia, South Sudan, Cambodia, and Myanmar following (Figure 3A; Supplementary Table S1). Conversely, the United Kingdom (0.02 per 100,000, 95% UI: −0.01–0.07) had the lowest ASYR, followed by Barbados, Sweden, Switzerland, and Norway. The most notable increase in ASYR was in Benin (EAPC 0.62, 95% CI: 0.44–0.80), then Côte d’Ivoire, the Democratic Republic of the Congo, the Central African Republic, and Burkina Faso; Tunisia (EAPC –13.80, 95% CI: −14.26 to −13.35) saw the steepest decline, followed by Egypt, the Syrian Arab Republic, Saudi Arabia, and Oman (Figure 3B; Supplementary Table S1).

For smoking, Spearman correlation analysis showed a significant negative correlation between SDI and ASYR of smoking-attributable cataract across 204 countries and territories (*r* = −0.1944, 95% CI: −0.3208 to −0.0402, *p* < 0.001) (Figure 2D). The association exhibited an N-shaped pattern: positively correlated at SDI ≤ 0.55 and negatively correlated at SDI > 0.55. In 2021, China had the highest number of YLDs at 60,057.78 (95% UI: 41,169.59–84,681.39), followed by India, Indonesia, Pakistan, and Bangladesh (Supplementary Table S2). The ASYRs ranged from around 0.10 to 9.65 per 100,000. Among all countries, Pakistan (9.65 per 100,000, 95% UI: 6.73–13.33) had the highest ASYR, with Cambodia, Lebanon, Kiribati, and Bangladesh following (Figure 3C; Supplementary Table S2). Conversely, the Democratic Republic of the Congo (0.10 per 100,000, 95% UI: 0.06–0.15) had the lowest ASYR, followed by Barbados, Taiwan Province of China, Burundi, and the Central African Republic. The most notable increase in ASYR was in Kyrgyzstan (EAPC 1.77, 95% CI: 1.47–2.07), then Mali, Georgia, Ghana, and Austria; Equatorial Guinea (EAPC –4.50, 95% CI: −4.70 to −4.30) saw the steepest decline, followed by South Africa, Myanmar, Mexico, and Maldives (Figure 3D; Supplementary Table S2).

### 3.4 Age and sex patterns

**Figure 4:**
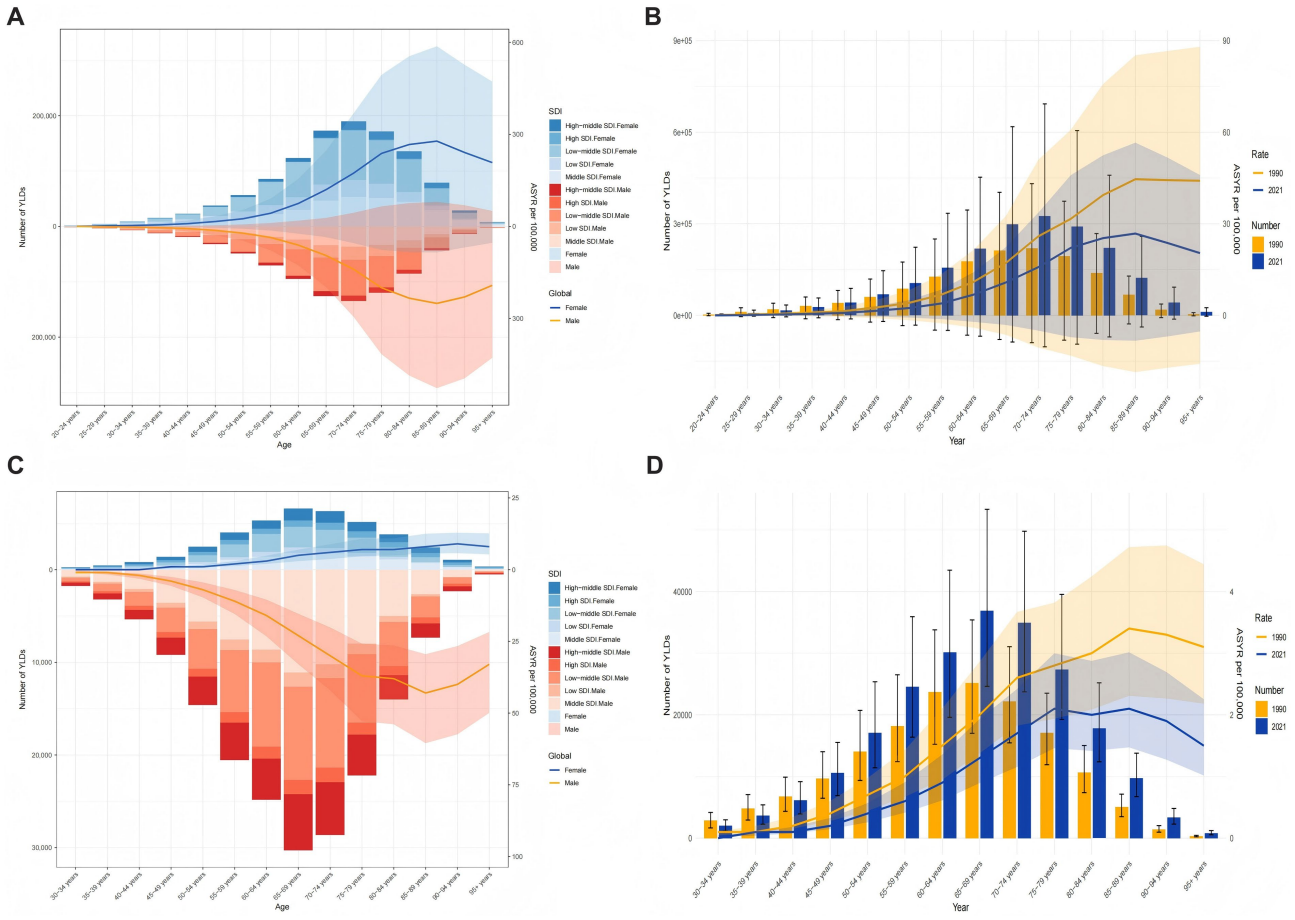
Age-specific YLDs and ASYR of cataract attributable to HAP **(A, B)** and smoking **(C, D)**. **(A, C)** By sex and SDI in 2021. **(B, D)** By year between 1990 and 2021. YLDs: years lived with disability. ASYR, age-standardised YLD rate; HAP, household air pollution; SDI, Socio-demographic Index.

**Figure 5:**
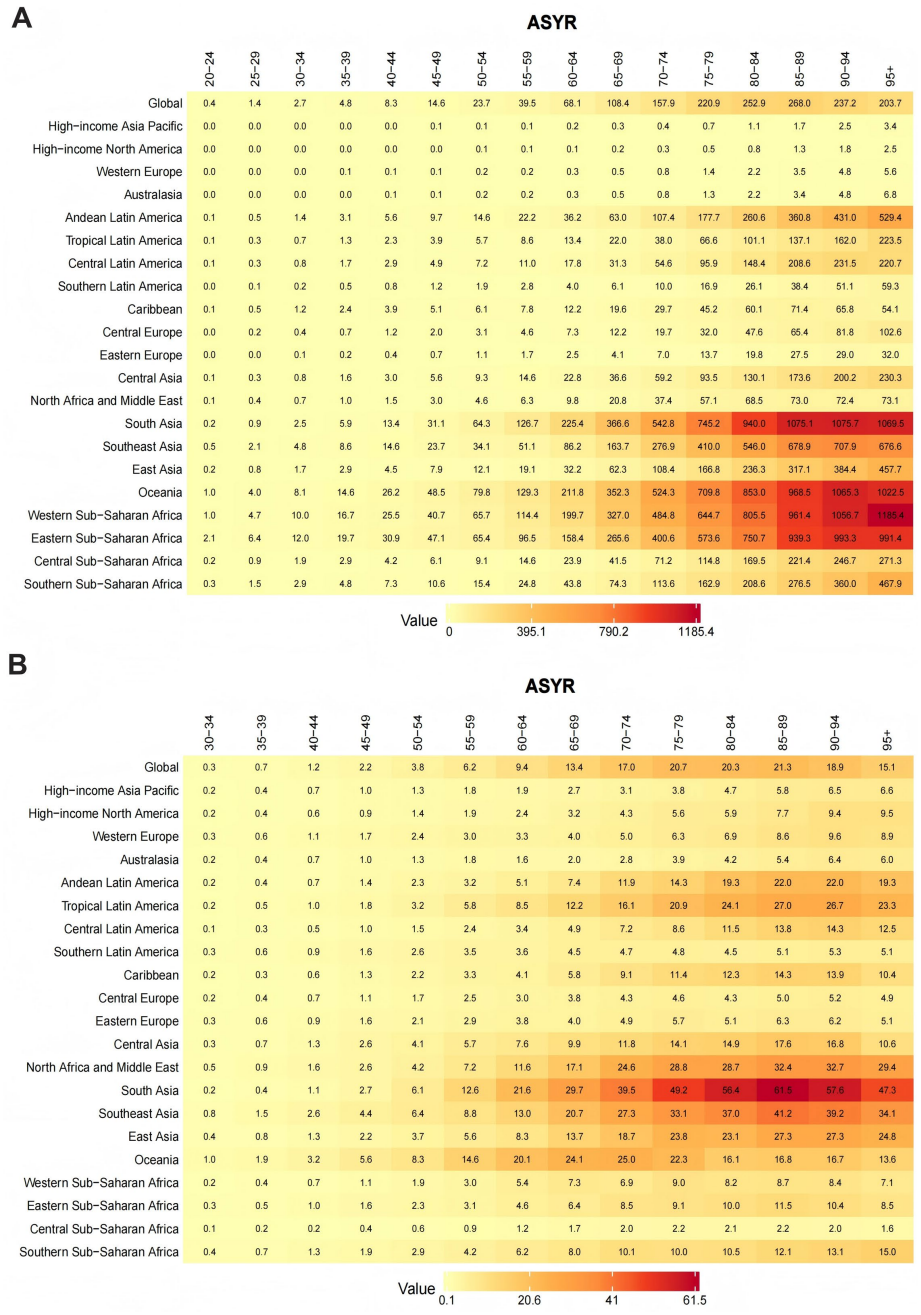
Heatmap of ASYR for cataract attributable to HAP **(A)** and smoking **(B)**, globally and across 21 GBD regions. ASYR, age-standardised YLD rate; HAP, household air pollution.

With respect to age, data on HAP-attributable cataract were limited to individuals aged 20 years and older, and data on smoking-attributable cataract to those aged 30 years and older, between 1990 and 2021 (Figure 4). For HAP, both the number of cataract YLDs and the ASYR increased progressively with age: YLDs peaked in the 70–74 age group, and the ASYR peaked in the 85–89 age group. These data indicate that the HAP-attributable cataract burden occurred primarily in individuals aged 60–84 years. Notably, among all SDI regions, the low-middle SDI regions had the highest proportions of YLDs, and both YLDs and ASYR were slightly higher among females than among males (Figure 4A). The age patterns of YLDs and ASYRs were similar between 1990 and 2021. Compared with 1990, the number of YLDs in 2021 increased substantially across all age groups, whereas ASYRs decreased markedly, with the reduction becoming more pronounced with increasing age (Figure 4B). The heatmap clearly demonstrated that the ASYR increased with age across all GBD regions, with the heaviest burden concentrated in South Asia, Oceania, Western Sub-Saharan Africa, and Eastern Sub-Saharan Africa, where the ASYR among individuals aged 85 years and older exceeded 900 per 100,000 and peaked at a striking 1185.4 per 100,000 in the 95 + age group of Western Sub-Saharan Africa (Figure 5A).

For smoking, both the number of cataract YLDs and the ASYR also increased progressively with age: YLDs peaked in the 65–69 age group, while the ASYR peaked in the 85–89 age group. These findings indicate that the smoking-attributable cataract burden occurred primarily among individuals aged 55–79 years. Notably, among all SDI regions, the low-middle and middle SDI regions had the highest proportions of YLDs, and both YLDs and ASYR were significantly higher among males than among females (Figure 4C). Consistent with HAP, the age patterns of YLDs and ASYRs remained similar between 1990 and 2021, with YLDs increasing substantially across all age groups in 2021 while ASYRs decreased markedly (Figure 4D). As presented in the heatmap, ASYR rose gradually with age across all GBD regions and peaked at 85–89 years of age. The heaviest burden was concentrated in South Asia, where ASYR among individuals aged 80–94 years exceeded 50 per 100,000 and reached a peak of 61.5 per 100,000 in the 85–89 age group (Figure 5B).

### 3.5 Cross-country inequality analysis

**Figure 6:**
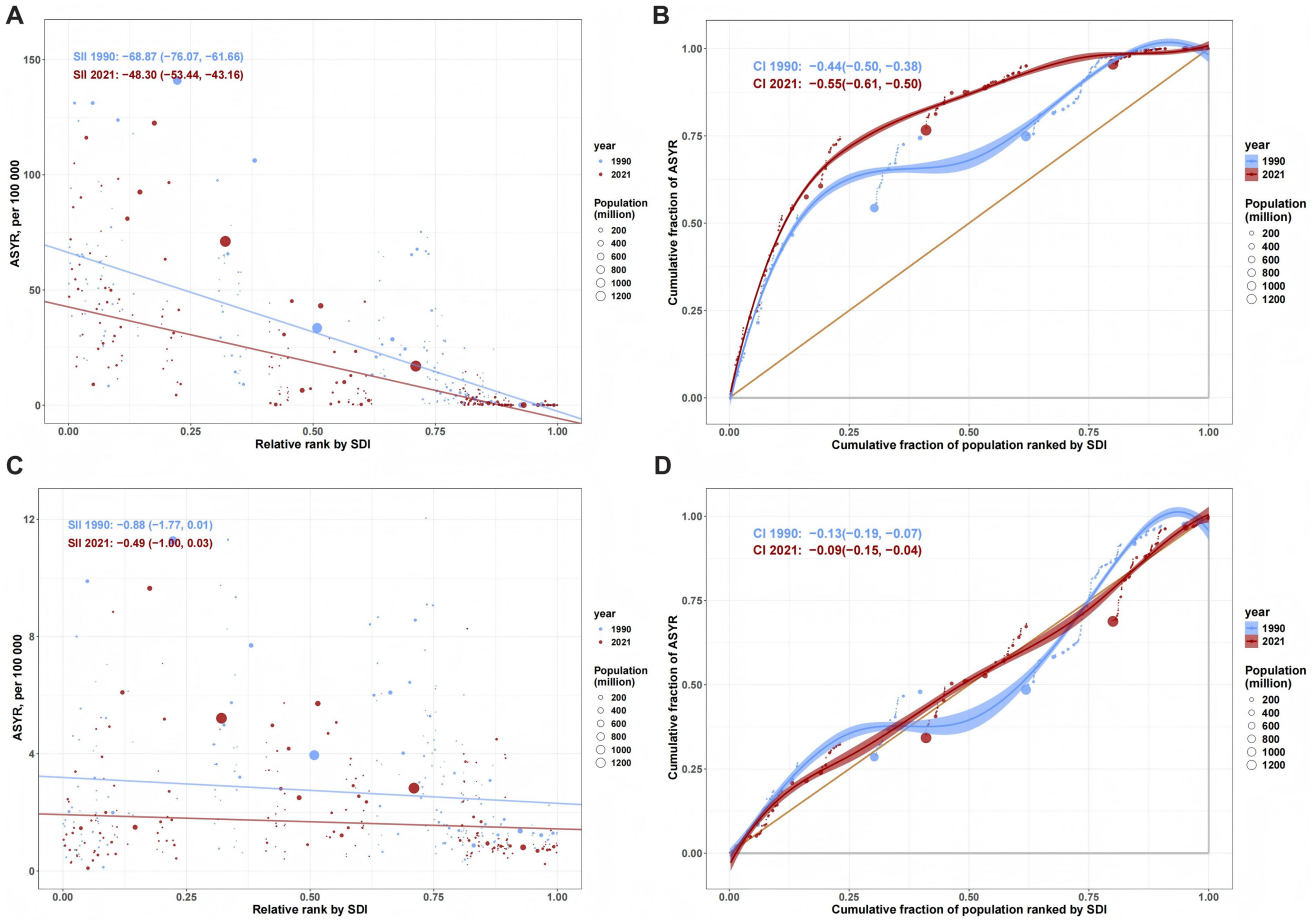
Absolute and relative cross-country inequalities in the ASYR of cataract attributable to HAP **(A, B)** and smoking **(C, D)**, 1990–2021. **(A, C)** Health inequality regression curves. **(B, D)** Concentration curves. ASYR, age-standardised YLD rate; HAP, household air pollution; SDI, Socio-demographic Index.

For cataract burden attributable to either HAP or smoking, both the SII and CI were negative, indicating that countries/territories with lower SDI carried a disproportionately higher burden (Figure 6). For HAP, the SII showed that the ASYR gap decreased from −68.87 (95% CI: −76.07 to −61.66) in 1990 to −48.30 (95% CI: −53.44 to −43.16) in 2021, suggesting a reduction in absolute cross-country health inequalities for HAP-attributable cataract burden. By contrast, the CI for ASYR decreased from −0.44 (95% CI: −0.50 to −0.38) in 1990 to −0.55 (95% CI: −0.61 to −0.50) in 2021, indicating widened relative cross-country health inequalities (Figures 6A,B).

In contrast to HAP, both absolute and relative cross-country health inequalities in smoking-attributable cataract burden improved. For the ASYR, the SII decreased from −0.88 (95% CI: −1.77 to 0.01) in 1990 to −0.49 (95% CI: −1.00 to 0.03) in 2021, while the CI fell from −0.13 (95% CI: −0.19 to −0.07) to −0.09 (95% CI: −0.15 to −0.04) over the same period (Figures 6C,D).

### 3.6 Decomposition analysis

**Figure 7:**
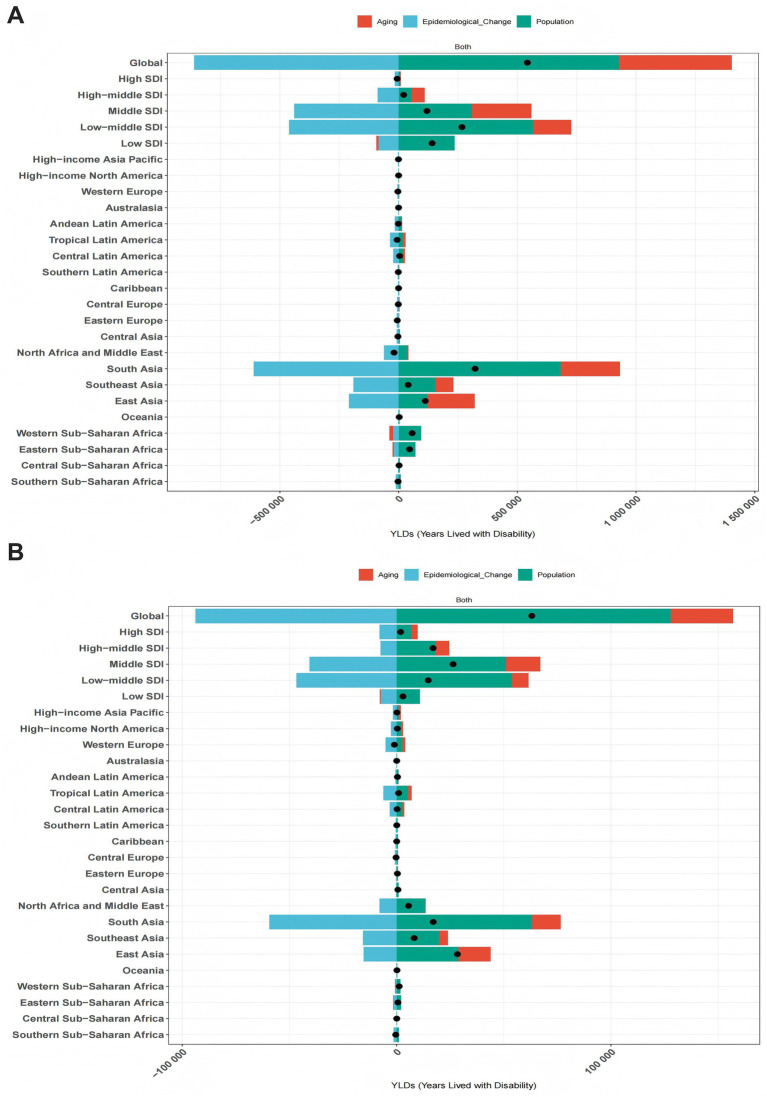
Changes in YLDs for cataract attributable to HAP **(A)** and smoking **(B)**, globally and across 21 GBD regions, 1990–2021 (driven by aging, population growth, and epidemiology shifts). The black dots represent the total change contributed by all three factors. A positive value for each component indicates a positive contribution to YLDs, and a negative value indicates a negative contribution. ASYR, age-standardised YLD rate; HAP, household air pollution; SDI, Socio-demographic Index.

To disentangle the drivers of temporal changes in YLDs for cataract attributable to HAP and smoking, we performed a decomposition analysis to assess the relative impacts of aging, population growth, and epidemiological shifts over the 1990–2021 period (Figure 7; Supplementary Table S3). Globally and across all SDI regions except high SDI regions, HAP-attributable YLDs showed an increasing trend. Aging, population growth, and epidemiological shifts accounted for 87.79, 171.01%, and −158.81% of the global increase in disease burden, respectively. In high-middle SDI regions and East Asia, the contribution of aging to the increase in disease burden (178.21 and 264.13%, respectively) exceeded that of population growth (107.88 and 255.35%, respectively), indicating an aging-dominated increase in burden. In some regions of South Asia, Eastern Sub-Saharan Africa, and Western Sub-Saharan Africa, the contribution of population growth to the increase in disease burden was significantly higher than that of aging, indicating a population growth-dominated increase in burden. In high SDI regions, North Africa and Middle East, Tropical Latin America, Eastern Europe, and other regions, the contribution of epidemiological shifts to the reduction in disease burden was significantly greater than the combined negative contributions of aging and population growth. For example, in North Africa and Middle East, the contributions of aging, population growth, and epidemiological shifts to the reduction in disease burden were −32.48, −182.03, and 314.52%, respectively (Figure 7A; Supplementary Table S3).

For smoking, YLDs showed an increasing trend globally and across all SDI regions. Aging, population growth, and epidemiological shifts accounted for 46.31, 202.47, and −148.78% of the global increase in disease burden, respectively. In most regions, the contribution of population growth to the increase in disease burden was significantly higher than that of aging, indicating a population growth-dominated increase in burden. In Western Europe, Southern Sub-Saharan Africa, and Central Europe, the contribution of epidemiological shifts to the reduction in disease burden was greater than the combined negative contributions of aging and population growth. For example, in Western Europe, the contributions of aging, population growth, and epidemiological shifts to the reduction in disease burden were −142.21, −264.37, and 506.58%, respectively (Figure 7B; Supplementary Table S4).

### 3.7 Frontier analysis

**Figure 8:**
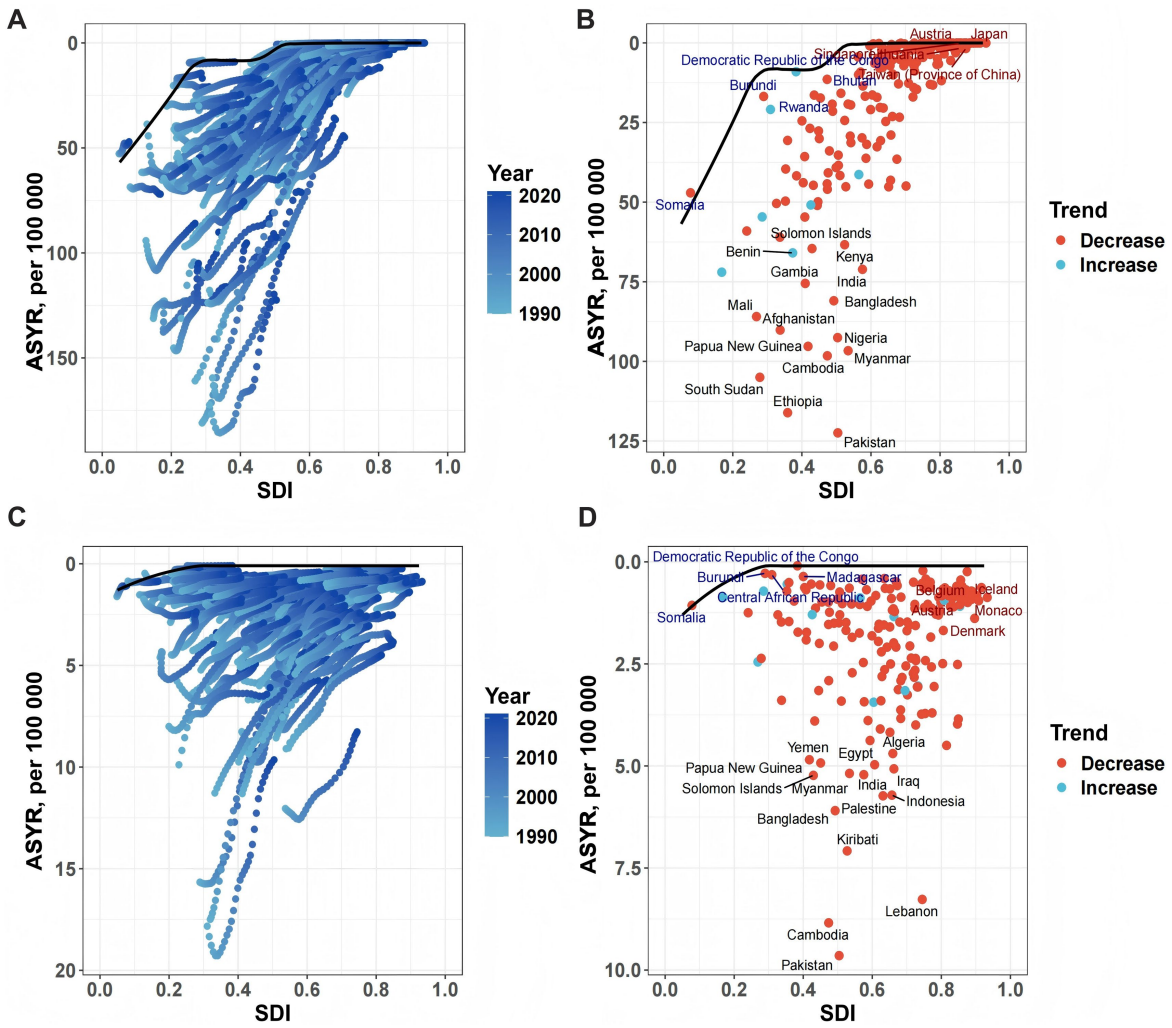
Frontier analysis exploring the relationship between the SDI and ASYR of cataract attributable to HAP **(A, B)** and smoking **(C, D)** in 204 countries and territories. **(A, C)** Light blue (1990) to dark blue (2021) indicate the change over time. The frontier line delineates the countries and territories with the lowest ASYRs (optimal performers) given their SDIs. **(B, D)** Each point represents a specific country or territory in 2021, the frontier line is shown in black, and the top 15 countries and territories with the largest efficiency differences from the frontier are marked in black. The direction of the change in the ASYR from 1990 to 2021 is indicated by the color of the dots, with red dots representing decreases and blue dots representing increases. ASYR, age-standardised YLD rate; HAP, household air pollution; SDI, Socio-demographic Index.

To explore the optimal scenario for minimizing disease burden relative to SDI across 204 countries and territories, we performed a frontier analysis. The efficiency difference at a given SDI generally decreased with increasing global SDI (Figure 8). For HAP, the 15 countries with the largest actual differences (efficiency difference range: 0–118.89 per 100,000 population) included Pakistan (efficiency difference = 118.89), Ethiopia (107.74), South Sudan (96.16), Myanmar, Cambodia, Nigeria, Papua New Guinea, Afghanistan, Mali, Bangladesh, India, Gambia, Kenya, Benin, and Solomon Islands. These nations were predominantly located in low and low-middle SDI regions, mainly across South Asia and sub-Saharan Africa. The observed ASYRs in these countries were markedly higher than the theoretical optimal values (frontier) at the corresponding SDI level, implying a considerable avoidable disease burden in these nations. The ASYRs of high-income countries such as Barbados (0.0004), Switzerland (0.004), and Sweden (0.004) were highly consistent with the theoretical frontier, reflecting the high efficiency of their health resource allocation (Figure 8A; Supplementary Table S5).

For smoking, the 15 countries with the largest actual differences (efficiency difference range: 0–9.55 per 100,000 population) included Pakistan (9.55), Cambodia (8.75), Lebanon (8.17), Kiribati, Bangladesh, Palestine, Indonesia, Solomon Islands, India, Myanmar, Iraq, Egypt, Yemen, Papua New Guinea, and Algeria. These nations were predominantly located in low-middle SDI regions, mainly across South Asia, Southeast Asia, North Africa and Middle East, and Oceania. Notably, countries in low SDI regions did not exhibit large efficiency differences; for instance, Burundi (0.18), the Central African Republic (0.21), and Madagascar (0.26) showed actual performances close to the optimal level given their socioeconomic conditions (Figure 8B; Supplementary Table S6).

### 3.8 Projections up to 2050 based on ARIMA model

All six fitted ARIMA models passed the Ljung–Box white noise test (*p* > 0.05), suggesting no statistically significant autocorrelation remained in model residuals and each model adequately captured the temporal patterns of cataract-related YLDs time series (Table 3). All three HAP-related models required second-order differencing (*d* = 2), indicating that the cataract YLD rates attributable to HAP featured varying long-term trends and non-stationarity. The male-specific HAP model was ARIMA(2,2,0) (AIC = −53.63) with a second-order autoregressive structure, meaning current values depended on observations from the preceding 2 years. The female and combined population HAP models took the ARIMA (2) specification with additional moving-average terms, reflecting more complex short-term random fluctuations. Ljung–Box *p*-values of all three models exceeded 0.92, verifying complete extraction of systematic temporal patterns. Smoking-associated models exhibited simpler structures. The male and pooled smoking models were ARIMA(0,1,1) with drift and ARIMA(1,1,0) with drift, respectively, indicating a deterministic linear trend. The female smoking model adopted ARIMA(0,2,0) (AIC = −194.68) without drift, suggestive of a stochastic rather than deterministic trend. AIC values of smoking-related models ranged from −194 to −111, substantially lower than those of HAP models (−53 to −32), which reflected a higher signal-to-noise ratio and a more distinct declining trend for smoking-attributable cataract YLDs (Table 3).

**Table 3:** Specification and fitting statistics of ARIMA models for cataract ASYR attributable to HAP and smoking stratified by sex, 1990–2021.

Risk factors	Sex	ARIMA Models (*p*, *d*, *q*)	AIC	BIC	logLik	Ljung–Box *Q*	*p* value
HAP	Male	ARIMA(2,2,0)	−53.63	−49.43	29.81	1.939	0.9252
HAP	Female	ARIMA(2,2,2)	−32.42	−25.42	21.21	1.106	0.9813
HAP	Both	ARIMA(2,2,2)	−40.92	−33.91	25.46	1.923	0.9267
Smoking	Male	ARIMA(0,1,1) w/drift	−111.74	−107.43	58.87	2.489	0.8697
Smoking	Female	ARIMA(0,2,0)	−194.68	−193.28	98.34	1.932	0.9258
Smoking	Both	ARIMA(1,1,0) w/drift	−144.80	−140.50	75.40	1.503	0.9593

ASYR, age standardized YLD rate; HAP, household air pollution; ARIMA, Autoregressive Integrated Moving Average; AIC, Akaike information criterion; BIC, Bayesian information criterion; logLik, Log-likelihood; w/ drift, With drift.

**Figure 9:**
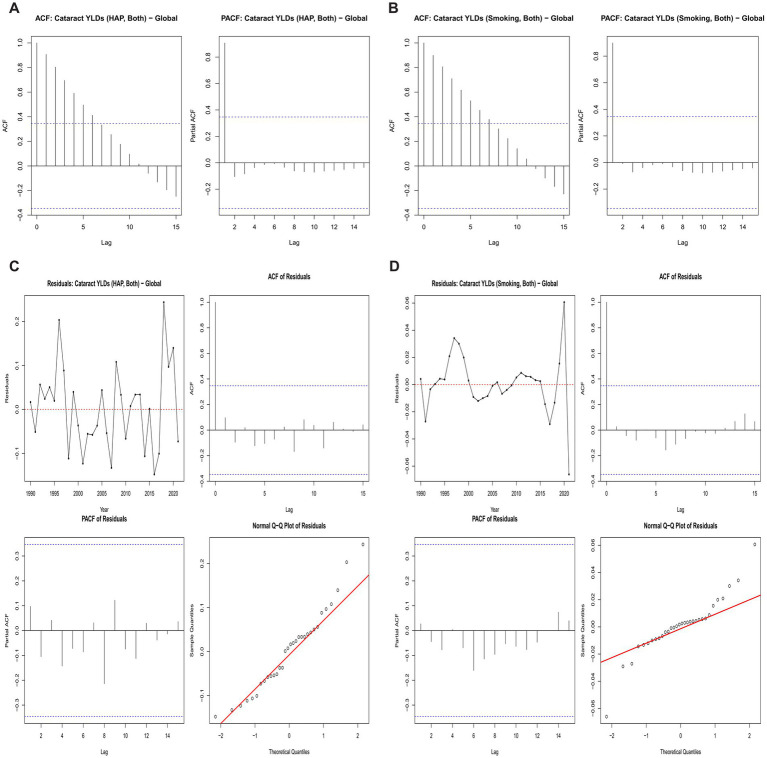
ACF and PACF plots **(A, B)** and residual diagnostic panels **(C, D)** for global ASYR time series of cataract attributable to HAP **(A, C)** and smoking **(B, D)**, 1990–2021. All residual lags fall within the 95% confidence bounds (blue dashed lines), confirming white noise distribution and adequate model fitting. Residual diagnostics in panels **C** and **D** consist of residual time-series plot, residual ACF, residual PACF, and normal *Q*–*Q* plot sequentially. ASYR, age-standardised YLD rate; HAP, household air pollution; ACF, autocorrelation function; PACF, partial autocorrelation function.

**Figure 10:**
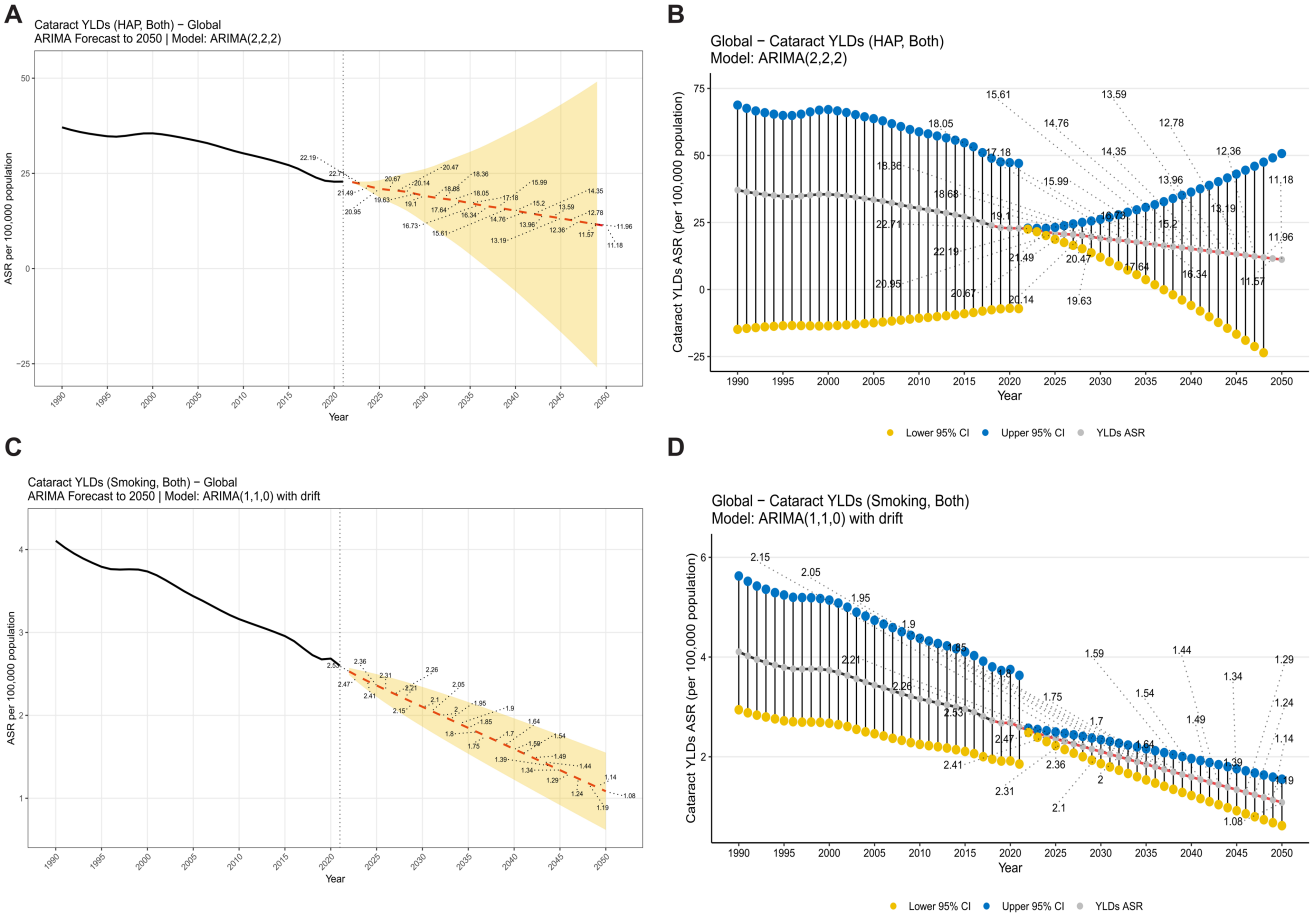
ARIMA models were applied to project trends in cataract ASYR attributable to HAP **(A, B)** and smoking **(C, D)** from 1990 to 2050. Panels A and C show overall temporal trends, while Panels B and D present detailed uncertainty information. ASYR, age-standardised YLD rate; HAP, household air pollution; ARIMA, autoregressive integrated moving average.

For HAP-attributable cataract YLD series, the ACF decayed gradually with oscillatory tailing, whereas the PACF presented significant coefficients at short lags and truncated rapidly within the 95% confidence bounds at longer lags (Figure 9A). For smoking-related counterparts, the ACF likewise declined slowly with tail-off, and the PACF converged quickly into confidence bands after prominent early-lag significance (Figure 9B). These patterns revealed mixed ARMA characteristics for both time-series datasets, thereby supporting the rationality of ARIMA specification. All residual autocorrelation coefficients across lags 0–15 for the two exposure sources fell within the 95% confidence limits without any statistically significant spikes. Residuals fluctuated randomly around zero, with no persistent upward/downward trends, clustered swings or apparent heteroscedasticity; their mean values approximated zero consistently, satisfying the zero-mean and constant-variance properties of white noise. Sample quantiles closely aligned with the theoretical normal reference line without substantial deviation in the *Q*–*Q* plots, confirming approximately normally distributed residuals and validating the normality assumption required for robust ARIMA parameter estimation (Figures 9C,D). ARIMA models stratified by sex (male and female) for both risk factors also met the goodness-of-fit criteria, as detailed in Supplementary Figures S1 and S2. The global ASYRs of cataract attributable to both HAP and smoking shows a significant downward trend across both sexes (Figure 10; Supplementary Figures S3 and S4). Specifically, the global ASYRs of cataract attributable to HAP and smoking are expected to decrease from 22.71 (95% UI: 22.51–22.91) and 2.53 (95% UI: 2.49–2.57) per 100,000 individuals in 2022 to 11.18 (95% UI: −28.34–50.70) and 1.08 (95% UI: 0.62–1.55) per 100,000 by 2050, corresponding to cumulative reductions of 50.77 and 57.31%, respectively (Figure 10; Supplementary Table S7).

## 4 Discussion

### 4.1 Global trends and regional disparities

This study comprehensively analysed the global cataract burden attributable to HAP and smoking from 1990 to 2021 and projected its future trajectory to 2050, filling the gap in global-scale, long-term, and multi-dimensional analyses of these two modifiable environmental risk factors. In 2021, globally, there were approximately 1.95 million and 220,000 cataract YLDs attributable to HAP and smoking, respectively, with ASYRs of 22.84 and 2.60 per 100,000 population. Consistent with the declining ASYRs, the global EAPC for HAP- and smoking-attributable cataract were significantly negative (−1.51 and −1.41, respectively), indicating a steady decrease in age-standardized burden over the study period. However, the decreasing pace varied across regions, with faster declines in high SDI areas and slower reductions in low SDI regions, which may further exacerbate existing health disparities. Notably, despite declining ASYR, the absolute YLDs increased in nearly all regions, rising by nearly 40% globally from 1990 to 2021 and inevitably increasing societal medical and economic burdens. This paradox—falling age-standardized rates but rising absolute counts—can be primarily explained by global demographic changes, particularly population growth and aging, consistent with decomposition analysis findings. In contrast, epidemiological shifts (e.g., improved household air quality, tobacco control policies) mitigated the burden. This suggests that while global efforts to control these risk factors have reduced per capita burden (18, 44, 53), population growth and aging have offset these gains, leading to an overall increase in absolute disease burden, highlighting the urgency of targeted interventions to address both demographic and environmental risks.

For HAP, the highest ASYR occurred in low SDI regions, while the lowest ASYR was observed in high SDI regions. Spearman analysis revealed a significant negative correlation between cataract ASYRs and SDI across 21 GBD regions. This finding highlights the persistent socioeconomic disparities in the global burden of cataract attributable to HAP. The elevated burden in low SDI regions may be driven by multiple interconnected factors, including limited access to clean household fuels and ventilation, weak implementation of environmental health policies, and insufficient healthcare resources for cataract prevention and treatment (54–56). In contrast, high SDI regions benefit from greater access to clean energy, improved household air quality, well-established healthcare systems, and more effective public health interventions, all of which contribute to lower ASYR of HAP-attributable cataract (57). Regionally, South Asia, Oceania, Sub-Saharan Africa, and Southeast Asia bear the heaviest HAP-attributable cataract burden, with South Asia having the highest number of YLDs and Oceania having the highest ASYR. These findings are consistent with those of previous studies on HAP exposure (57). Alarmingly, the ASYR among older adult cataract patients aged over 70 years in these regions reaches 200–500 times the global average, underscoring an urgent need for targeted public health interventions focusing on older adult populations in high-burden regions. Populations in these regions remain at the bottom of the energy ladder, with widespread reliance on traditional solid fuels due to economic constraints and inadequate clean energy infrastructure (58, 59). Concurrently, shortages in healthcare and cataract surgical capacity further compound the overall disease burden (60).

For smoking, the regional distribution of the burden was slightly different. The highest number of YLDs shifted from low-middle SDI regions in 1990 to middle SDI regions in 2021, and the low-middle SDI regions still had the highest ASYR in 2021. The correlation between SDI and smoking-attributable ASYRs was negative but showed an N-shaped pattern: positive at SDI ≤ 0.45 and negative at SDI > 0.45. This indicates that smoking-related burden rises with socioeconomic development in low-SDI regions but declines in middle and high-SDI regions owing to stronger tobacco control and improved health awareness. Notably, South Asia carried the heaviest burden of smoking-attributable cataract, with the highest YLDs and ASYR globally. Though its regional disparity is less pronounced than that seen in HAP-related disease burden, the ASYR of smoking-related cataract patients aged over 70 years in South Asia is approximately 20–30 times the global average. Smoking is highly prevalent in South Asia, where tobacco use is deeply embedded in social and cultural norms, and is more common among men with low socioeconomic status, low education levels, and rural residents in South Asian countries (61, 62). South Asia has achieved a marked reduction in smoking-attributable cataract burden (EAPC = −2.38), indicating effective tobacco control and expanded cataract surgical coverage (63–65). However, South Asia remains a high-priority region for targeted interventions, not only because of high smoking prevalence among youth, but also due to its persistently high overall burden (66–68).

### 4.2 Sex and age disparities

The burden of HAP- and smoking-attributable cataract showed distinct sex patterns: females bore a slightly higher HAP-related burden, while males consistently had a higher smoking-related burden. This gap might stem from the interaction between social behavioral factors and biological susceptibility. First, women in resource-poor countries are much more heavily exposed to HAP, as they spend far more time cooking and heating with biomass fuels in inefficient traditional stoves (69). Conversely, smoking is far more prevalent among men, exposing them to numerous toxic substances and thereby increasing the risk of cataract development (33, 34, 44, 70). Second, biological and ocular structural disparities contribute substantially to the higher cataract susceptibility in females. Females consistently exhibit shorter axial length, shallower anterior chamber depth, and thinner lenses, anatomical features that increase vulnerability to oxidative stress and ultraviolet radiation (71, 72). Concurrently, the human lens expresses estrogen receptors, and 17β-estradiol exerts sex-specific protection against cataractogenesis in females—a protective effect lost following menopause, collectively accelerating lens opacification (73).

Notably, these findings—combined with the aforementioned biological mechanisms—may lead to an underappreciation of the urgency and inequality related to the female cataract burden. According to GBD 2021, females had significantly higher numbers of cataract cases and higher age-standardized prevalence rates than males (3). However, the effective cataract surgical coverage (eCSC) was substantially lower in females than in males (60). Moreover, a rising trend in smoking prevalence has been observed among women worldwide in recent years (74), and hidden female smoking is often overlooked (75). These observations suggest that the HAP- and smoking-attributable cataract burden among females may be even more severe than currently estimated.

Our findings further demonstrated that the YLDs and ASYRs of cataract burden attributable to HAP and smoking generally rose with advancing age and were predominantly concentrated in middle-aged and older individuals. This phenomenon arises from the progressive nature of cataract, whose severity increases gradually with age as the non-regenerative crystalline lens undergoes lifelong cumulative oxidative stress, protein denaturation and aggregation, and compaction of aged fibers, resulting in irreversible opacification and visual impairment (76). Notably, the peak burden caused by smoking appears earlier than that attributed to HAP, which may be related to differences in population exposure characteristics and intensity. Several studies have demonstrated a dose–response relationship between smoking and cataract, with smokers developing cataracts earlier than non-smokers (77, 78).

### 4.3 Health inequalities, avoidable burden, and policy implications

Cross-country health inequality analysis revealed that lower SDI regions continue to bear a disproportionate cataract burden attributable to both HAP and smoking, highlighting that environmental risk factors exert a disproportionately greater impact on economically disadvantaged areas and underscoring the ongoing challenge of achieving global health equity. For HAP-related cataract, absolute inequality has decreased, but relative inequality has widened, likely driven by faster improvements in high SDI regions and persistent gaps in low SDI areas. In contrast, inequalities related to smoking have consistently improved, reflecting the effectiveness of global tobacco control policies and public health interventions, indicating that sustained policy action can successfully narrow socioeconomic health disparities. These findings highlight the need to prioritize low SDI regions in efforts to expand access to clean energy, improve household ventilation, and strengthen environmental health regulations, alongside continued tobacco control and investments in eye care infrastructure to achieve more equitable and sustainable health outcomes globally.

Decomposition analysis revealed that, to effectively address the cataract burden, differentiated prevention and control policies should be formulated based on the dominant driving factors of each region, with the aim of reducing the disease burden and promoting global health equity. For aging-dominated regions (e.g., East Asia), efforts should focus on strengthening early cataract screening, optimizing the allocation of ophthalmic resources, and improving medical accessibility for the older adults to reduce avoidable visual impairment. For population growth-driven regions (e.g., South Asia), priority should be given to expanding primary eye care coverage and popularizing basic prevention knowledge to achieve early detection and intervention. Regions with significant epidemiological improvements (e.g., Western Europe) provide valuable references—their prevention models and implementation pathways can be summarized to offer replicable experiences for other regions to optimize their strategies.

Frontier analysis confirmed significant avoidable burdens of HAP- and smoking-attributable cataract. For HAP-related cataract, substantial inefficiencies clustered in lower SDI regions, notably South Asia and sub-Saharan Africa. Countries like Pakistan and Ethiopia showed actual ASYRs far exceeding the optimal frontier, indicating considerable avoidable burden, likely driven by limited clean energy access, poor infrastructure, and scarce eye care resources. In contrast, high-income nations such as Switzerland and Sweden approached the frontier, reflecting efficient resource allocation and effective policies. For smoking-related cataract, largest gaps were concentrated in low-middle SDI regions (e.g., South Asia, Southeast Asia), exemplified by Pakistan and Cambodia (79). Notably, low SDI countries like Burundi and Madagascar demonstrated minimal inefficiencies, performing close to optimal given their constraints. This suggests that while structural barriers hinder progress in reducing HAP-related burden, the lower smoking-attributable cataract burden observed in low SDI regions likely reflects a complex interplay of effective tobacco control interventions and economic constraints limiting tobacco access.

Fortunately, ARIMA projections indicate that the global absolute burden of HAP- and smoking-attributable cataract will keep decreasing. Therefore, long-term sustained interventions are still required to facilitate such favorable declines. The majority of HAP- and smoking-attributable cataract cases are preventable, and targeted measures are urgently needed to mitigate their burden. First, for HAP-related cataract, low SDI regions (including South Asia, Sub-Saharan Africa, and Oceania) should prioritize clean energy transitions through targeted subsidies to replace solid fuels, paired with free or low-cost ventilation upgrades for vulnerable households. Environmental health regulations should be strengthened in low and low-middle SDI settings, with regular air quality monitoring and enforcement against high-pollution fuels in dense areas. Primary eye care services must be expanded in burdened regions, including training grassroots staff for basic screening and deploying mobile clinics to remote areas. Public health campaigns should also target high-exposure groups (particularly women) to raise awareness and promote safe cooking and heating practices. Second, for smoking-related cataract, middle and low-middle SDI regions, especially South Asia, need robust tobacco control measures. These include raising tobacco taxes, expanding smoke-free environments, and restricting advertising, with a sharp focus on youth and rural populations. Primary healthcare facilities should offer free or low-cost smoking cessation support, integrated into routine eye care. Health education should emphasize the smoking-cataract link for low education groups, while gender-specific initiatives are needed to address hidden female smoking. Finally, tobacco control should be embedded into national eye health strategies, with dedicated funding for high-burden regions.

### 4.4 The strengths and limitations

The present study has several notable strengths. First, we firstly evaluated HAP and smoking as two critical modifiable risk factors for cataract burden. Second, based on the GBD 2021 dataset, we provided a comprehensive global, regional, and national assessment, offering updated population-based evidence. Third, multiple rigorous analytical approaches, including decomposition, inequality, frontier, and ARIMA forecasting analyses, were jointly applied to systematically explore the temporal drivers, disparities, and future trends of cataract burden. For ARIMA projection specifically, comprehensive model validation was rigorously implemented via ACF and PACF identification for model order selection, as well as full residual diagnostic tests including Ljung–Box white-noise verification and residual normality checking to guarantee robust forecasting performance. Fourth, detailed stratified interpretations across sex, age, and SDI subgroups highlighted the heterogeneous epidemiological characteristics. Finally, our findings provide actionable evidence for global tobacco control and clean household energy strategies, offering practical implications for reducing the environmental-attributable cataract burden.

Notwithstanding these strengths, this study has several limitations. First, this analysis relied on GBD-derived population-level modeled estimates instead of individual primary exposure data, with all findings interpreted at the ecological population level. The comparative risk assessment model also entails inherent uncertainty in cataract-related exposure attribution, while unmeasured confounders such as genetic predispositions and socioeconomic status may bias the observed epidemiological trends. Second, data quality and accessibility vary across countries; incomplete epidemiological records and underreporting of cataract cases in low- and middle-income countries may reduce estimation accuracy and underestimate the true disease burden. Third, although HAP and smoking may exert co-exposure and synergistic effects, this study only quantified their individual attributable burdens without exploring their interactions, potentially underestimating the actual disease burden. Fourth, the 95% prediction intervals widened nonlinearly with extended forecast horizons, reflecting inherent limitations when extrapolating a 32-year training dataset over 29 future years. Notably, the HAP-related models yielded negative lower interval bounds in the long run, which were epidemiologically implausible since rate values cannot drop below zero. This represents a well-documented limitation of linear ARIMA when modelling bounded rate outcomes; log-transformed ARIMA or ETS models could serve as alternative approaches in further research. Fifth, this study only conducted cross-national comparisons and failed to investigate subnational epidemiological heterogeneity. In addition, despite the official release of the updated GBD 2023 dataset, relevant analyses were not performed due to the absence of authorized collaborator access permissions.

## 5 Conclusion

In conclusion, this study used GBD 2021 data to systematically assess the global, regional, and national burden of cataract attributable to HAP and smoking, including its temporal trends, socioeconomic disparities, and future projections. Globally, the age-standardized burden of cataract attributable to these two modifiable environmental risks has decreased. However, pronounced gender and geographic disparities persist: females face a higher burden of HAP-related cataract (with potential underestimation), whereas males bear a greater burden of smoking-related cataract. South Asia, particularly Pakistan, exhibits extremely high cataract burden attributable to both risk factors, representing a critical hotspot for environment-related visual health inequities. Regions with low SDI also sustain disproportionate disease burdens, further widening global health gaps. By integrating multiple analytical methods to independently evaluate two major environmental risk factors, this study addresses limitations of previous single-factor or localized studies and provides updated global epidemiological evidence. These findings support visual health prevention, environmental risk management, and global health equity improvement. To mitigate the rising cataract burden, targeted interventions should prioritize vulnerable groups, including women, older adults, and populations in low- and middle-SDI regions. Integrated strategies promoting clean household energy, strict tobacco control, and accessible eye care are essential to reduce environmental cataract prevalence and advance equitable global visual health.

## Data Availability

The original contributions presented in the study are included in the article/Supplementary material, further inquiries can be directed to the corresponding author.
