# Supplementary material for: Global, regional, and national cataract burden attributable to household air pollution and smoking (1990–2021) and projection to 2050
Source: Front Public Health. 2026 Jul 1;14:1842269. doi: 10.3389/fpubh.2026.1842269 (PMC13369277; doi:10.3389/fpubh.2026.1842269)
Supplement: Supplementary file 1 [file Data_Sheet_1.DOCX]

Supplementary Materials

**Table S1.** The YLDs, ASYR and EAPC (1990–2021) of cataract burden attributable to household air pollution (HAP) among 204 countries and territories.

| **Location** | **1990** | | **2021** | | **1990–2021** |
| --- | --- | --- | --- | --- | --- |
|  | **Number,**  **(95% UI)** | **ASYR**  **per 100 000,**  **(95% UI)** | **Number,**  **(95% UI)** | **ASYR**  **per 100 000,**  **(95% UI)** | **EAPC, %,**  **(95% CI)** |
| Afghanistan | 7423.04 ( -2865.99 - 13857.31 ) | 123.43 ( -48.28 - 228.83 ) | 7428.57 ( -2398.78 - 14627.85 ) | 90.17 ( -29.31 - 175.7 ) | -0.76(-1.05 to -0.47) |
| Albania | 205.6 ( -75 - 400.36 ) | 11.12 ( -4.1 - 21.57 ) | 223.81 ( -39.68 - 545.43 ) | 5.24 ( -0.93 - 12.86 ) | -2.83(-3.08 to -2.59) |
| Algeria | 1464.94 ( -154.45 - 4725.54 ) | 13.33 ( -1.39 - 42.68 ) | 167.46 ( -14.47 - 594.62 ) | 0.51 ( -0.04 - 1.85 ) | -10.96(-11.39 to -10.53) |
| American Samoa | 5.34 ( -0.84 - 13.41 ) | 28.23 ( -4.55 - 70.49 ) | 5.95 ( -0.79 - 17.36 ) | 14.04 ( -1.89 - 41.02 ) | -1.96(-2.1 to -1.82) |
| Andorra | 0.11 ( -0.01 - 0.38 ) | 0.21 ( -0.02 - 0.71 ) | 0.09 ( -0.01 - 0.32 ) | 0.05 ( 0 - 0.2 ) | -4.46(-4.69 to -4.23) |
| Angola | 1383.96 ( -577.53 - 2566.42 ) | 41.4 ( -18 - 77.16 ) | 1758.57 ( -377.86 - 3844.64 ) | 17.31 ( -4.06 - 36.58 ) | -2.89(-3.12 to -2.66) |
| Antigua and Barbuda | 1.74 ( -0.21 - 5.72 ) | 3.03 ( -0.36 - 10.1 ) | 0.68 ( -0.07 - 2.48 ) | 0.7 ( -0.07 - 2.56 ) | -4.47(-4.65 to -4.29) |
| Argentina | 1542.76 ( -219.65 - 4428.32 ) | 5.11 ( -0.73 - 14.71 ) | 724.82 ( -83.71 - 2507.63 ) | 1.26 ( -0.15 - 4.36 ) | -4.81(-4.91 to -4.7) |
| Armenia | 356.7 ( -51.11 - 929.41 ) | 14.54 ( -2.11 - 37.85 ) | 134.73 ( -14.82 - 435 ) | 3.11 ( -0.35 - 10.07 ) | -6.1(-6.74 to -5.44) |
| Australia | 225.35 ( -24.91 - 718.62 ) | 1.2 ( -0.13 - 3.81 ) | 79.19 ( -8.98 - 282.22 ) | 0.17 ( -0.02 - 0.59 ) | -6.62(-6.9 to -6.34) |
| Austria | 37.68 ( -4.16 - 128.35 ) | 0.31 ( -0.03 - 1.08 ) | 19.58 ( -2.08 - 74.84 ) | 0.1 ( -0.01 - 0.37 ) | -3.88(-3.99 to -3.76) |
| Azerbaijan | 1263.09 ( -331.62 - 2625.98 ) | 28.09 ( -7.47 - 58.47 ) | 631.16 ( -91.02 - 1817.86 ) | 7.15 ( -1.05 - 20.76 ) | -5.26(-5.58 to -4.94) |
| Bahamas | 4.84 ( -0.56 - 14.76 ) | 3.43 ( -0.39 - 10.45 ) | 3.11 ( -0.34 - 10.41 ) | 0.86 ( -0.1 - 2.86 ) | -4.62(-4.69 to -4.56) |
| Bahrain | 20.07 ( -2.5 - 60.06 ) | 14.31 ( -1.86 - 42.28 ) | 7.41 ( -0.74 - 27.61 ) | 1.03 ( -0.11 - 3.79 ) | -8.66(-8.79 to -8.53) |
| Bangladesh | 55057.83 ( -25561.48 - 98851.54 ) | 131.15 ( -61.4 - 235.26 ) | 105517.61 ( -38791.95 - 201202.19 ) | 80.97 ( -29.87 - 153.71 ) | -1.14(-1.31 to -0.97) |
| Barbados | 0.32 ( -0.03 - 1.12 ) | 0.1 ( -0.01 - 0.36 ) | 0.14 ( -0.02 - 0.53 ) | 0.03 ( 0 - 0.1 ) | -4.07(-4.17 to -3.96) |
| Belarus | 299.12 ( -34.26 - 968.05 ) | 2.39 ( -0.28 - 7.71 ) | 81.48 ( -8.37 - 303.56 ) | 0.51 ( -0.05 - 1.87 ) | -5.52(-5.87 to -5.18) |
| Belgium | 47.55 ( -4.98 - 167.77 ) | 0.31 ( -0.03 - 1.1 ) | 15.66 ( -1.66 - 57.94 ) | 0.06 ( -0.01 - 0.23 ) | -5.38(-5.57 to -5.19) |
| Belize | 12.74 ( -2.29 - 30.53 ) | 13.82 ( -2.49 - 33.16 ) | 17.25 ( -2.66 - 47.17 ) | 6.37 ( -0.99 - 17.5 ) | -2.45(-2.58 to -2.33) |
| Benin | 948.95 ( -436.39 - 1794.39 ) | 52.66 ( -24.42 - 99.91 ) | 3078.76 ( -1407.76 - 5586.29 ) | 65.93 ( -30.44 - 120.72 ) | 0.62(0.44 to 0.8) |
| Bermuda | 2.55 ( -0.31 - 7.52 ) | 4.32 ( -0.53 - 12.67 ) | 1.68 ( -0.21 - 5.52 ) | 1.16 ( -0.14 - 3.86 ) | -4.29(-4.39 to -4.18) |
| Bhutan | 85.27 ( -32.35 - 158.87 ) | 45.2 ( -18.02 - 84.64 ) | 64.99 ( -11.8 - 162.08 ) | 11.45 ( -2.1 - 28.55 ) | -4.86(-5.18 to -4.54) |
| Bolivia (Plurinational State of) | 1596.99 ( -444.45 - 3204.71 ) | 57.74 ( -16.34 - 117.97 ) | 1714.95 ( -304.34 - 4323.79 ) | 20.94 ( -3.77 - 53 ) | -3.24(-3.4 to -3.07) |
| Bosnia and Herzegovina | 400.33 ( -164.35 - 752.41 ) | 11.37 ( -4.69 - 21.37 ) | 420.49 ( -102.73 - 919.09 ) | 6.74 ( -1.66 - 14.75 ) | -1.95(-2.06 to -1.85) |
| Botswana | 230.76 ( -84.04 - 440.68 ) | 47.99 ( -18.15 - 92.25 ) | 317.64 ( -72.88 - 682.73 ) | 24.68 ( -5.84 - 53.27 ) | -2.17(-2.23 to -2.12) |
| Brazil | 22000.8 ( -5301.55 - 47466.64 ) | 28.61 ( -7.01 - 62 ) | 15693.55 ( -2533.57 - 43928.67 ) | 6.46 ( -1.05 - 18.1 ) | -4.49(-4.62 to -4.37) |
| Brunei Darussalam | 4.66 ( -0.7 - 12.1 ) | 4.78 ( -0.77 - 12.12 ) | 2.08 ( -0.23 - 7.48 ) | 0.71 ( -0.08 - 2.6 ) | -6.43(-6.72 to -6.14) |
| Bulgaria | 312.52 ( -48.77 - 790.16 ) | 2.9 ( -0.46 - 7.24 ) | 366.18 ( -63.11 - 942.47 ) | 2.49 ( -0.43 - 6.42 ) | -0.09(-0.33 to 0.15) |
| Burkina Faso | 1346.88 ( -627.3 - 2470.97 ) | 38.71 ( -18.37 - 70.74 ) | 4548.76 ( -2019.68 - 8277.63 ) | 54.63 ( -24.58 - 100.27 ) | 0.34(-0.06 to 0.74) |
| Burundi | 431.61 ( -198.33 - 778.24 ) | 20.33 ( -9.63 - 36.63 ) | 711.12 ( -330.62 - 1290.1 ) | 16.82 ( -8.08 - 30.66 ) | -1.09(-1.25 to -0.93) |
| Cabo Verde | 140.81 ( -51.58 - 267.76 ) | 59.7 ( -21.8 - 113.58 ) | 81.29 ( -14.29 - 199.65 ) | 19.22 ( -3.39 - 46.98 ) | -3.94(-4.11 to -3.78) |
| Cambodia | 7089.71 ( -3268.1 - 12978.2 ) | 180.95 ( -85.64 - 331.88 ) | 10463.16 ( -4247.1 - 19962.42 ) | 98.25 ( -40.33 - 186.55 ) | -2.3(-2.43 to -2.18) |
| Cameroon | 1607.81 ( -664.67 - 2972.2 ) | 43.89 ( -18.38 - 80.88 ) | 3639.68 ( -1309.28 - 6850.96 ) | 33.92 ( -12.37 - 64.48 ) | -0.91(-0.99 to -0.83) |
| Canada | 110.13 ( -12.27 - 379.28 ) | 0.34 ( -0.04 - 1.18 ) | 28.28 ( -3.01 - 103.09 ) | 0.04 ( 0 - 0.14 ) | -9(-9.79 to -8.2) |
| Central African Republic | 141.03 ( -63.41 - 266.09 ) | 19.54 ( -9.4 - 35.76 ) | 345.25 ( -159.04 - 649.5 ) | 20.9 ( -10.06 - 38.16 ) | 0.34(0.28 to 0.4) |
| Chad | 1609.38 ( -732.91 - 2932.4 ) | 61.63 ( -28.46 - 113.41 ) | 2982.96 ( -1370.96 - 5469.99 ) | 59.07 ( -27.34 - 109.08 ) | -0.01(-0.09 to 0.07) |
| Chile | 1322.44 ( -254.01 - 3174.51 ) | 14 ( -2.71 - 33.69 ) | 933.88 ( -114.43 - 2998.57 ) | 3.62 ( -0.44 - 11.66 ) | -4.63(-4.74 to -4.53) |
| China | 227938.84 ( -94383.43 - 420551.64 ) | 33.53 ( -14.22 - 61.85 ) | 338546.8 ( -77701.29 - 753535.32 ) | 16.98 ( -3.92 - 37.98 ) | -1.96(-2.45 to -1.47) |
| Colombia | 4108.68 ( -932.57 - 9319.86 ) | 26.36 ( -6.05 - 59.31 ) | 4001.52 ( -534.11 - 12387.46 ) | 7.17 ( -0.96 - 22.19 ) | -4.09(-4.33 to -3.85) |
| Comoros | 109.69 ( -50.29 - 200.5 ) | 62.65 ( -29.55 - 113.71 ) | 183.16 ( -71.63 - 340.1 ) | 40.8 ( -16.14 - 75.79 ) | -1.41(-1.45 to -1.37) |
| Congo | 273.55 ( -123.9 - 506.79 ) | 30.33 ( -14.11 - 55.44 ) | 378.51 ( -112.36 - 782.36 ) | 16.74 ( -5.15 - 34.02 ) | -1.93(-2.08 to -1.78) |
| Cook Islands | 3.92 ( -0.58 - 10.06 ) | 34.35 ( -5.2 - 88.25 ) | 3.42 ( -0.44 - 10.05 ) | 13.24 ( -1.71 - 38.91 ) | -2.88(-3.22 to -2.53) |
| Costa Rica | 398.53 ( -79.26 - 928.04 ) | 23.88 ( -4.77 - 55.66 ) | 339.02 ( -45.81 - 1077.74 ) | 6.17 ( -0.84 - 19.59 ) | -4.35(-4.45 to -4.26) |
| Cote d'Ivoire | 856.82 ( -372.04 - 1573.56 ) | 30.07 ( -13.49 - 55.85 ) | 4928.67 ( -1918.26 - 9105.68 ) | 50.88 ( -20.14 - 95.93 ) | -3.54(-3.59 to -3.5) |
| Croatia | 244.46 ( -38.99 - 623.84 ) | 4.41 ( -0.7 - 11.44 ) | 144.28 ( -18.03 - 461.18 ) | 1.54 ( -0.19 - 4.91 ) | -4.71(-4.82 to -4.59) |
| Cuba | 603.63 ( -73.97 - 1904.68 ) | 5.96 ( -0.73 - 18.8 ) | 267.62 ( -26.04 - 912.55 ) | 1.33 ( -0.13 - 4.56 ) | -6.83(-7.15 to -6.5) |
| Cyprus | 3.68 ( -0.42 - 12.53 ) | 0.52 ( -0.06 - 1.8 ) | 1.34 ( -0.13 - 4.83 ) | 0.07 ( -0.01 - 0.24 ) | -3(-3.06 to -2.94) |
| Czechia | 149.23 ( -14.42 - 474.59 ) | 1.1 ( -0.11 - 3.5 ) | 101.98 ( -12.03 - 343.56 ) | 0.45 ( -0.05 - 1.52 ) | 0.48(-0.07 to 1.03) |
| Democratic People's Republic of Korea | 1516.41 ( -775.4 - 2915.65 ) | 12.66 ( -6.63 - 23.79 ) | 2723.14 ( -1157.6 - 5424.56 ) | 9.18 ( -3.88 - 17.94 ) | -1.07(-1.12 to -1.03) |
| Democratic Republic of the Congo | 979.56 ( -470.31 - 1807.58 ) | 8.35 ( -4.16 - 15.62 ) | 2525.3 ( -1182.72 - 4613.5 ) | 9.04 ( -4.4 - 16.76 ) | 0.41(0.26 to 0.56) |
| Denmark | 11.73 ( -1.33 - 42.77 ) | 0.14 ( -0.02 - 0.51 ) | 5.63 ( -0.55 - 21.11 ) | 0.05 ( 0 - 0.17 ) | -3.7(-3.77 to -3.62) |
| Djibouti | 59.9 ( -15.3 - 120.23 ) | 49.61 ( -12.71 - 100.4 ) | 107.08 ( -16.57 - 272.61 ) | 19.25 ( -3.11 - 47.78 ) | -3.19(-3.54 to -2.83) |
| Dominica | 10.84 ( -3.04 - 22.69 ) | 18.42 ( -5.16 - 38.71 ) | 3.04 ( -0.42 - 8.68 ) | 3.88 ( -0.54 - 11.06 ) | -5.12(-5.31 to -4.93) |
| Dominican Republic | 701.17 ( -204.02 - 1454.01 ) | 21.1 ( -6.19 - 43.41 ) | 471.83 ( -64.47 - 1376.97 ) | 4.85 ( -0.67 - 14.19 ) | -4.76(-4.98 to -4.54) |
| Ecuador | 1222.24 ( -262.29 - 2819.11 ) | 25.07 ( -5.44 - 58 ) | 872.28 ( -96.9 - 2719.57 ) | 5.47 ( -0.61 - 17.08 ) | -4.96(-5.02 to -4.9) |
| Egypt | 3186.48 ( -399.77 - 9383.93 ) | 14.44 ( -1.91 - 43.57 ) | 163.09 ( -16.76 - 620.13 ) | 0.31 ( -0.03 - 1.17 ) | -13.32(-13.85 to -12.78) |
| El Salvador | 1225.05 ( -439.56 - 2385.23 ) | 43.07 ( -15.56 - 84.02 ) | 652.24 ( -107.98 - 1746.24 ) | 9.96 ( -1.65 - 26.77 ) | -4.68(-4.93 to -4.43) |
| Equatorial Guinea | 280.92 ( -109.29 - 523.92 ) | 160.92 ( -64.04 - 297.29 ) | 140.74 ( -24.13 - 340.48 ) | 28.94 ( -5.1 - 70.56 ) | -5.98(-6.22 to -5.75) |
| Eritrea | 652.71 ( -261.44 - 1222.89 ) | 69.91 ( -29.15 - 127.61 ) | 1041.21 ( -365.97 - 1995.71 ) | 43.91 ( -15.75 - 82.46 ) | -1.35(-1.43 to -1.26) |
| Estonia | 112.92 ( -20.02 - 284.32 ) | 5.61 ( -0.99 - 14.17 ) | 93.13 ( -12.08 - 261.52 ) | 3.08 ( -0.39 - 8.57 ) | -2.08(-2.21 to -1.95) |
| Eswatini | 156.95 ( -59.13 - 294.52 ) | 61.66 ( -23.5 - 115.12 ) | 174.01 ( -53.95 - 358.11 ) | 36.23 ( -11.5 - 74.44 ) | -1.7(-1.82 to -1.57) |
| Ethiopia | 24429.23 ( -10513.25 - 44897.74 ) | 131.16 ( -57.69 - 236.79 ) | 51327.33 ( -22359.91 - 93869.92 ) | 116.12 ( -51.2 - 210.53 ) | -0.27(-0.32 to -0.22) |
| Fiji | 222.77 ( -69.63 - 432.46 ) | 72.79 ( -23.53 - 142.33 ) | 239.02 ( -46.06 - 549.42 ) | 36.52 ( -7.12 - 84.24 ) | -1.98(-2.16 to -1.79) |
| Finland | 12.34 ( -1.24 - 44.01 ) | 0.18 ( -0.02 - 0.62 ) | 6.93 ( -0.63 - 27.13 ) | 0.05 ( 0 - 0.2 ) | -4.09(-4.28 to -3.9) |
| France | 317.24 ( -30.32 - 1174.17 ) | 0.37 ( -0.04 - 1.36 ) | 116.4 ( -12.05 - 441.55 ) | 0.08 ( -0.01 - 0.29 ) | -5.38(-5.61 to -5.15) |
| Gabon | 125.83 ( -34.77 - 255.68 ) | 24.3 ( -6.79 - 49.16 ) | 47.4 ( -5.95 - 135.23 ) | 5.26 ( -0.67 - 15.09 ) | -5.1(-5.35 to -4.84) |
| Gambia | 313.74 ( -145.01 - 569.31 ) | 96.98 ( -45.66 - 176.52 ) | 673.99 ( -305.36 - 1221.94 ) | 75.53 ( -35 - 136.61 ) | -0.74(-0.8 to -0.68) |
| Georgia | 1468.27 ( -387.49 - 3189.72 ) | 24.78 ( -6.54 - 53.76 ) | 911.57 ( -181.89 - 2262.14 ) | 14.59 ( -2.9 - 36.35 ) | -1.82(-2.25 to -1.4) |
| Germany | 105.9 ( -10.7 - 384.85 ) | 0.08 ( -0.01 - 0.3 ) | 72.33 ( -7.66 - 271.32 ) | 0.03 ( 0 - 0.13 ) | -3(-3.12 to -2.88) |
| Ghana | 2001.41 ( -948.12 - 3703.22 ) | 40.54 ( -19.64 - 75.7 ) | 6044.6 ( -2316.92 - 11722.62 ) | 41.35 ( -15.95 - 80.21 ) | -0.31(-0.59 to -0.03) |
| Greece | 224.8 ( -24.19 - 731.44 ) | 1.54 ( -0.17 - 4.99 ) | 63.8 ( -7.35 - 236.94 ) | 0.23 ( -0.03 - 0.86 ) | -6.21(-6.48 to -5.95) |
| Greenland | 0.08 ( -0.01 - 0.3 ) | 0.29 ( -0.03 - 1.12 ) | 0.05 ( -0.01 - 0.19 ) | 0.09 ( -0.01 - 0.34 ) | -4.19(-4.42 to -3.96) |
| Grenada | 11.88 ( -2.38 - 27.87 ) | 15.07 ( -2.97 - 35.46 ) | 1.37 ( -0.17 - 4.59 ) | 1.37 ( -0.17 - 4.67 ) | -7.69(-8.16 to -7.22) |
| Guam | 8.99 ( -1.21 - 27.52 ) | 13.97 ( -1.87 - 43.51 ) | 25.54 ( -3.2 - 77.96 ) | 11.86 ( -1.5 - 36.1 ) | -0.05(-0.24 to 0.13) |
| Guatemala | 1354.74 ( -486.35 - 2572.47 ) | 48.34 ( -17.82 - 92.44 ) | 3012.44 ( -962.11 - 6042.18 ) | 29.27 ( -9.4 - 58.64 ) | -1.39(-1.48 to -1.31) |
| Guinea | 1943.31 ( -926.95 - 3576.6 ) | 64.52 ( -31.23 - 118.32 ) | 3152.2 ( -1488.71 - 5751.1 ) | 61.01 ( -29.53 - 112.01 ) | -0.25(-0.39 to -0.12) |
| Guinea-Bissau | 131.96 ( -62.82 - 245.69 ) | 41.13 ( -20.1 - 76.31 ) | 233.97 ( -112.73 - 420.07 ) | 39.6 ( -19.77 - 73.37 ) | -0.16(-0.35 to 0.03) |
| Guyana | 44.92 ( -7.07 - 112.91 ) | 13.34 ( -2.1 - 33.67 ) | 18.66 ( -2.45 - 58.33 ) | 3.41 ( -0.44 - 10.75 ) | -4.39(-4.65 to -4.13) |
| Haiti | 1099.37 ( -517.46 - 2022.91 ) | 41.58 ( -20.2 - 75.22 ) | 1667.85 ( -746.83 - 3028.55 ) | 27.66 ( -12.98 - 50.26 ) | -1.34(-1.38 to -1.31) |
| Honduras | 532.37 ( -196.84 - 1000.01 ) | 30.1 ( -11.33 - 57.01 ) | 866.63 ( -253.92 - 1797.07 ) | 15.82 ( -4.7 - 32.82 ) | -2.1(-2.15 to -2.04) |
| Hungary | 1613.32 ( -385.88 - 3684.73 ) | 11.31 ( -2.72 - 26 ) | 1375.37 ( -223.43 - 3494.64 ) | 6.76 ( -1.08 - 17.09 ) | -1.6(-1.65 to -1.56) |
| Iceland | 0.43 ( -0.04 - 1.45 ) | 0.15 ( -0.01 - 0.5 ) | 0.31 ( -0.04 - 1.12 ) | 0.05 ( -0.01 - 0.18 ) | -3.38(-3.61 to -3.14) |
| India | 558412.89 ( -231916.58 - 1010658.49 ) | 140.94 ( -59.64 - 255.77 ) | 783329.99 ( -267646.86 - 1551569.17 ) | 71.11 ( -24.37 - 142.36 ) | -2.09(-2.28 to -1.91) |
| Indonesia | 89199.06 ( -31016.58 - 169853.79 ) | 106.21 ( -37.76 - 202.76 ) | 88433.27 ( -18694.53 - 208675.32 ) | 43.15 ( -9.28 - 102.44 ) | -2.96(-3.26 to -2.65) |
| Iran (Islamic Republic of) | 1886.99 ( -297.06 - 5205.08 ) | 9.05 ( -1.47 - 24.68 ) | 230.58 ( -21.36 - 843.7 ) | 0.32 ( -0.03 - 1.19 ) | -11.1(-11.31 to -10.9) |
| Iraq | 1590.18 ( -212.03 - 4979.82 ) | 21.57 ( -2.88 - 67.49 ) | 285.1 ( -30.5 - 1026.02 ) | 1.41 ( -0.15 - 5.03 ) | -8.86(-9.63 to -8.08) |
| Ireland | 14.71 ( -1.36 - 50.47 ) | 0.38 ( -0.04 - 1.27 ) | 5.8 ( -0.53 - 20.79 ) | 0.07 ( -0.01 - 0.26 ) | -5.73(-5.99 to -5.46) |
| Israel | 12.34 ( -1.13 - 46.3 ) | 0.27 ( -0.02 - 1 ) | 11.09 ( -1.06 - 42.69 ) | 0.09 ( -0.01 - 0.33 ) | -3.9(-4.04 to -3.77) |
| Italy | 1430.85 ( -144.22 - 4615.53 ) | 1.65 ( -0.17 - 5.34 ) | 441.98 ( -53.08 - 1622.16 ) | 0.29 ( -0.03 - 1.05 ) | -5.75(-6.01 to -5.49) |
| Jamaica | 345.17 ( -102.56 - 708.02 ) | 18.7 ( -5.51 - 38.54 ) | 166.21 ( -28.71 - 470.19 ) | 5.16 ( -0.89 - 14.55 ) | -3.8(-4.3 to -3.3) |
| Japan | 727.17 ( -78.17 - 2661.66 ) | 0.45 ( -0.05 - 1.65 ) | 373.88 ( -33.43 - 1356.94 ) | 0.09 ( -0.01 - 0.33 ) | -5.32(-5.67 to -4.98) |
| Jordan | 7.71 ( -0.78 - 26.65 ) | 0.73 ( -0.07 - 2.53 ) | 3.57 ( -0.34 - 12.33 ) | 0.06 ( -0.01 - 0.2 ) | -7.86(-8.09 to -7.63) |
| Kazakhstan | 2552.73 ( -580.24 - 5745.15 ) | 22.29 ( -5.11 - 50.38 ) | 1159.55 ( -147.02 - 3448.92 ) | 7.31 ( -0.92 - 21.74 ) | -4.12(-4.56 to -3.68) |
| Kenya | 7795.92 ( -3459.2 - 14144.33 ) | 97.59 ( -44.04 - 177.23 ) | 13299.58 ( -5661.39 - 24122.85 ) | 63.37 ( -27.69 - 115.71 ) | -1.25(-1.39 to -1.12) |
| Kiribati | 24.39 ( -10.4 - 44.46 ) | 78.02 ( -34.19 - 141.71 ) | 26.62 ( -6.57 - 55.88 ) | 45.17 ( -11.43 - 93.85 ) | -1.76(-1.93 to -1.59) |
| Kuwait | 23.36 ( -2.24 - 79.58 ) | 4.7 ( -0.46 - 15.55 ) | 8.77 ( -0.91 - 31.63 ) | 0.35 ( -0.04 - 1.24 ) | -8.43(-8.53 to -8.33) |
| Kyrgyzstan | 763.67 ( -202.35 - 1598.79 ) | 27.64 ( -7.35 - 58.01 ) | 685.88 ( -124.14 - 1586.82 ) | 16.36 ( -3.02 - 38.1 ) | -1.74(-2.02 to -1.46) |
| Lao People's Democratic Republic | 447.92 ( -233.12 - 818.64 ) | 27.97 ( -14.76 - 50.91 ) | 801.56 ( -390.34 - 1483.53 ) | 21.47 ( -10.62 - 40.68 ) | -0.81(-0.85 to -0.77) |
| Latvia | 374.23 ( -85.1 - 852.7 ) | 10.52 ( -2.38 - 23.95 ) | 244.56 ( -37.89 - 627.31 ) | 5.55 ( -0.85 - 14.29 ) | -2.28(-2.43 to -2.12) |
| Lebanon | 236.53 ( -27.6 - 782.29 ) | 12.24 ( -1.45 - 40.13 ) | 39.6 ( -4.01 - 145.13 ) | 0.63 ( -0.06 - 2.32 ) | -9.86(-10.28 to -9.43) |
| Lesotho | 497.34 ( -161.26 - 959.34 ) | 63.84 ( -20.9 - 123.66 ) | 392.36 ( -109.7 - 777.5 ) | 41.65 ( -12.05 - 83.37 ) | -1.32(-1.43 to -1.21) |
| Liberia | 728.64 ( -347.91 - 1312.61 ) | 69.26 ( -33.49 - 124.35 ) | 906.32 ( -432.91 - 1643.34 ) | 49.68 ( -23.84 - 90.68 ) | -1.17(-1.23 to -1.12) |
| Libya | 405.65 ( -47.21 - 1163.11 ) | 23.61 ( -2.81 - 67.7 ) | 42.5 ( -3.93 - 163.93 ) | 0.94 ( -0.09 - 3.54 ) | -10.21(-10.55 to -9.88) |
| Lithuania | 233.09 ( -31.98 - 690 ) | 5.18 ( -0.71 - 15.25 ) | 111.88 ( -13.57 - 374.3 ) | 1.75 ( -0.21 - 5.93 ) | -3.74(-3.84 to -3.64) |
| Luxembourg | 1.91 ( -0.21 - 7.26 ) | 0.36 ( -0.04 - 1.35 ) | 0.81 ( -0.08 - 3 ) | 0.07 ( -0.01 - 0.27 ) | -5.44(-5.57 to -5.31) |
| Madagascar | 1453.1 ( -673.88 - 2581.49 ) | 32.16 ( -15.27 - 57.44 ) | 2265.21 ( -1067.02 - 4032.21 ) | 24.48 ( -11.98 - 44.32 ) | -0.88(-0.93 to -0.82) |
| Malawi | 2291.57 ( -1027.27 - 4179.89 ) | 64.56 ( -30.37 - 116.07 ) | 2789.5 ( -1282.9 - 5031.73 ) | 41.75 ( -20.15 - 75.71 ) | -1.36(-1.4 to -1.32) |
| Malaysia | 1399.85 ( -151.18 - 4614.14 ) | 16.28 ( -1.77 - 54.31 ) | 688.15 ( -57.85 - 2384.5 ) | 2.64 ( -0.22 - 9.17 ) | -5.6(-6.05 to -5.15) |
| Maldives | 22.74 ( -8.7 - 43.12 ) | 33.71 ( -13.58 - 63.63 ) | 17.32 ( -3.18 - 50 ) | 5.84 ( -1.03 - 16.81 ) | -6.13(-6.29 to -5.97) |
| Mali | 3320.74 ( -1523.91 - 6163.07 ) | 94.17 ( -43.75 - 173.51 ) | 6917.12 ( -3294.77 - 12688.27 ) | 85.94 ( -41.74 - 155.04 ) | -0.53(-0.71 to -0.35) |
| Malta | 3.87 ( -0.46 - 13.65 ) | 0.96 ( -0.11 - 3.41 ) | 1.39 ( -0.15 - 5.32 ) | 0.14 ( -0.01 - 0.52 ) | -6.74(-6.96 to -6.53) |
| Marshall Islands | 7.04 ( -1.43 - 15.68 ) | 49.53 ( -10.33 - 108.63 ) | 8.12 ( -1.61 - 18.62 ) | 29.81 ( -6.01 - 67.45 ) | -1.31(-1.48 to -1.14) |
| Mauritania | 587.83 ( -234.06 - 1078.06 ) | 63.85 ( -25.74 - 116.7 ) | 776.41 ( -224.21 - 1572.21 ) | 39.14 ( -11.45 - 79.39 ) | -1.6(-1.66 to -1.53) |
| Mauritius | 90.7 ( -12.43 - 275.1 ) | 14.55 ( -2.02 - 44.65 ) | 39.53 ( -4.35 - 138.7 ) | 2.27 ( -0.25 - 7.97 ) | -6.02(-6.12 to -5.92) |
| Mexico | 9226.21 ( -2046.94 - 20528.14 ) | 24.42 ( -5.5 - 54.34 ) | 11850.79 ( -2013.06 - 31975.47 ) | 10.04 ( -1.71 - 26.8 ) | -2.98(-3.07 to -2.89) |
| Micronesia (Federated States of) | 29.15 ( -10.02 - 55.92 ) | 66.51 ( -23.25 - 127.48 ) | 19.24 ( -3.53 - 45.62 ) | 31.87 ( -5.94 - 74.83 ) | -2.41(-2.48 to -2.33) |
| Monaco | 0.08 ( -0.01 - 0.28 ) | 0.11 ( -0.01 - 0.37 ) | 0.04 ( 0 - 0.13 ) | 0.03 ( 0 - 0.12 ) | -3.84(-3.99 to -3.69) |
| Mongolia | 271.98 ( -103.29 - 522.29 ) | 29.03 ( -11.2 - 55.99 ) | 328.35 ( -95.4 - 694.41 ) | 17.09 ( -5.1 - 35.65 ) | -1.79(-1.85 to -1.73) |
| Montenegro | 51.08 ( -16.94 - 101.22 ) | 8.66 ( -2.86 - 17.33 ) | 60.93 ( -16.09 - 131.54 ) | 6.58 ( -1.74 - 14.21 ) | -0.85(-0.98 to -0.72) |
| Morocco | 3871.47 ( -580.09 - 10040.59 ) | 29.35 ( -4.43 - 76.13 ) | 1391.24 ( -147.31 - 4648.76 ) | 4.41 ( -0.47 - 14.72 ) | -6.31(-6.75 to -5.87) |
| Mozambique | 3541.14 ( -1516.09 - 6501.06 ) | 65.32 ( -29.24 - 119.35 ) | 4928.3 ( -2141.15 - 9000.75 ) | 50.41 ( -23.05 - 90.87 ) | -0.62(-0.73 to -0.51) |
| Myanmar | 31929.97 ( -14606.78 - 58066.57 ) | 159.52 ( -74.06 - 290 ) | 42269.67 ( -17224.21 - 78856.65 ) | 96.65 ( -39.77 - 180.59 ) | -1.9(-2.09 to -1.71) |
| Namibia | 321.65 ( -123.14 - 607.26 ) | 57.71 ( -22.97 - 107.31 ) | 393.34 ( -113.9 - 795.41 ) | 32.62 ( -9.63 - 66.43 ) | -1.85(-1.96 to -1.73) |
| Nauru | 1.06 ( -0.17 - 2.78 ) | 27.57 ( -4.53 - 71.9 ) | 0.38 ( -0.04 - 1.18 ) | 7.91 ( -0.88 - 24.8 ) | -4.17(-4.4 to -3.95) |
| Nepal | 4998.47 ( -2070.62 - 9393.98 ) | 65.43 ( -27.98 - 121.52 ) | 9583.47 ( -3328.46 - 18448.33 ) | 44.71 ( -15.67 - 85.43 ) | -1.48(-1.55 to -1.41) |
| Netherlands | 37.43 ( -3.82 - 131.74 ) | 0.19 ( -0.02 - 0.66 ) | 13.43 ( -1.42 - 50.87 ) | 0.04 ( 0 - 0.14 ) | -5.4(-5.61 to -5.19) |
| New Zealand | 20.05 ( -1.97 - 75.88 ) | 0.53 ( -0.05 - 2.01 ) | 9.94 ( -0.91 - 36.27 ) | 0.12 ( -0.01 - 0.42 ) | -5.01(-5.26 to -4.75) |
| Nicaragua | 682.85 ( -281.91 - 1291.07 ) | 49.69 ( -20.7 - 93.27 ) | 1103.66 ( -297.43 - 2340.17 ) | 24.35 ( -6.62 - 51.58 ) | -2.32(-2.35 to -2.29) |
| Niger | 1655.01 ( -718.76 - 3053.21 ) | 68.61 ( -31.44 - 125.49 ) | 5267.19 ( -2462.59 - 9596.58 ) | 72 ( -34.28 - 131.52 ) | 0.09(0.05 to 0.13) |
| Nigeria | 51036.05 ( -22756.07 - 93091.21 ) | 123.75 ( -55.76 - 224.94 ) | 79485.3 ( -28083.68 - 151623.09 ) | 92.54 ( -32.92 - 177.63 ) | -0.98(-1.1 to -0.86) |
| Niue | 0.91 ( -0.19 - 2.09 ) | 38.93 ( -8.35 - 89.7 ) | 0.18 ( -0.02 - 0.58 ) | 8.81 ( -1.08 - 27.59 ) | -5.14(-5.31 to -4.97) |
| North Macedonia | 154.27 ( -43.33 - 307.16 ) | 9.12 ( -2.57 - 18.15 ) | 134.82 ( -27.2 - 334 ) | 4.54 ( -0.92 - 11.35 ) | -2.38(-2.55 to -2.22) |
| Northern Mariana Islands | 3.36 ( -0.54 - 8.44 ) | 24.33 ( -3.94 - 60.59 ) | 5.73 ( -0.86 - 16.81 ) | 13.03 ( -1.97 - 38.84 ) | -1.42(-1.7 to -1.13) |
| Norway | 7.57 ( -0.71 - 27.31 ) | 0.11 ( -0.01 - 0.38 ) | 3.43 ( -0.35 - 12.47 ) | 0.03 ( 0 - 0.12 ) | -3.9(-4.05 to -3.75) |
| Oman | 333.43 ( -56.6 - 820.21 ) | 52.84 ( -9.01 - 128.86 ) | 30.8 ( -2.7 - 108.38 ) | 1.71 ( -0.15 - 6.18 ) | -11.32(-11.5 to -11.14) |
| Pakistan | 89352.15 ( -36317.88 - 163888.02 ) | 172.35 ( -70.63 - 316.21 ) | 131542.23 ( -37351.26 - 268751.65 ) | 122.45 ( -35.33 - 247.37 ) | -1.38(-1.6 to -1.15) |
| Palau | 0.03 ( 0 - 0.1 ) | 0.29 ( -0.03 - 1.16 ) | 0.03 ( 0 - 0.1 ) | 0.13 ( -0.01 - 0.52 ) | -1.7(-2.02 to -1.37) |
| Palestine | 167.99 ( -21.42 - 502.32 ) | 21.26 ( -2.74 - 63.49 ) | 50.11 ( -4.95 - 173.77 ) | 2.31 ( -0.23 - 7.93 ) | -6.91(-7.09 to -6.73) |
| Panama | 591.87 ( -129.47 - 1323.33 ) | 41.9 ( -9.28 - 93.81 ) | 567.93 ( -83.48 - 1697.55 ) | 12.68 ( -1.86 - 37.88 ) | -3.95(-4.27 to -3.62) |
| Papua New Guinea | 1785.73 ( -789.3 - 3246.55 ) | 117.84 ( -53.53 - 214.34 ) | 4132.67 ( -1745.45 - 7641.46 ) | 95.29 ( -41.05 - 176.95 ) | -0.71(-0.84 to -0.58) |
| Paraguay | 1128.11 ( -349.8 - 2212.91 ) | 54.03 ( -16.9 - 105.46 ) | 1131.22 ( -255.75 - 2534.52 ) | 20.45 ( -4.69 - 46 ) | -3.4(-3.57 to -3.22) |
| Peru | 8356.81 ( -2816.58 - 16225.24 ) | 75.24 ( -25.46 - 146.74 ) | 7678.65 ( -1413.81 - 19288.83 ) | 23.11 ( -4.26 - 57.86 ) | -3.94(-4.32 to -3.57) |
| Philippines | 17362.86 ( -6673.49 - 33012.55 ) | 67.72 ( -26.52 - 129.02 ) | 31855.11 ( -9114.65 - 66530.8 ) | 45.19 ( -13.18 - 93.92 ) | -1.19(-1.29 to -1.1) |
| Poland | 2695.41 ( -532.17 - 6242.48 ) | 6.47 ( -1.28 - 14.91 ) | 2540.26 ( -407.02 - 6715.45 ) | 3.45 ( -0.55 - 9.03 ) | -2.07(-2.15 to -1.98) |
| Portugal | 263.08 ( -28.77 - 835.38 ) | 2.04 ( -0.22 - 6.44 ) | 68.18 ( -6.69 - 257.34 ) | 0.25 ( -0.02 - 0.94 ) | -6.99(-7.15 to -6.83) |
| Puerto Rico | 3.72 ( -0.36 - 14.83 ) | 0.1 ( -0.01 - 0.42 ) | 2.98 ( -0.34 - 10.67 ) | 0.04 ( 0 - 0.13 ) | -3.11(-3.26 to -2.96) |
| Qatar | 1.21 ( -0.13 - 4.77 ) | 1.32 ( -0.15 - 5.14 ) | 0.48 ( -0.05 - 1.7 ) | 0.06 ( -0.01 - 0.21 ) | -10.02(-10.22 to -9.81) |
| Republic of Korea | 287.61 ( -28.84 - 1070.92 ) | 1.09 ( -0.11 - 3.89 ) | 58.45 ( -5.26 - 214.61 ) | 0.06 ( -0.01 - 0.24 ) | -9.06(-9.68 to -8.45) |
| Republic of Moldova | 1077.6 ( -301.73 - 2210.89 ) | 27.63 ( -7.8 - 56.39 ) | 358.05 ( -50.4 - 1085.59 ) | 6.01 ( -0.85 - 18.29 ) | -6.01(-6.48 to -5.53) |
| Romania | 1983.61 ( -442.41 - 4281.57 ) | 7.83 ( -1.77 - 16.89 ) | 1344.38 ( -217.96 - 3778.15 ) | 3.43 ( -0.55 - 9.61 ) | -3.02(-3.15 to -2.88) |
| Russian Federation | 4527.15 ( -770.81 - 12936.02 ) | 2.72 ( -0.46 - 7.77 ) | 2115.82 ( -263.74 - 6948.36 ) | 0.89 ( -0.11 - 2.9 ) | -4.19(-4.46 to -3.92) |
| Rwanda | 631.87 ( -289.22 - 1143.71 ) | 25.66 ( -12.19 - 46.16 ) | 903.16 ( -441.44 - 1625.68 ) | 16.4 ( -8.29 - 29.84 ) | -1.63(-1.72 to -1.54) |
| Saint Kitts and Nevis | 2.48 ( -0.32 - 7.26 ) | 6.68 ( -0.85 - 19.66 ) | 0.73 ( -0.08 - 2.52 ) | 1.26 ( -0.14 - 4.37 ) | -5.51(-5.58 to -5.44) |
| Saint Lucia | 12.77 ( -2.86 - 29.02 ) | 15.52 ( -3.49 - 35.21 ) | 5.52 ( -0.65 - 17.54 ) | 2.36 ( -0.28 - 7.49 ) | -6.25(-6.35 to -6.16) |
| Saint Vincent and the Grenadines | 9.49 ( -1.89 - 22.57 ) | 13.88 ( -2.75 - 33.02 ) | 2.71 ( -0.3 - 9.41 ) | 1.99 ( -0.22 - 6.89 ) | -6.36(-6.55 to -6.16) |
| Samoa | 48.65 ( -19.59 - 89.99 ) | 64.46 ( -26.37 - 119.81 ) | 57.77 ( -17.92 - 113.64 ) | 44.34 ( -13.87 - 87.64 ) | -1.08(-1.15 to -1.01) |
| San Marino | 0.08 ( -0.01 - 0.29 ) | 0.23 ( -0.03 - 0.82 ) | 0.05 ( 0 - 0.18 ) | 0.06 ( -0.01 - 0.21 ) | -4.48(-4.68 to -4.28) |
| Sao Tome and Principe | 43.3 ( -19.68 - 78.41 ) | 71.62 ( -32.8 - 130.29 ) | 34.95 ( -8.58 - 74.07 ) | 35.24 ( -8.74 - 74.67 ) | -2.46(-2.67 to -2.25) |
| Saudi Arabia | 3369.02 ( -426.8 - 9261.35 ) | 65.65 ( -8.32 - 179.09 ) | 234.37 ( -21.2 - 880.04 ) | 1.52 ( -0.14 - 5.7 ) | -12.29(-12.49 to -12.1) |
| Senegal | 1488.47 ( -608.38 - 2743.4 ) | 50.76 ( -21.39 - 93.52 ) | 2496.16 ( -977.71 - 4668.12 ) | 35.7 ( -14.1 - 66.37 ) | -0.8(-0.88 to -0.72) |
| Serbia | 940.91 ( -276.81 - 1866.78 ) | 9.71 ( -2.92 - 19.53 ) | 842.5 ( -176.04 - 1997.21 ) | 4.99 ( -1.03 - 11.87 ) | -2.34(-2.41 to -2.27) |
| Seychelles | 6.07 ( -0.7 - 20.31 ) | 10.75 ( -1.24 - 35.94 ) | 1.17 ( -0.11 - 4.45 ) | 1.12 ( -0.11 - 4.27 ) | -7.4(-7.62 to -7.19) |
| Sierra Leone | 723.8 ( -346.32 - 1346.83 ) | 39.65 ( -19 - 74.12 ) | 1010.21 ( -489.13 - 1848.72 ) | 30.63 ( -15.23 - 56.57 ) | -0.85(-1.01 to -0.69) |
| Singapore | 75.28 ( -10.63 - 225.84 ) | 3.71 ( -0.52 - 10.96 ) | 17.82 ( -1.76 - 62.83 ) | 0.21 ( -0.02 - 0.75 ) | -9.47(-9.93 to -9.01) |
| Slovakia | 56.19 ( -6.32 - 187.5 ) | 0.97 ( -0.11 - 3.22 ) | 34.78 ( -3.72 - 123.34 ) | 0.37 ( -0.04 - 1.3 ) | -3.34(-3.41 to -3.26) |
| Slovenia | 118.89 ( -20.36 - 288.31 ) | 4.9 ( -0.83 - 11.83 ) | 105.54 ( -12.98 - 317.7 ) | 2.21 ( -0.27 - 6.62 ) | -2.63(-2.7 to -2.56) |
| Solomon Islands | 97.75 ( -44.94 - 177.87 ) | 85.41 ( -40.92 - 154.56 ) | 190.45 ( -85.75 - 345.18 ) | 64.59 ( -29.8 - 118.85 ) | -0.85(-0.89 to -0.81) |
| Somalia | 1184.46 ( -495.69 - 2214.06 ) | 52.77 ( -23.26 - 96.71 ) | 2630.91 ( -1067.4 - 4881.05 ) | 47.08 ( -20.52 - 85.03 ) | -0.41(-0.46 to -0.36) |
| South Africa | 8149.84 ( -2325.84 - 17290.62 ) | 41.11 ( -11.88 - 86.42 ) | 5351.3 ( -881.48 - 13283.02 ) | 12.87 ( -2.18 - 31.96 ) | -4.31(-4.51 to -4.11) |
| South Sudan | 3349.71 ( -1549.05 - 6024.54 ) | 145.28 ( -67.9 - 258.24 ) | 3407.3 ( -1613.39 - 6110.67 ) | 105.01 ( -50.43 - 187.39 ) | -1.07(-1.16 to -0.98) |
| Spain | 2186.39 ( -269.88 - 6472.96 ) | 4.13 ( -0.5 - 12.33 ) | 777.93 ( -79.41 - 2826.14 ) | 0.73 ( -0.07 - 2.67 ) | -5.64(-5.89 to -5.39) |
| Sri Lanka | 6203.53 ( -2672.48 - 11833.65 ) | 66.84 ( -29.17 - 127.4 ) | 11583.68 ( -4070.11 - 23088.03 ) | 44.95 ( -15.84 - 89.04 ) | -1.16(-1.33 to -0.99) |
| Sudan | 6289.29 ( -2649.37 - 11590.39 ) | 77.26 ( -32.91 - 141.12 ) | 5275.88 ( -1324 - 11370.29 ) | 31.32 ( -7.95 - 67.4 ) | -2.85(-3.11 to -2.59) |
| Suriname | 61.84 ( -15.57 - 136.48 ) | 26.04 ( -6.6 - 58.1 ) | 46.14 ( -5.8 - 132.61 ) | 7.7 ( -0.98 - 22.3 ) | -4.17(-4.26 to -4.07) |
| Sweden | 12.48 ( -1.3 - 47.65 ) | 0.08 ( -0.01 - 0.3 ) | 6.61 ( -0.54 - 25.22 ) | 0.03 ( 0 - 0.11 ) | -3.38(-3.49 to -3.28) |
| Switzerland | 7.58 ( -0.81 - 29.33 ) | 0.07 ( -0.01 - 0.27 ) | 5.91 ( -0.53 - 20.08 ) | 0.03 ( 0 - 0.1 ) | -2.84(-2.93 to -2.76) |
| Syrian Arab Republic | 440.62 ( -52.3 - 1464.32 ) | 9.66 ( -1.16 - 32.33 ) | 23.4 ( -2.02 - 87.92 ) | 0.2 ( -0.02 - 0.75 ) | -12.42(-12.67 to -12.16) |
| Taiwan | 496.16 ( -136.53 - 1085.01 ) | 4.05 ( -1.11 - 8.72 ) | 768.37 ( -105.6 - 2191.85 ) | 1.75 ( -0.24 - 4.96 ) | -2.85(-3.04 to -2.67) |
| Tajikistan | 926.3 ( -312.13 - 1811.81 ) | 36.84 ( -12.51 - 71.78 ) | 949.64 ( -172.21 - 2202.5 ) | 19.49 ( -3.64 - 45.77 ) | -2.38(-2.61 to -2.15) |
| Thailand | 19995.23 ( -6824.04 - 39341.9 ) | 65.34 ( -22.47 - 129.52 ) | 25247.03 ( -4671.27 - 62411.58 ) | 23.35 ( -4.32 - 57.64 ) | -3.42(-3.5 to -3.35) |
| Timor-Leste | 207.17 ( -92.75 - 392.12 ) | 92.8 ( -44.21 - 174.22 ) | 387.18 ( -167.02 - 727.48 ) | 51.03 ( -22.32 - 95.57 ) | -1.78(-1.83 to -1.73) |
| Togo | 591.43 ( -277.11 - 1053.81 ) | 56.11 ( -26.86 - 100.76 ) | 1793.23 ( -797.65 - 3291.86 ) | 54.68 ( -24.64 - 100.87 ) | -0.16(-0.3 to -0.03) |
| Tokelau | 0.01 ( 0 - 0.04 ) | 0.82 ( -0.09 - 3.31 ) | 0 ( 0 - 0.01 ) | 0.1 ( -0.01 - 0.39 ) | -6.61(-6.82 to -6.4) |
| Tonga | 18.61 ( -6.47 - 35.11 ) | 37.95 ( -13.46 - 71.84 ) | 15.58 ( -3.49 - 34.1 ) | 20.28 ( -4.56 - 44.33 ) | -1.87(-1.96 to -1.77) |
| Trinidad and Tobago | 4.89 ( -0.51 - 17.21 ) | 0.63 ( -0.07 - 2.25 ) | 1.54 ( -0.16 - 6.17 ) | 0.08 ( -0.01 - 0.33 ) | -7.16(-7.41 to -6.91) |
| Tunisia | 957.33 ( -142.07 - 2741.53 ) | 21.74 ( -3.2 - 62.46 ) | 47.96 ( -4.98 - 183.19 ) | 0.38 ( -0.04 - 1.45 ) | -13.8(-14.26 to -13.35) |
| Türkiye | 6357.9 ( -1028.04 - 16372.54 ) | 20.95 ( -3.41 - 54.35 ) | 1853.33 ( -206.64 - 6696.36 ) | 2.07 ( -0.23 - 7.43 ) | -7.58(-8.06 to -7.09) |
| Turkmenistan | 24.1 ( -2.46 - 83.55 ) | 1.39 ( -0.14 - 4.88 ) | 11.41 ( -1.25 - 46.26 ) | 0.31 ( -0.03 - 1.25 ) | -5.22(-5.8 to -4.64) |
| Tuvalu | 3.67 ( -0.97 - 7.37 ) | 64.3 ( -17.41 - 131.26 ) | 1.28 ( -0.16 - 3.7 ) | 13.53 ( -1.7 - 38.76 ) | -5.27(-5.42 to -5.12) |
| Uganda | 1750.4 ( -827.43 - 3187.37 ) | 30.09 ( -14.41 - 54.31 ) | 3539.37 ( -1723.89 - 6343.52 ) | 26.84 ( -13.42 - 49.62 ) | -0.09(-0.18 to -0.01) |
| Ukraine | 3675.75 ( -515.66 - 10770.68 ) | 5.42 ( -0.75 - 15.83 ) | 1548.08 ( -165.72 - 5023.44 ) | 1.96 ( -0.21 - 6.36 ) | -3.64(-3.93 to -3.36) |
| United Arab Emirates | 3.61 ( -0.34 - 12.61 ) | 0.97 ( -0.09 - 3.35 ) | 1.31 ( -0.12 - 5.02 ) | 0.04 ( 0 - 0.14 ) | -10.56(-11.05 to -10.06) |
| United Kingdom | 60.13 ( -5.99 - 231.72 ) | 0.07 ( -0.01 - 0.25 ) | 27.14 ( -3.1 - 95.27 ) | 0.02 ( 0 - 0.07 ) | -3.98(-4.05 to -3.9) |
| United Republic of Tanzania | 5949.26 ( -2774.16 - 10628.97 ) | 62.16 ( -29.58 - 111.12 ) | 11702.24 ( -5468.38 - 21465.09 ) | 49.9 ( -23.82 - 91.14 ) | -0.52(-0.65 to -0.4) |
| United States of America | 283.41 ( -28.15 - 994.62 ) | 0.09 ( -0.01 - 0.3 ) | 387.43 ( -39.47 - 1436.77 ) | 0.06 ( -0.01 - 0.24 ) | -0.95(-1 to -0.9) |
| United States Virgin Islands | 0.99 ( -0.1 - 3.31 ) | 1.36 ( -0.14 - 4.57 ) | 0.82 ( -0.08 - 2.81 ) | 0.44 ( -0.04 - 1.52 ) | -3.76(-3.82 to -3.7) |
| Uruguay | 72.92 ( -11.63 - 208.78 ) | 1.91 ( -0.3 - 5.48 ) | 35.99 ( -4.57 - 117.72 ) | 0.59 ( -0.07 - 1.94 ) | -3.5(-3.68 to -3.33) |
| Uzbekistan | 2390.24 ( -492.86 - 5683.28 ) | 21.92 ( -4.53 - 52.01 ) | 2189.36 ( -268.78 - 6450.81 ) | 9.78 ( -1.23 - 28.64 ) | -2.74(-2.86 to -2.62) |
| Vanuatu | 28.3 ( -12.62 - 51.24 ) | 54.55 ( -25.1 - 99.76 ) | 63.89 ( -26.76 - 119.24 ) | 44.26 ( -19.07 - 83.73 ) | -0.65(-0.68 to -0.61) |
| Venezuela (Bolivarian Republic of) | 226.01 ( -21.63 - 765.62 ) | 2.56 ( -0.25 - 8.72 ) | 256.38 ( -23.39 - 942.38 ) | 0.9 ( -0.08 - 3.28 ) | -2.76(-3.37 to -2.15) |
| Viet Nam | 23740.71 ( -11112.12 - 43733.84 ) | 65.71 ( -30.87 - 121.16 ) | 26073.62 ( -6699.29 - 57617.9 ) | 30.69 ( -7.97 - 67.76 ) | -2.81(-2.99 to -2.63) |
| Yemen | 2155.85 ( -647.78 - 4252.13 ) | 54.61 ( -16.75 - 108.33 ) | 3625.45 ( -612.69 - 8777.29 ) | 30.05 ( -5.18 - 73 ) | -2.09(-2.43 to -1.76) |
| Zambia | 1344.42 ( -560.1 - 2473.56 ) | 52.49 ( -22.69 - 95.85 ) | 2371.17 ( -978.01 - 4360.34 ) | 38.47 ( -16.68 - 71.21 ) | -0.98(-1.07 to -0.9) |
| Zimbabwe | 2341.35 ( -906.93 - 4343.71 ) | 65.07 ( -25.77 - 122.24 ) | 2732.43 ( -896.5 - 5134.96 ) | 45.96 ( -15.45 - 87.12 ) | -1.14(-1.19 to -1.1) |

**Table S2.** The YLDs, ASYR and EAPC (1990–2021) of cataract burden attributable to smoking among 204 countries and territories.

| **Location** | **1990** | | **2021** | | **1990–2021** |
| --- | --- | --- | --- | --- | --- |
|  | **Number,**  **(95% UI)** | **ASYR**  **per 100 000,**  **(95% UI)** | **Number,**  **(95% UI)** | **ASYR**  **per 100 000,**  **(95% UI)** | **EAPC, %,**  **(95% CI)** |
| Afghanistan | 297.57 ( 181.36 - 466.64 ) | 4.61 ( 2.81 - 7.28 ) | 315.76 ( 205.43 - 463.27 ) | 3.39 ( 2.1 - 5.24 ) | -0.42(-0.9 to 0.06) |
| Albania | 34.93 ( 23.63 - 50.3 ) | 1.75 ( 1.18 - 2.52 ) | 66.63 ( 43.76 - 98.58 ) | 1.55 ( 1.02 - 2.28 ) | -0.36(-0.43 to -0.3) |
| Algeria | 938.78 ( 649 - 1337.35 ) | 8.42 ( 5.78 - 12.03 ) | 1561.07 ( 1062.51 - 2207.29 ) | 4.7 ( 3.26 - 6.69 ) | -1.92(-2.07 to -1.78) |
| American Samoa | 0.89 ( 0.59 - 1.27 ) | 3.73 ( 2.47 - 5.37 ) | 1.32 ( 0.86 - 1.95 ) | 2.66 ( 1.74 - 3.94 ) | -1.12(-1.14 to -1.1) |
| Andorra | 0.7 ( 0.44 - 1.09 ) | 1.2 ( 0.77 - 1.89 ) | 1.38 ( 0.84 - 2.12 ) | 0.91 ( 0.56 - 1.39 ) | -0.92(-0.97 to -0.87) |
| Angola | 74.71 ( 47.58 - 107.92 ) | 1.8 ( 1.17 - 2.56 ) | 126.92 ( 83.57 - 183.93 ) | 0.98 ( 0.65 - 1.42 ) | -1.85(-1.94 to -1.76) |
| Antigua and Barbuda | 0.52 ( 0.32 - 0.82 ) | 0.97 ( 0.61 - 1.53 ) | 0.69 ( 0.43 - 1.09 ) | 0.65 ( 0.41 - 1.04 ) | -1.42(-1.56 to -1.29) |
| Argentina | 538.31 ( 361.13 - 798.17 ) | 1.66 ( 1.11 - 2.48 ) | 567.74 ( 364.47 - 851.96 ) | 1.04 ( 0.67 - 1.56 ) | -1.42(-1.51 to -1.34) |
| Armenia | 98.96 ( 66.25 - 141.63 ) | 3.59 ( 2.4 - 5.04 ) | 141.54 ( 94.33 - 203.05 ) | 3.26 ( 2.18 - 4.67 ) | -0.17(-0.23 to -0.12) |
| Australia | 189.47 ( 127.87 - 270.47 ) | 0.99 ( 0.67 - 1.41 ) | 245.02 ( 159.65 - 368.73 ) | 0.6 ( 0.39 - 0.91 ) | -1.5(-1.57 to -1.43) |
| Austria | 102.34 ( 68.76 - 153.39 ) | 0.96 ( 0.65 - 1.4 ) | 173.52 ( 113.7 - 256.96 ) | 1.09 ( 0.72 - 1.59 ) | 0.51(0.28 to 0.73) |
| Azerbaijan | 151.14 ( 98.77 - 222.16 ) | 2.96 ( 1.92 - 4.34 ) | 319.11 ( 210.81 - 457.34 ) | 3.16 ( 2.08 - 4.47 ) | 0.42(0.15 to 0.68) |
| Bahamas | 1.34 ( 0.82 - 2.07 ) | 0.87 ( 0.53 - 1.36 ) | 2.67 ( 1.52 - 4.33 ) | 0.67 ( 0.39 - 1.1 ) | -0.84(-0.94 to -0.74) |
| Bahrain | 10.95 ( 6.9 - 16.82 ) | 6.31 ( 4 - 9.81 ) | 34.63 ( 21.29 - 53.93 ) | 3.72 ( 2.35 - 5.85 ) | -1.89(-1.99 to -1.79) |
| Bangladesh | 4296.53 ( 3009.61 - 5873.01 ) | 9.89 ( 6.92 - 13.49 ) | 8228.24 ( 5570.42 - 11770.48 ) | 6.09 ( 4.15 - 8.68 ) | -1.08(-1.27 to -0.88) |
| Barbados | 0.96 ( 0.61 - 1.45 ) | 0.32 ( 0.2 - 0.48 ) | 1.1 ( 0.65 - 1.73 ) | 0.22 ( 0.13 - 0.34 ) | -1.45(-1.59 to -1.31) |
| Belarus | 200.2 ( 131.1 - 292.14 ) | 1.57 ( 1.04 - 2.29 ) | 199.41 ( 134.77 - 289.71 ) | 1.28 ( 0.87 - 1.85 ) | -0.55(-0.61 to -0.5) |
| Belgium | 232.13 ( 157.31 - 344.05 ) | 1.61 ( 1.09 - 2.38 ) | 220.64 ( 143.2 - 335.38 ) | 1.05 ( 0.68 - 1.57 ) | -1.32(-1.38 to -1.26) |
| Belize | 1.34 ( 0.88 - 1.93 ) | 1.44 ( 0.95 - 2.09 ) | 2.42 ( 1.6 - 3.59 ) | 0.83 ( 0.53 - 1.26 ) | -1.66(-1.82 to -1.5) |
| Benin | 19.82 ( 13.14 - 28.98 ) | 1.02 ( 0.67 - 1.5 ) | 50.58 ( 32.68 - 76.24 ) | 0.96 ( 0.61 - 1.44 ) | -0.52(-0.89 to -0.15) |
| Bermuda | 0.63 ( 0.38 - 0.99 ) | 1.01 ( 0.61 - 1.58 ) | 1.1 ( 0.65 - 1.72 ) | 0.83 ( 0.5 - 1.3 ) | -0.64(-0.72 to -0.57) |
| Bhutan | 4.29 ( 2.39 - 6.59 ) | 2.05 ( 1.15 - 3.2 ) | 5.72 ( 3.56 - 8.62 ) | 0.99 ( 0.61 - 1.5 ) | -2.6(-2.69 to -2.52) |
| Bolivia (Plurinational State of) | 106.61 ( 70.78 - 155.35 ) | 3.4 ( 2.25 - 4.99 ) | 164.22 ( 107.6 - 238.37 ) | 1.81 ( 1.18 - 2.64 ) | -1.83(-2.01 to -1.64) |
| Bosnia and Herzegovina | 56.69 ( 38.1 - 81.02 ) | 1.39 ( 0.93 - 1.99 ) | 67.86 ( 44.57 - 100 ) | 1.14 ( 0.76 - 1.67 ) | -0.63(-0.94 to -0.31) |
| Botswana | 23.32 ( 15.78 - 33.51 ) | 4.2 ( 2.87 - 5.99 ) | 31.25 ( 21.18 - 44.49 ) | 2.09 ( 1.41 - 2.95 ) | -2.27(-2.36 to -2.18) |
| Brazil | 5307.1 ( 3582.66 - 7494.74 ) | 6.09 ( 4.1 - 8.63 ) | 6260.05 ( 4018.23 - 9439.16 ) | 2.5 ( 1.61 - 3.77 ) | -2.64(-2.95 to -2.33) |
| Brunei Darussalam | 1.72 ( 1.14 - 2.49 ) | 1.46 ( 0.98 - 2.12 ) | 2.41 ( 1.53 - 3.56 ) | 0.64 ( 0.41 - 0.94 ) | -2.64(-2.78 to -2.51) |
| Bulgaria | 89.35 ( 59.35 - 129.83 ) | 0.74 ( 0.49 - 1.09 ) | 62.3 ( 40.13 - 92.47 ) | 0.5 ( 0.32 - 0.75 ) | -1.23(-1.41 to -1.05) |
| Burkina Faso | 19.41 ( 12.36 - 29.16 ) | 0.47 ( 0.3 - 0.72 ) | 71.38 ( 45.91 - 107.17 ) | 0.72 ( 0.45 - 1.11 ) | 0.14(-0.3 to 0.58) |
| Burundi | 13.47 ( 8.98 - 19.16 ) | 0.57 ( 0.39 - 0.82 ) | 14.65 ( 9.12 - 22.63 ) | 0.28 ( 0.18 - 0.43 ) | -2.55(-3.01 to -2.1) |
| Cabo Verde | 3.58 ( 2.38 - 5.11 ) | 1.62 ( 1.08 - 2.3 ) | 3.02 ( 1.93 - 4.43 ) | 0.64 ( 0.41 - 0.95 ) | -3.09(-3.25 to -2.94) |
| Cambodia | 672.42 ( 475.41 - 944.95 ) | 15.66 ( 11.19 - 21.83 ) | 1039.56 ( 698.08 - 1471.55 ) | 8.84 ( 5.93 - 12.47 ) | -2.31(-2.56 to -2.05) |
| Cameroon | 38.4 ( 25.67 - 57.4 ) | 0.89 ( 0.61 - 1.33 ) | 74.81 ( 48.07 - 111.8 ) | 0.58 ( 0.37 - 0.87 ) | -1.39(-1.5 to -1.27) |
| Canada | 414.21 ( 279.79 - 599.44 ) | 1.29 ( 0.88 - 1.86 ) | 518.44 ( 326.27 - 800.74 ) | 0.76 ( 0.48 - 1.18 ) | -1.78(-1.89 to -1.67) |
| Central African Republic | 3.76 ( 2.38 - 5.47 ) | 0.4 ( 0.25 - 0.58 ) | 7.22 ( 4.38 - 10.75 ) | 0.32 ( 0.2 - 0.47 ) | -0.68(-0.93 to -0.42) |
| Chad | 42.58 ( 25.78 - 61.3 ) | 1.53 ( 0.94 - 2.21 ) | 69.76 ( 44.39 - 103.33 ) | 1.25 ( 0.8 - 1.88 ) | -0.79(-0.87 to -0.71) |
| Chile | 222.52 ( 150.14 - 319.31 ) | 2.08 ( 1.39 - 3.01 ) | 277.8 ( 181.03 - 410.53 ) | 1.14 ( 0.74 - 1.67 ) | -2.08(-2.13 to -2.03) |
| China | 31757.73 ( 22343.97 - 44077.98 ) | 3.95 ( 2.79 - 5.45 ) | 60057.78 ( 41169.59 - 84681.39 ) | 2.83 ( 1.94 - 4 ) | -0.61(-0.84 to -0.37) |
| Colombia | 393.19 ( 260.58 - 560.37 ) | 2.22 ( 1.47 - 3.15 ) | 497.1 ( 316.47 - 742.65 ) | 0.9 ( 0.57 - 1.34 ) | -3(-3.1 to -2.89) |
| Comoros | 4.61 ( 2.94 - 6.89 ) | 2.42 ( 1.56 - 3.62 ) | 5.86 ( 3.83 - 8.55 ) | 1.23 ( 0.8 - 1.81 ) | -2.31(-2.38 to -2.24) |
| Congo | 8.76 ( 5.71 - 12.52 ) | 0.82 ( 0.53 - 1.18 ) | 18.54 ( 11.57 - 27.03 ) | 0.68 ( 0.43 - 0.99 ) | -0.33(-0.51 to -0.14) |
| Cook Islands | 0.71 ( 0.46 - 1.03 ) | 5.42 ( 3.56 - 7.9 ) | 0.79 ( 0.52 - 1.2 ) | 3.06 ( 2 - 4.64 ) | -1.86(-1.91 to -1.81) |
| Costa Rica | 54.82 ( 36.53 - 79.51 ) | 3.17 ( 2.11 - 4.61 ) | 92.63 ( 60.18 - 137.77 ) | 1.69 ( 1.09 - 2.51 ) | -2(-2.09 to -1.91) |
| Cote d'Ivoire | 28.37 ( 18.95 - 41.24 ) | 0.81 ( 0.53 - 1.18 ) | 152.29 ( 102.2 - 232.04 ) | 1.29 ( 0.86 - 1.98 ) | -0.74(-0.85 to -0.64) |
| Croatia | 82.12 ( 56.05 - 117.23 ) | 1.37 ( 0.93 - 1.95 ) | 83.5 ( 54.04 - 121.45 ) | 1 ( 0.66 - 1.46 ) | -2.36(-2.49 to -2.23) |
| Cuba | 423.02 ( 287.66 - 617.91 ) | 4.14 ( 2.81 - 6.02 ) | 402.68 ( 264.42 - 606.6 ) | 2.09 ( 1.38 - 3.15 ) | -0.59(-0.65 to -0.53) |
| Cyprus | 10.78 ( 7.22 - 15.39 ) | 1.35 ( 0.89 - 1.92 ) | 22 ( 14.61 - 32.41 ) | 1.1 ( 0.73 - 1.62 ) | -0.97(-1.04 to -0.9) |
| Czechia | 162.97 ( 109.12 - 235.9 ) | 1.2 ( 0.8 - 1.74 ) | 178.97 ( 118.96 - 266.41 ) | 0.89 ( 0.59 - 1.32 ) | -0.53(-1.4 to 0.34) |
| Democratic People's Republic of Korea | 99.19 ( 62.91 - 146.16 ) | 0.65 ( 0.42 - 0.96 ) | 139.75 ( 83.99 - 219.74 ) | 0.43 ( 0.26 - 0.67 ) | -1.63(-1.71 to -1.55) |
| Democratic Republic of the Congo | 19.09 ( 12.61 - 28.26 ) | 0.13 ( 0.08 - 0.19 ) | 38.29 ( 24.63 - 57.35 ) | 0.1 ( 0.06 - 0.15 ) | -0.69(-0.81 to -0.57) |
| Denmark | 178.05 ( 118.51 - 253.79 ) | 2.24 ( 1.5 - 3.13 ) | 158.76 ( 102.71 - 243.64 ) | 1.38 ( 0.91 - 2.1 ) | -1.63(-1.75 to -1.51) |
| Djibouti | 5.15 ( 3.22 - 7.62 ) | 3.64 ( 2.35 - 5.35 ) | 13.27 ( 8.51 - 19.64 ) | 2.06 ( 1.32 - 3.02 ) | -1.75(-1.78 to -1.73) |
| Dominica | 0.5 ( 0.32 - 0.75 ) | 0.84 ( 0.53 - 1.27 ) | 0.44 ( 0.27 - 0.7 ) | 0.53 ( 0.32 - 0.84 ) | -1.45(-1.71 to -1.2) |
| Dominican Republic | 94.98 ( 59.99 - 142.49 ) | 2.77 ( 1.74 - 4.15 ) | 149.43 ( 92.62 - 223.05 ) | 1.53 ( 0.94 - 2.29 ) | -1.91(-2.03 to -1.78) |
| Ecuador | 147.2 ( 96.66 - 213.42 ) | 2.87 ( 1.86 - 4.14 ) | 188.37 ( 120.98 - 278.36 ) | 1.15 ( 0.74 - 1.7 ) | -2.99(-3.12 to -2.86) |
| Egypt | 1388.97 ( 918.3 - 1968.27 ) | 5.74 ( 3.87 - 8.16 ) | 2814.42 ( 1932.27 - 4069.21 ) | 4.97 ( 3.44 - 7.16 ) | -0.52(-0.62 to -0.42) |
| El Salvador | 52.31 ( 31.12 - 82.01 ) | 1.76 ( 1.04 - 2.79 ) | 64.37 ( 38.03 - 100.24 ) | 1.04 ( 0.62 - 1.62 ) | -1.48(-1.58 to -1.39) |
| Equatorial Guinea | 8.63 ( 5.07 - 13.4 ) | 4.27 ( 2.5 - 6.52 ) | 7.14 ( 4.24 - 11 ) | 1.18 ( 0.71 - 1.83 ) | -4.5(-4.7 to -4.3) |
| Eritrea | 17.89 ( 11.1 - 28.66 ) | 1.25 ( 0.79 - 2 ) | 21.47 ( 13.8 - 33.76 ) | 0.63 ( 0.41 - 1 ) | -2.2(-2.32 to -2.09) |
| Estonia | 17.67 ( 11.41 - 25.78 ) | 0.87 ( 0.56 - 1.27 ) | 18.45 ( 11.99 - 27.33 ) | 0.77 ( 0.5 - 1.13 ) | -0.44(-0.63 to -0.24) |
| Eswatini | 6.36 ( 4.24 - 9.04 ) | 2.42 ( 1.58 - 3.37 ) | 5.52 ( 3.53 - 8.38 ) | 1.09 ( 0.69 - 1.62 ) | -2.68(-2.74 to -2.62) |
| Ethiopia | 450.48 ( 301.75 - 627.21 ) | 2.03 ( 1.37 - 2.8 ) | 698.66 ( 480.05 - 979.29 ) | 1.46 ( 1 - 2.07 ) | -0.74(-0.88 to -0.6) |
| Fiji | 21.05 ( 14.39 - 29.09 ) | 5.48 ( 3.7 - 7.62 ) | 25.79 ( 17.76 - 36.83 ) | 3.13 ( 2.14 - 4.52 ) | -1.65(-1.73 to -1.57) |
| Finland | 52.33 ( 35.45 - 77.3 ) | 0.79 ( 0.53 - 1.15 ) | 62.07 ( 38.62 - 96.69 ) | 0.6 ( 0.38 - 0.91 ) | -0.63(-0.75 to -0.51) |
| France | 963.7 ( 646.37 - 1396.76 ) | 1.23 ( 0.82 - 1.8 ) | 1006.53 ( 653.05 - 1503.71 ) | 0.85 ( 0.56 - 1.27 ) | -1.19(-1.29 to -1.1) |
| Gabon | 3.61 ( 2.36 - 5.26 ) | 0.62 ( 0.41 - 0.9 ) | 4.63 ( 2.97 - 6.81 ) | 0.41 ( 0.26 - 0.61 ) | -1.27(-1.32 to -1.22) |
| Gambia | 15.76 ( 10.79 - 22.42 ) | 4.1 ( 2.77 - 5.81 ) | 17.66 ( 11.99 - 25.46 ) | 1.73 ( 1.15 - 2.48 ) | -2.9(-3.02 to -2.79) |
| Georgia | 164.17 ( 108.51 - 236.21 ) | 2.6 ( 1.73 - 3.71 ) | 176.02 ( 117.06 - 255.93 ) | 3.04 ( 2.05 - 4.38 ) | 0.97(0.82 to 1.13) |
| Germany | 1552.92 ( 1042.85 - 2261.3 ) | 1.29 ( 0.87 - 1.89 ) | 1421.69 ( 905.61 - 2134.86 ) | 0.83 ( 0.53 - 1.24 ) | -1.44(-1.52 to -1.36) |
| Ghana | 38.56 ( 25.44 - 57.2 ) | 0.76 ( 0.5 - 1.14 ) | 136.37 ( 85.54 - 208.54 ) | 0.89 ( 0.56 - 1.37 ) | 0.58(0.49 to 0.67) |
| Greece | 330.75 ( 223.5 - 479.97 ) | 2.29 ( 1.57 - 3.32 ) | 281.55 ( 187.03 - 417.94 ) | 1.3 ( 0.87 - 1.93 ) | -1.86(-1.94 to -1.78) |
| Greenland | 0.59 ( 0.38 - 0.91 ) | 1.56 ( 1 - 2.38 ) | 0.77 ( 0.5 - 1.13 ) | 1.1 ( 0.7 - 1.6 ) | -1.28(-1.33 to -1.24) |
| Grenada | 0.71 ( 0.44 - 1.12 ) | 1.02 ( 0.64 - 1.6 ) | 0.57 ( 0.36 - 0.91 ) | 0.49 ( 0.31 - 0.78 ) | -2.58(-2.75 to -2.41) |
| Guam | 2.52 ( 1.64 - 3.9 ) | 2.94 ( 1.91 - 4.55 ) | 5.33 ( 3.37 - 8.09 ) | 2.49 ( 1.58 - 3.78 ) | -0.55(-0.6 to -0.51) |
| Guatemala | 61.91 ( 39.84 - 92.47 ) | 1.97 ( 1.27 - 2.91 ) | 110.49 ( 69.6 - 163.39 ) | 1.03 ( 0.65 - 1.53 ) | -2.05(-2.09 to -2.02) |
| Guinea | 53.67 ( 35.02 - 74.94 ) | 1.67 ( 1.09 - 2.35 ) | 83.16 ( 54.69 - 116.52 ) | 1.47 ( 0.97 - 2.1 ) | -0.52(-0.67 to -0.37) |
| Guinea-Bissau | 1.92 ( 1.2 - 2.94 ) | 0.49 ( 0.31 - 0.76 ) | 4.21 ( 2.73 - 6.37 ) | 0.55 ( 0.34 - 0.84 ) | 0.47(0.38 to 0.55) |
| Guyana | 4.85 ( 3.14 - 7.1 ) | 1.26 ( 0.8 - 1.88 ) | 4.2 ( 2.69 - 6.15 ) | 0.66 ( 0.42 - 0.97 ) | -1.91(-2.04 to -1.77) |
| Haiti | 38.54 ( 23.94 - 58.52 ) | 1.15 ( 0.7 - 1.75 ) | 41.57 ( 25.46 - 62.28 ) | 0.57 ( 0.34 - 0.91 ) | -2.34(-2.51 to -2.18) |
| Honduras | 38.19 ( 25.24 - 55.03 ) | 1.91 ( 1.25 - 2.76 ) | 63.44 ( 41.74 - 96.93 ) | 1.02 ( 0.67 - 1.55 ) | -2.1(-2.21 to -2) |
| Hungary | 334.73 ( 227.31 - 474.68 ) | 2.35 ( 1.6 - 3.31 ) | 210.99 ( 139 - 307.28 ) | 1.22 ( 0.81 - 1.76 ) | -2.27(-2.37 to -2.16) |
| Iceland | 4.34 ( 2.79 - 6.56 ) | 1.57 ( 1.02 - 2.36 ) | 5.41 ( 3.42 - 8.35 ) | 0.99 ( 0.64 - 1.54 ) | -1.51(-1.62 to -1.39) |
| India | 47793.42 ( 33912.24 - 65346.48 ) | 11.28 ( 8.01 - 15.39 ) | 59155.05 ( 42040.93 - 82710.53 ) | 5.21 ( 3.69 - 7.35 ) | -2.44(-2.54 to -2.33) |
| Indonesia | 7284.97 ( 5163.68 - 9974.18 ) | 7.7 ( 5.47 - 10.54 ) | 13487.49 ( 9538.02 - 18757.02 ) | 5.72 ( 4.06 - 7.81 ) | -1.14(-1.34 to -0.94) |
| Iran (Islamic Republic of) | 844.28 ( 568.33 - 1249.86 ) | 3.2 ( 2.18 - 4.69 ) | 1995.03 ( 1352.91 - 2971.67 ) | 2.55 ( 1.74 - 3.82 ) | -0.75(-0.83 to -0.67) |
| Iraq | 851.33 ( 588.27 - 1188.49 ) | 11.31 ( 7.8 - 15.94 ) | 1111.9 ( 755.54 - 1632.99 ) | 5.07 ( 3.44 - 7.55 ) | -2.67(-2.72 to -2.62) |
| Ireland | 73.1 ( 50.31 - 105.22 ) | 1.85 ( 1.28 - 2.68 ) | 64.5 ( 41.7 - 95.84 ) | 0.84 ( 0.55 - 1.25 ) | -2.91(-3.04 to -2.78) |
| Israel | 59.62 ( 40.31 - 88.91 ) | 1.28 ( 0.85 - 1.9 ) | 87.57 ( 57.73 - 131.58 ) | 0.75 ( 0.5 - 1.12 ) | -1.74(-1.79 to -1.69) |
| Italy | 2538.3 ( 1744.29 - 3570.79 ) | 3.09 ( 2.14 - 4.34 ) | 2078.2 ( 1400.59 - 3070.95 ) | 1.68 ( 1.14 - 2.46 ) | -1.97(-2.06 to -1.89) |
| Jamaica | 27.15 ( 18.11 - 39.97 ) | 1.54 ( 1.02 - 2.25 ) | 26.69 ( 16.91 - 42.03 ) | 0.87 ( 0.55 - 1.34 ) | -2.09(-2.18 to -2) |
| Japan | 2052.17 ( 1451.85 - 2886.94 ) | 1.22 ( 0.87 - 1.72 ) | 2050.37 ( 1354.87 - 3014.77 ) | 0.69 ( 0.47 - 0.97 ) | -2(-2.06 to -1.93) |
| Jordan | 51.83 ( 35.1 - 74.68 ) | 4.38 ( 2.98 - 6.35 ) | 166.18 ( 109.48 - 236.03 ) | 2.37 ( 1.57 - 3.37 ) | -2.08(-2.17 to -2) |
| Kazakhstan | 264.81 ( 175.78 - 386.21 ) | 2 ( 1.33 - 2.92 ) | 298.28 ( 195.72 - 432.11 ) | 1.56 ( 1.03 - 2.26 ) | -0.74(-0.88 to -0.59) |
| Kenya | 371.73 ( 260.47 - 526.34 ) | 4.32 ( 3.02 - 6.09 ) | 413.96 ( 285.4 - 583.3 ) | 1.68 ( 1.16 - 2.38 ) | -3.23(-3.51 to -2.95) |
| Kiribati | 3.12 ( 2.18 - 4.37 ) | 8.26 ( 5.82 - 11.65 ) | 5.22 ( 3.56 - 7.31 ) | 7.08 ( 4.89 - 9.85 ) | -0.66(-0.96 to -0.36) |
| Kuwait | 36.56 ( 24.4 - 53.06 ) | 6.01 ( 4.02 - 8.62 ) | 113.92 ( 75.44 - 164.3 ) | 3.97 ( 2.6 - 5.79 ) | -1.43(-1.51 to -1.36) |
| Kyrgyzstan | 78.61 ( 54.96 - 111.62 ) | 2.63 ( 1.85 - 3.78 ) | 167.46 ( 112.15 - 235.1 ) | 3.44 ( 2.28 - 4.85 ) | 1.77(1.47 to 2.07) |
| Lao People's Democratic Republic | 36.97 ( 24.79 - 53.35 ) | 1.99 ( 1.33 - 2.83 ) | 61.97 ( 40.92 - 90.09 ) | 1.5 ( 1.01 - 2.19 ) | -0.85(-0.89 to -0.81) |
| Latvia | 45.77 ( 31.49 - 65.68 ) | 1.3 ( 0.89 - 1.86 ) | 31.43 ( 20.78 - 46.11 ) | 0.9 ( 0.61 - 1.31 ) | -1.08(-1.16 to -0.99) |
| Lebanon | 255.02 ( 165.43 - 359.58 ) | 12.04 ( 7.84 - 16.97 ) | 498.83 ( 333.74 - 721.58 ) | 8.27 ( 5.54 - 11.89 ) | -1.34(-1.54 to -1.13) |
| Lesotho | 35.03 ( 22.59 - 49.48 ) | 4.32 ( 2.77 - 6.32 ) | 35.14 ( 22.92 - 50.93 ) | 3.41 ( 2.25 - 5.05 ) | -0.58(-0.66 to -0.51) |
| Liberia | 15.38 ( 10.14 - 22.57 ) | 1.32 ( 0.88 - 1.95 ) | 16.51 ( 10.79 - 23.51 ) | 0.72 ( 0.46 - 1.05 ) | -2.22(-2.34 to -2.1) |
| Libya | 142.67 ( 96.99 - 203.08 ) | 7.54 ( 5.12 - 10.67 ) | 211.22 ( 146.56 - 296.54 ) | 4 ( 2.75 - 5.52 ) | -2.05(-2.23 to -1.86) |
| Lithuania | 57.45 ( 38.56 - 82.45 ) | 1.29 ( 0.87 - 1.86 ) | 47.04 ( 30.02 - 67.73 ) | 0.89 ( 0.58 - 1.29 ) | -1.24(-1.27 to -1.21) |
| Luxembourg | 6.31 ( 3.82 - 9.73 ) | 1.21 ( 0.73 - 1.86 ) | 9.19 ( 5.52 - 14.21 ) | 0.9 ( 0.55 - 1.36 ) | -0.99(-1.03 to -0.95) |
| Madagascar | 50.15 ( 34.04 - 70.55 ) | 1.03 ( 0.7 - 1.47 ) | 40.2 ( 27.25 - 56.69 ) | 0.36 ( 0.25 - 0.52 ) | -3.55(-3.81 to -3.28) |
| Malawi | 105.31 ( 69.09 - 149.51 ) | 2.88 ( 1.89 - 4.07 ) | 126.48 ( 81.75 - 180.04 ) | 1.72 ( 1.1 - 2.45 ) | -1.51(-1.58 to -1.44) |
| Malaysia | 830.71 ( 592.5 - 1166.48 ) | 9.07 ( 6.43 - 12.65 ) | 1028.57 ( 726.21 - 1414.03 ) | 3.73 ( 2.6 - 5.1 ) | -3.07(-3.18 to -2.95) |
| Maldives | 3.1 ( 2.05 - 4.42 ) | 4.4 ( 2.97 - 6.25 ) | 5.03 ( 3.36 - 7.28 ) | 1.59 ( 1.06 - 2.31 ) | -3.56(-3.82 to -3.3) |
| Mali | 66.23 ( 44.23 - 93.67 ) | 1.74 ( 1.16 - 2.51 ) | 210.4 ( 143.11 - 294.13 ) | 2.45 ( 1.65 - 3.47 ) | 1.08(0.9 to 1.26) |
| Malta | 5.84 ( 3.84 - 8.43 ) | 1.38 ( 0.91 - 1.99 ) | 6.75 ( 4.36 - 10.17 ) | 0.83 ( 0.54 - 1.24 ) | -1.82(-1.9 to -1.74) |
| Marshall Islands | 0.63 ( 0.39 - 0.93 ) | 3.62 ( 2.26 - 5.48 ) | 0.87 ( 0.55 - 1.33 ) | 2.47 ( 1.56 - 3.87 ) | -1.22(-1.23 to -1.21) |
| Mauritania | 17.32 ( 12.18 - 23.91 ) | 1.62 ( 1.13 - 2.25 ) | 21.18 ( 14.39 - 30.13 ) | 0.91 ( 0.62 - 1.3 ) | -1.9(-1.93 to -1.87) |
| Mauritius | 28.6 ( 19.75 - 40.73 ) | 4.02 ( 2.8 - 5.73 ) | 50.11 ( 33.06 - 73.08 ) | 2.73 ( 1.81 - 3.97 ) | -1.21(-1.6 to -0.82) |
| Mexico | 1599.76 ( 1107.94 - 2218.55 ) | 4.02 ( 2.79 - 5.53 ) | 1502.21 ( 1027 - 2171.75 ) | 1.22 ( 0.83 - 1.77 ) | -4.26(-4.41 to -4.11) |
| Micronesia (Federated States of) | 2.95 ( 1.95 - 4.22 ) | 5.81 ( 3.89 - 8.29 ) | 3.15 ( 2.06 - 4.64 ) | 3.89 ( 2.57 - 5.69 ) | -1.37(-1.4 to -1.34) |
| Monaco | 0.76 ( 0.47 - 1.22 ) | 1.25 ( 0.77 - 1.97 ) | 0.8 ( 0.47 - 1.34 ) | 0.95 ( 0.56 - 1.56 ) | -0.93(-0.96 to -0.89) |
| Mongolia | 20.03 ( 13.13 - 28.29 ) | 1.88 ( 1.25 - 2.63 ) | 38.84 ( 26.04 - 54.94 ) | 1.63 ( 1.11 - 2.31 ) | -0.11(-0.24 to 0.03) |
| Montenegro | 9.18 ( 5.88 - 14.18 ) | 1.46 ( 0.93 - 2.24 ) | 11.56 ( 7.44 - 17.47 ) | 1.19 ( 0.76 - 1.77 ) | -0.85(-1.1 to -0.6) |
| Morocco | 523.33 ( 352.52 - 738.66 ) | 3.65 ( 2.45 - 5.12 ) | 617.71 ( 410.95 - 909.63 ) | 1.74 ( 1.15 - 2.61 ) | -2.48(-2.64 to -2.33) |
| Mozambique | 129.77 ( 86.14 - 181.06 ) | 2.17 ( 1.46 - 3.09 ) | 148.18 ( 98.67 - 218.73 ) | 1.3 ( 0.87 - 1.89 ) | -1.48(-1.58 to -1.38) |
| Myanmar | 3708.4 ( 2516.16 - 5081.96 ) | 17.49 ( 11.77 - 23.96 ) | 2406.75 ( 1622.82 - 3355.92 ) | 5.18 ( 3.54 - 7.15 ) | -4.33(-4.48 to -4.18) |
| Namibia | 23.79 ( 15.82 - 33.5 ) | 4.23 ( 2.86 - 5.98 ) | 23.74 ( 15.9 - 34.02 ) | 1.95 ( 1.29 - 2.77 ) | -2.74(-2.86 to -2.62) |
| Nauru | 0.26 ( 0.17 - 0.38 ) | 5.3 ( 3.57 - 7.91 ) | 0.21 ( 0.14 - 0.31 ) | 3.4 ( 2.27 - 4.96 ) | -1.48(-1.62 to -1.34) |
| Nepal | 645.31 ( 457.77 - 877.81 ) | 8 ( 5.58 - 10.85 ) | 851.12 ( 583.18 - 1204.89 ) | 3.9 ( 2.65 - 5.49 ) | -2.86(-3.02 to -2.69) |
| Netherlands | 204.34 ( 137.31 - 303.74 ) | 1.06 ( 0.72 - 1.58 ) | 238.01 ( 152.48 - 360.1 ) | 0.72 ( 0.47 - 1.1 ) | -0.94(-1.05 to -0.83) |
| New Zealand | 49.17 ( 32.69 - 71.34 ) | 1.28 ( 0.85 - 1.86 ) | 60.63 ( 38.72 - 94.63 ) | 0.75 ( 0.48 - 1.16 ) | -1.71(-1.84 to -1.57) |
| Nicaragua | 42.16 ( 27.53 - 61.85 ) | 2.77 ( 1.8 - 4.12 ) | 72.19 ( 47.17 - 104.8 ) | 1.49 ( 0.96 - 2.18 ) | -1.74(-1.96 to -1.53) |
| Niger | 23.87 ( 15.77 - 33.66 ) | 0.86 ( 0.56 - 1.21 ) | 68.48 ( 44.47 - 99.69 ) | 0.87 ( 0.57 - 1.26 ) | -0.01(-0.09 to 0.06) |
| Nigeria | 876.36 ( 595.52 - 1237.05 ) | 1.99 ( 1.37 - 2.81 ) | 1436.39 ( 964.94 - 2087.14 ) | 1.49 ( 1 - 2.21 ) | -0.99(-1.3 to -0.68) |
| Niue | 0.07 ( 0.05 - 0.11 ) | 3.54 ( 2.23 - 5.38 ) | 0.05 ( 0.03 - 0.08 ) | 2.42 ( 1.49 - 3.69 ) | -1.32(-1.37 to -1.27) |
| North Macedonia | 29.93 ( 19.55 - 43.96 ) | 1.59 ( 1.04 - 2.32 ) | 36.22 ( 23.82 - 52.43 ) | 1.12 ( 0.74 - 1.62 ) | -1.32(-1.39 to -1.25) |
| Northern Mariana Islands | 0.7 ( 0.45 - 0.99 ) | 3.6 ( 2.38 - 5.17 ) | 1.45 ( 0.9 - 2.17 ) | 2.58 ( 1.61 - 3.81 ) | -1.09(-1.13 to -1.05) |
| Norway | 66.88 ( 46.19 - 97.75 ) | 1.07 ( 0.75 - 1.57 ) | 56.83 ( 38.43 - 83.86 ) | 0.63 ( 0.42 - 0.94 ) | -1.97(-2.11 to -1.82) |
| Oman | 51.13 ( 34.77 - 71.92 ) | 6.69 ( 4.52 - 9.42 ) | 87.92 ( 58.68 - 126.13 ) | 3.7 ( 2.43 - 5.29 ) | -2.05(-2.12 to -1.98) |
| Pakistan | 9761.32 ( 6922.44 - 13356.35 ) | 17.83 ( 12.63 - 24.44 ) | 11369.22 ( 7910.57 - 15793.13 ) | 9.65 ( 6.73 - 13.33 ) | -2.39(-2.65 to -2.14) |
| Palau | 0.33 ( 0.21 - 0.51 ) | 3.34 ( 2.07 - 5.16 ) | 0.57 ( 0.36 - 0.87 ) | 2.42 ( 1.53 - 3.64 ) | -1.12(-1.17 to -1.07) |
| Palestine | 81.76 ( 54.97 - 121.78 ) | 9.75 ( 6.54 - 14.49 ) | 144.13 ( 96.95 - 214.19 ) | 5.73 ( 3.85 - 8.48 ) | -1.77(-1.85 to -1.69) |
| Panama | 65.05 ( 44.24 - 91.48 ) | 4.47 ( 2.99 - 6.27 ) | 91.32 ( 57.59 - 140.4 ) | 2.06 ( 1.3 - 3.17 ) | -2.61(-2.72 to -2.5) |
| Papua New Guinea | 122.66 ( 77.17 - 176.41 ) | 6.13 ( 3.86 - 8.81 ) | 280.82 ( 180.84 - 424.39 ) | 4.85 ( 3.1 - 7.4 ) | -0.87(-1.01 to -0.72) |
| Paraguay | 149.9 ( 102.65 - 209.65 ) | 7.09 ( 4.81 - 10.02 ) | 148.12 ( 94.39 - 223.8 ) | 2.64 ( 1.69 - 4.01 ) | -3.46(-3.61 to -3.31) |
| Peru | 345.51 ( 221.59 - 521.12 ) | 3.02 ( 1.92 - 4.62 ) | 660.48 ( 401.66 - 1040.41 ) | 1.99 ( 1.2 - 3.14 ) | -2.14(-2.42 to -1.86) |
| Philippines | 2429.73 ( 1689.53 - 3358.59 ) | 8.56 ( 5.88 - 11.7 ) | 3346.32 ( 2294.21 - 4689.23 ) | 4.18 ( 2.88 - 5.85 ) | -2.31(-2.41 to -2.22) |
| Poland | 635.63 ( 437.38 - 904.89 ) | 1.46 ( 1.01 - 2.09 ) | 530.18 ( 358.64 - 765.79 ) | 0.79 ( 0.54 - 1.13 ) | -2.15(-2.22 to -2.09) |
| Portugal | 123.77 ( 82.72 - 179.65 ) | 0.94 ( 0.63 - 1.37 ) | 102.87 ( 66.61 - 155.22 ) | 0.53 ( 0.35 - 0.79 ) | -2.02(-2.13 to -1.9) |
| Puerto Rico | 41.8 ( 25.11 - 67.38 ) | 1.15 ( 0.69 - 1.84 ) | 53.66 ( 31.96 - 92.26 ) | 0.77 ( 0.45 - 1.27 ) | -1.44(-1.5 to -1.37) |
| Qatar | 6.28 ( 3.85 - 9.55 ) | 4.32 ( 2.7 - 6.56 ) | 33.56 ( 20.8 - 51.54 ) | 2.51 ( 1.54 - 3.76 ) | -1.83(-1.96 to -1.7) |
| Republic of Korea | 496.77 ( 327.91 - 732.01 ) | 1.6 ( 1.06 - 2.29 ) | 640.7 ( 422.44 - 931.54 ) | 0.72 ( 0.48 - 1.03 ) | -2.79(-2.9 to -2.68) |
| Republic of Moldova | 144.77 ( 94.65 - 212.84 ) | 3.23 ( 2.1 - 4.76 ) | 177.65 ( 111.29 - 263.67 ) | 3.06 ( 1.93 - 4.54 ) | 0.02(-0.12 to 0.16) |
| Romania | 262.37 ( 175.52 - 377.1 ) | 0.95 ( 0.64 - 1.38 ) | 200.71 ( 133.59 - 289.85 ) | 0.6 ( 0.4 - 0.88 ) | -1.76(-1.87 to -1.64) |
| Russian Federation | 1564.29 ( 1091.14 - 2225.54 ) | 0.87 ( 0.61 - 1.24 ) | 2150.81 ( 1492.43 - 3093.84 ) | 0.94 ( 0.66 - 1.35 ) | 0.27(-0.11 to 0.65) |
| Rwanda | 42.42 ( 27.92 - 61.26 ) | 1.69 ( 1.11 - 2.44 ) | 63.04 ( 38.52 - 95.08 ) | 1.13 ( 0.68 - 1.71 ) | -1.41(-1.51 to -1.31) |
| Saint Kitts and Nevis | 0.31 ( 0.19 - 0.5 ) | 0.82 ( 0.5 - 1.31 ) | 0.28 ( 0.17 - 0.44 ) | 0.42 ( 0.26 - 0.69 ) | -2.33(-2.44 to -2.23) |
| Saint Lucia | 1.07 ( 0.69 - 1.62 ) | 1.23 ( 0.8 - 1.85 ) | 1.4 ( 0.86 - 2.19 ) | 0.58 ( 0.36 - 0.92 ) | -2.6(-2.71 to -2.49) |
| Saint Vincent and the Grenadines | 0.63 ( 0.4 - 0.97 ) | 0.89 ( 0.56 - 1.37 ) | 0.93 ( 0.57 - 1.43 ) | 0.65 ( 0.4 - 1.01 ) | -1.08(-1.15 to -1.01) |
| Samoa | 5.31 ( 3.63 - 7.43 ) | 6.05 ( 4.13 - 8.46 ) | 6.41 ( 4.3 - 9.49 ) | 4.38 ( 2.93 - 6.49 ) | -1.14(-1.21 to -1.06) |
| San Marino | 0.4 ( 0.25 - 0.6 ) | 1.22 ( 0.77 - 1.83 ) | 0.62 ( 0.38 - 0.99 ) | 0.92 ( 0.56 - 1.46 ) | -0.96(-1.03 to -0.9) |
| Sao Tome and Principe | 0.78 ( 0.48 - 1.22 ) | 1.17 ( 0.73 - 1.84 ) | 0.89 ( 0.57 - 1.35 ) | 0.74 ( 0.47 - 1.14 ) | -1.9(-2.18 to -1.63) |
| Saudi Arabia | 562.83 ( 361.1 - 841.28 ) | 9.1 ( 5.82 - 13.62 ) | 999.36 ( 635.04 - 1465.1 ) | 4.5 ( 2.81 - 6.68 ) | -2.3(-2.43 to -2.16) |
| Senegal | 47.29 ( 31.13 - 67.41 ) | 1.35 ( 0.88 - 1.93 ) | 57.06 ( 36.99 - 87.6 ) | 0.69 ( 0.43 - 1.08 ) | -2.07(-2.16 to -1.98) |
| Serbia | 131.52 ( 86.81 - 193.18 ) | 1.15 ( 0.76 - 1.68 ) | 132.57 ( 85.03 - 197.4 ) | 0.87 ( 0.56 - 1.29 ) | -1.1(-1.48 to -0.72) |
| Seychelles | 2.71 ( 1.83 - 3.79 ) | 4.8 ( 3.26 - 6.73 ) | 3.46 ( 2.32 - 5.03 ) | 3.06 ( 2.06 - 4.5 ) | -1.56(-1.66 to -1.47) |
| Sierra Leone | 17.55 ( 11.47 - 24.98 ) | 0.87 ( 0.57 - 1.23 ) | 20.56 ( 13.78 - 29.81 ) | 0.51 ( 0.34 - 0.74 ) | -1.77(-1.98 to -1.56) |
| Singapore | 19.53 ( 13.01 - 28.15 ) | 0.81 ( 0.55 - 1.17 ) | 38.88 ( 25.47 - 57.43 ) | 0.45 ( 0.29 - 0.66 ) | -1.92(-2.07 to -1.78) |
| Slovakia | 52.26 ( 33.95 - 77.22 ) | 0.89 ( 0.58 - 1.31 ) | 62.06 ( 40.54 - 93.49 ) | 0.68 ( 0.44 - 1.02 ) | -0.76(-0.98 to -0.55) |
| Slovenia | 25.13 ( 16.11 - 38.39 ) | 1.03 ( 0.66 - 1.58 ) | 33.01 ( 20.42 - 51.36 ) | 0.84 ( 0.53 - 1.31 ) | -0.36(-0.49 to -0.23) |
| Solomon Islands | 10.07 ( 6.91 - 14.34 ) | 7.05 ( 4.85 - 10.01 ) | 18.86 ( 13.08 - 26.5 ) | 5.23 ( 3.65 - 7.4 ) | -0.73(-0.86 to -0.61) |
| Somalia | 43.54 ( 26.27 - 66.41 ) | 1.61 ( 0.98 - 2.41 ) | 75.46 ( 44.53 - 118.12 ) | 1.07 ( 0.65 - 1.63 ) | -1.41(-1.57 to -1.26) |
| South Africa | 1110.67 ( 762.87 - 1611.08 ) | 5.29 ( 3.61 - 7.58 ) | 715.48 ( 488.7 - 990.1 ) | 1.48 ( 1.01 - 2.05 ) | -4.5(-4.78 to -4.22) |
| South Sudan | 111.66 ( 66.98 - 167.1 ) | 4.48 ( 2.69 - 6.67 ) | 84.02 ( 53.99 - 128.61 ) | 2.37 ( 1.49 - 3.74 ) | -2.17(-2.26 to -2.08) |
| Spain | 1681.4 ( 1138.44 - 2407.14 ) | 3.34 ( 2.28 - 4.79 ) | 1569.09 ( 1039.56 - 2354.09 ) | 1.84 ( 1.22 - 2.71 ) | -1.91(-1.96 to -1.86) |
| Sri Lanka | 384.88 ( 260.1 - 548.85 ) | 3.92 ( 2.67 - 5.61 ) | 392.22 ( 265.88 - 580.66 ) | 1.48 ( 1 - 2.21 ) | -3.31(-3.49 to -3.14) |
| Sudan | 462.57 ( 287.78 - 683.5 ) | 5.22 ( 3.22 - 7.8 ) | 493.89 ( 313.78 - 737.02 ) | 2.72 ( 1.71 - 4.1 ) | -2.08(-2.2 to -1.96) |
| Suriname | 8.56 ( 5.69 - 12.26 ) | 3.29 ( 2.21 - 4.74 ) | 9.89 ( 6.41 - 14.91 ) | 1.55 ( 1 - 2.34 ) | -2.85(-3.01 to -2.68) |
| Sweden | 111.88 ( 74.65 - 165.13 ) | 0.78 ( 0.53 - 1.16 ) | 149.88 ( 93.11 - 238.74 ) | 0.74 ( 0.46 - 1.14 ) | -0.15(-0.27 to -0.03) |
| Switzerland | 130.04 ( 88.7 - 188.25 ) | 1.31 ( 0.89 - 1.89 ) | 149.58 ( 98.1 - 220.35 ) | 0.87 ( 0.58 - 1.29 ) | -1.26(-1.34 to -1.17) |
| Syrian Arab Republic | 461.52 ( 316.9 - 637.67 ) | 9.35 ( 6.4 - 13.06 ) | 522.39 ( 356.57 - 754.59 ) | 4.1 ( 2.82 - 5.84 ) | -2.8(-2.93 to -2.67) |
| Taiwan | 56.39 ( 33.95 - 90.25 ) | 0.39 ( 0.24 - 0.62 ) | 103.17 ( 58.76 - 162.56 ) | 0.24 ( 0.14 - 0.38 ) | -1.71(-1.79 to -1.64) |
| Tajikistan | 102.67 ( 68.84 - 146 ) | 3.77 ( 2.55 - 5.37 ) | 108.64 ( 67.51 - 160.4 ) | 1.85 ( 1.14 - 2.8 ) | -2.18(-2.29 to -2.06) |
| Thailand | 2162.06 ( 1540.29 - 2996.73 ) | 6.44 ( 4.59 - 8.9 ) | 3166.46 ( 2133.03 - 4422.68 ) | 2.91 ( 1.96 - 4.06 ) | -2.71(-2.85 to -2.56) |
| Timor-Leste | 16.29 ( 10.48 - 24.02 ) | 6.46 ( 4.07 - 9.31 ) | 25.26 ( 16.71 - 37.42 ) | 3.15 ( 2.08 - 4.69 ) | -2.28(-2.35 to -2.2) |
| Togo | 31.58 ( 21.03 - 46.18 ) | 2.74 ( 1.83 - 3.98 ) | 75.41 ( 47.54 - 116.31 ) | 1.91 ( 1.22 - 2.93 ) | -1.27(-1.32 to -1.22) |
| Tokelau | 0.06 ( 0.04 - 0.09 ) | 4.61 ( 2.95 - 6.91 ) | 0.04 ( 0.03 - 0.06 ) | 2.83 ( 1.89 - 4.19 ) | -1.7(-1.76 to -1.64) |
| Tonga | 1.96 ( 1.28 - 2.79 ) | 3.64 ( 2.37 - 5.19 ) | 1.74 ( 1.13 - 2.56 ) | 2.21 ( 1.44 - 3.26 ) | -1.61(-1.64 to -1.58) |
| Trinidad and Tobago | 10.67 ( 7.14 - 15.88 ) | 1.26 ( 0.84 - 1.87 ) | 12.31 ( 7.83 - 18.79 ) | 0.64 ( 0.4 - 0.97 ) | -2.49(-2.64 to -2.33) |
| Tunisia | 411.73 ( 269.92 - 571.54 ) | 8.66 ( 5.67 - 12.04 ) | 502.41 ( 346.78 - 705.22 ) | 3.84 ( 2.66 - 5.39 ) | -2.66(-2.77 to -2.56) |
| Türkiye | 2062.15 ( 1420.19 - 2931.74 ) | 6 ( 4.12 - 8.47 ) | 2234.37 ( 1463.71 - 3171.48 ) | 2.36 ( 1.54 - 3.32 ) | -1.91(-2.05 to -1.76) |
| Turkmenistan | 123.19 ( 82.46 - 176.7 ) | 6.22 ( 4.19 - 8.97 ) | 154.42 ( 99.55 - 230.57 ) | 3.63 ( 2.34 - 5.37 ) | -1.28(-1.3 to -1.25) |
| Tuvalu | 0.36 ( 0.24 - 0.52 ) | 5.08 ( 3.35 - 7.16 ) | 0.37 ( 0.24 - 0.56 ) | 3.43 ( 2.23 - 5.13 ) | -3.33(-3.49 to -3.16) |
| Uganda | 43.5 ( 29.51 - 61.26 ) | 0.72 ( 0.48 - 1.03 ) | 78.76 ( 50.11 - 116.86 ) | 0.55 ( 0.35 - 0.81 ) | -1.04(-1.26 to -0.82) |
| Ukraine | 966.21 ( 666.2 - 1392.23 ) | 1.38 ( 0.94 - 1.96 ) | 767.05 ( 529.11 - 1093.65 ) | 1.04 ( 0.72 - 1.48 ) | -0.94(-1.18 to -0.71) |
| United Arab Emirates | 22.53 ( 15.24 - 32.07 ) | 4.44 ( 2.97 - 6.43 ) | 163.25 ( 102.82 - 246.12 ) | 3.85 ( 2.44 - 5.82 ) | -0.45(-0.66 to -0.23) |
| United Kingdom | 1510.26 ( 1004.72 - 2191.63 ) | 1.76 ( 1.17 - 2.56 ) | 1063.81 ( 677.14 - 1639.88 ) | 0.85 ( 0.55 - 1.3 ) | -2.71(-2.88 to -2.53) |
| United Republic of Tanzania | 333.61 ( 225.89 - 484.17 ) | 3.18 ( 2.14 - 4.57 ) | 531.57 ( 330.01 - 799.92 ) | 2 ( 1.23 - 3.07 ) | -1.46(-1.58 to -1.34) |
| United States of America | 4241.18 ( 2885.3 - 6183.26 ) | 1.37 ( 0.94 - 1.99 ) | 4467.53 ( 2955.29 - 6804.08 ) | 0.81 ( 0.54 - 1.21 ) | -1.88(-2.06 to -1.7) |
| United States Virgin Islands | 0.84 ( 0.49 - 1.39 ) | 0.98 ( 0.55 - 1.64 ) | 1.31 ( 0.74 - 2.22 ) | 0.71 ( 0.41 - 1.18 ) | -1.03(-1.08 to -0.98) |
| Uruguay | 21.95 ( 14.39 - 32.14 ) | 0.59 ( 0.39 - 0.87 ) | 24.67 ( 15.46 - 37.84 ) | 0.5 ( 0.32 - 0.75 ) | -0.65(-0.74 to -0.57) |
| Uzbekistan | 147.47 ( 97.36 - 215.92 ) | 1.26 ( 0.83 - 1.84 ) | 367.4 ( 244.09 - 521.05 ) | 1.34 ( 0.89 - 1.94 ) | 0.12(-0.22 to 0.46) |
| Vanuatu | 1.6 ( 1.11 - 2.26 ) | 2.54 ( 1.71 - 3.59 ) | 2.65 ( 1.76 - 3.77 ) | 1.53 ( 1.03 - 2.21 ) | -1.83(-1.98 to -1.68) |
| Venezuela (Bolivarian Republic of) | 239.49 ( 153.8 - 344.23 ) | 2.49 ( 1.59 - 3.6 ) | 289.52 ( 185.06 - 445.62 ) | 0.96 ( 0.61 - 1.47 ) | -3.02(-3.17 to -2.86) |
| Viet Nam | 1933.27 ( 1336.33 - 2736.35 ) | 4.98 ( 3.46 - 7.01 ) | 2705.78 ( 1877.98 - 3844.12 ) | 2.81 ( 1.97 - 4 ) | -2.22(-2.34 to -2.09) |
| Yemen | 267.95 ( 176.18 - 375.68 ) | 5.54 ( 3.67 - 7.8 ) | 679.11 ( 456.16 - 965.61 ) | 4.93 ( 3.27 - 6.96 ) | -0.8(-1.05 to -0.55) |
| Zambia | 67.66 ( 46.34 - 95.37 ) | 2.53 ( 1.7 - 3.61 ) | 85.09 ( 56.92 - 121.67 ) | 1.3 ( 0.86 - 1.85 ) | -2.4(-2.52 to -2.28) |
| Zimbabwe | 187.22 ( 125.18 - 271.55 ) | 4.83 ( 3.16 - 6.94 ) | 190.31 ( 121.58 - 281.07 ) | 2.91 ( 1.85 - 4.38 ) | -1.71(-1.8 to -1.61) |

**Table S3.** Changes in YLDs of cataract attributable to HAP according to decomposition analysis from 1990 to 2021 at the global, SDI, and 21 GBD regional levels.

| **Location** | **Overall** | **Aging** | **Population growth** | **Epidemiological shifts** | **Aging (%)** | **Population growth (%)** | **Epidemiological shifts (%)** |
| --- | --- | --- | --- | --- | --- | --- | --- |
| **Global** | 542294.7 | 476104.7 | 927402.21 | -861212.2 | 87.79 | 171.01 | -158.81 |
| **SDI Regions** |  |  |  |  |  |  |  |
| High SDI | -6110.6 | 5022.79 | 4753.07 | -15886.46 | -82.2 | -77.78 | 259.98 |
| High-middle SDI | 21122.38 | 55791.56 | 53935.35 | -88604.53 | 264.13 | 255.35 | -419.48 |
| Middle SDI | 119198.56 | 251996.25 | 307220.54 | -440018.23 | 211.41 | 257.74 | -369.15 |
| Low-middle SDI | 266752.89 | 162236.16 | 565334.29 | -460817.56 | 60.82 | 211.93 | -172.75 |
| Low SDI | 141213.62 | -10596.44 | 235705.42 | -83895.36 | -7.5 | 166.91 | -59.41 |
| **GBD Regions** |  |  |  |  |  |  |  |
| East Asia | 112086.9 | 199749.58 | 120922.55 | -208585.22 | 178.21 | 107.88 | -186.09 |
| Southeast Asia | 40210.73 | 77537.21 | 152974.39 | -190300.87 | 192.83 | 380.43 | -473.26 |
| Central Asia | -3015.42 | 281.48 | 4473.11 | -7770 | -9.33 | -148.34 | 257.68 |
| Oceania | 2575.63 | 352.53 | 3135.98 | -912.88 | 13.69 | 121.76 | -35.44 |
| Central Europe | -1261.9 | 3802.61 | 587.45 | -5651.96 | -301.34 | -46.55 | 447.89 |
| High-income Asia Pacific | -642.49 | 744.33 | 228.08 | -1614.91 | -115.85 | -35.5 | 251.35 |
| Eastern Europe | -5746.86 | 2363.56 | 67.91 | -8178.33 | -41.13 | -1.18 | 142.31 |
| Western Europe | -3136.57 | 1266.73 | 696.36 | -5099.66 | -40.39 | -22.2 | 162.59 |
| Australasia | -156.27 | 84.54 | 111.71 | -352.53 | -54.1 | -71.48 | 225.58 |
| Southern Latin America | -1243.48 | 643.08 | 1251.9 | -3138.46 | -51.72 | -100.68 | 252.39 |
| Caribbean | -264.47 | 937.26 | 1489.6 | -2691.33 | -354.4 | -563.25 | 1017.65 |
| High-income North America | 22.14 | 120.87 | 143.59 | -242.32 | 545.89 | 648.53 | -1094.42 |
| Andean Latin America | -910.16 | 4065.77 | 10301.56 | -15277.49 | -446.71 | -1131.84 | 1678.55 |
| Central Latin America | 4304.31 | 10639.2 | 16607.36 | -22942.25 | 247.18 | 385.83 | -533.01 |
| North Africa and Middle East | -19284.17 | 6264.27 | 35103.57 | -60652.01 | -32.48 | -182.03 | 314.52 |
| Tropical Latin America | -6304.13 | 12262.02 | 16586.96 | -35153.11 | -194.51 | -263.11 | 557.62 |
| South Asia | 322131.67 | 249514.09 | 682572.34 | -609954.76 | 77.46 | 211.89 | -189.35 |
| Central Sub-Saharan Africa | 2010.93 | -166.04 | 4220.34 | -2043.37 | -8.26 | 209.87 | -101.61 |
| Southern Sub-Saharan Africa | -2336.81 | 1294.04 | 7530.19 | -11161.04 | -55.38 | -322.24 | 477.62 |
| Western Sub-Saharan Africa | 56974.59 | -14989.8 | 94970.59 | -23006.21 | -26.31 | 166.69 | -40.38 |
| Eastern Sub-Saharan AfricaBoth | 46280.54 | -4695.5 | 70893.48 | -19917.44 | -10.15 | 153.18 | -43.04 |

**Table S4.** Changes in YLDs of cataract attributable to smoking according to decomposition analysis from 1990 to 2021 at the global, SDI, and 21 GBD regional levels.

| **Location** | **Overall** | **Aging** | **Population growth** | **Epidemiological shifts** | **Aging (%)** | **Population growth (%)** | **Epidemiological shifts (%)** |
| --- | --- | --- | --- | --- | --- | --- | --- |
| **Global** | 63091.26 | 29214.98 | 127740.68 | -93864.39 | 46.31 | 202.47 | -148.78 |
| **SDI Regions** |  |  |  |  |  |  |  |
| High SDI | 1901.07 | 3147.26 | 6681.76 | -7927.95 | 165.55 | 351.47 | -417.03 |
| High-middle SDI | 17035.99 | 6292.05 | 18210.22 | -7466.28 | 36.93 | 106.89 | -43.83 |
| Middle SDI | 26376.41 | 16019.1 | 51048.78 | -40691.47 | 60.73 | 193.54 | -154.27 |
| Low-middle SDI | 14770.38 | 7673.02 | 53808.63 | -46711.27 | 51.95 | 364.3 | -316.25 |
| Low SDI | 2987.06 | -557.66 | 10857.12 | -7312.39 | -18.67 | 363.47 | -244.8 |
| **GBD Regions** |  |  |  |  |  |  |  |
| Oceania | 185.15 | 4.34 | 256.49 | -75.68 | 2.35 | 138.53 | -40.88 |
| Southeast Asia | 8235.36 | 4208.57 | 19764.29 | -15737.5 | 51.1 | 239.99 | -191.1 |
| East Asia | 28387.38 | 14933.81 | 28904.64 | -15451.07 | 52.61 | 101.82 | -54.43 |
| High-income Asia Pacific | 162.18 | 974.52 | 941.32 | -1753.67 | 600.9 | 580.43 | -1081.33 |
| Eastern Europe | 395.48 | 297.83 | 311.59 | -213.95 | 75.31 | 78.79 | -54.1 |
| Central Asia | 620.66 | -24.05 | 872.62 | -227.91 | -3.87 | 140.59 | -36.72 |
| Central Europe | -236.46 | 342.04 | 267.58 | -846.07 | -144.65 | -113.16 | 357.81 |
| Australasia | 67.01 | 49.29 | 164.47 | -146.76 | 73.56 | 245.44 | -219 |
| Western Europe | -1010.01 | 1436.3 | 2670.21 | -5116.52 | -142.21 | -264.37 | 506.58 |
| Andean Latin America | 413.75 | 152.59 | 762.43 | -501.27 | 36.88 | 184.27 | -121.15 |
| Southern Latin America | 87.44 | 58.44 | 445.1 | -416.1 | 66.84 | 509.02 | -475.85 |
| High-income North America | 330.73 | 908.31 | 2043.01 | -2620.59 | 274.64 | 617.73 | -792.37 |
| Central Latin America | 236.39 | 795.71 | 2703.31 | -3262.63 | 336.61 | 1143.57 | -1380.18 |
| Caribbean | 56.85 | 107.9 | 447.97 | -499.02 | 189.81 | 788.01 | -877.82 |
| North Africa and Middle East | 5578.26 | 206.91 | 13261.75 | -7890.4 | 3.71 | 237.74 | -141.45 |
| Tropical Latin America | 951.17 | 1688.03 | 5356.41 | -6093.26 | 177.47 | 563.14 | -640.61 |
| Central Sub-Saharan Africa | 84.18 | -10.82 | 164.32 | -69.32 | -12.86 | 195.21 | -82.35 |
| Southern Sub-Saharan Africa | -384.95 | 14.07 | 1049.83 | -1448.85 | -3.66 | -272.72 | 376.38 |
| South Asia | 17108.46 | 13277.54 | 63174.58 | -59343.66 | 77.61 | 369.26 | -346.87 |
| Western Sub-Saharan Africa | 1211.67 | -215.76 | 1828.91 | -401.48 | -17.81 | 150.94 | -33.13 |
| Eastern Sub-Saharan Africa | 610.55 | -149.68 | 2053.79 | -1293.57 | -24.52 | 336.38 | -211.87 |

**Table S5.** The effective difference in ASYR among countries and territories in 2021 for cataract attributable to HAP according to frontier analysis.

| **Location** | **ASYR,**  **per 100,000** | **SDI level** | **Efficiency difference** |
| --- | --- | --- | --- |
| Pakistan | 122.45 | 0.5040 | 118.89 |
| Ethiopia | 116.12 | 0.3588 | 107.74 |
| South Sudan | 105.01 | 0.2784 | 96.16 |
| Myanmar | 96.65 | 0.5339 | 96.04 |
| Cambodia | 98.25 | 0.4736 | 92.48 |
| Nigeria | 92.54 | 0.5034 | 88.93 |
| Papua New Guinea | 95.29 | 0.4178 | 86.92 |
| Afghanistan | 90.17 | 0.3372 | 81.80 |
| Mali | 85.94 | 0.2686 | 76.85 |
| Bangladesh | 80.97 | 0.4924 | 76.42 |
| India | 71.11 | 0.5754 | 70.75 |
| Gambia | 75.53 | 0.4097 | 67.16 |
| Kenya | 63.37 | 0.5238 | 62.68 |
| Benin | 65.93 | 0.3735 | 57.57 |
| Solomon Islands | 64.59 | 0.4294 | 56.22 |
| Guinea | 61.01 | 0.3364 | 52.65 |
| Togo | 54.68 | 0.4085 | 46.31 |
| Burkina Faso | 54.63 | 0.2851 | 46.01 |
| Philippines | 45.19 | 0.6512 | 45.04 |
| Sri Lanka | 44.95 | 0.7015 | 44.89 |
| Kiribati | 45.17 | 0.5272 | 44.49 |
| Samoa | 44.34 | 0.5934 | 44.08 |
| Indonesia | 43.15 | 0.6569 | 43.03 |
| Chad | 59.07 | 0.2404 | 42.69 |
| Timor-Leste | 51.03 | 0.4447 | 42.66 |
| Cote d'Ivoire | 50.88 | 0.4259 | 42.52 |
| Mozambique | 50.41 | 0.3265 | 42.05 |
| United Republic of Tanzania | 49.90 | 0.4466 | 41.92 |
| Liberia | 49.68 | 0.3524 | 41.32 |
| Ghana | 41.35 | 0.5649 | 40.92 |
| Lesotho | 41.65 | 0.5104 | 40.69 |
| Zimbabwe | 45.96 | 0.4738 | 40.19 |
| Vanuatu | 44.26 | 0.4731 | 38.31 |
| Fiji | 36.52 | 0.6751 | 36.43 |
| Nepal | 44.71 | 0.4332 | 36.35 |
| Zambia | 38.47 | 0.5059 | 36.10 |
| Eswatini | 36.23 | 0.5855 | 35.93 |
| Eritrea | 43.91 | 0.4039 | 35.55 |
| Mauritania | 39.14 | 0.4989 | 35.12 |
| Comoros | 40.80 | 0.4760 | 34.74 |
| Malawi | 41.75 | 0.3846 | 33.38 |
| Sao Tome and Principe | 35.24 | 0.5054 | 32.98 |
| Namibia | 32.62 | 0.6176 | 32.42 |
| Micronesia (Federated States of) | 31.87 | 0.5875 | 31.58 |
| Guinea-Bissau | 39.60 | 0.3531 | 31.23 |
| Sudan | 31.32 | 0.5419 | 30.74 |
| Niger | 72.00 | 0.1681 | 30.62 |
| Viet Nam | 30.69 | 0.6279 | 30.51 |
| Marshall Islands | 29.81 | 0.5741 | 29.45 |
| Equatorial Guinea | 28.94 | 0.6579 | 28.82 |
| Cameroon | 33.92 | 0.4797 | 28.69 |
| Guatemala | 29.27 | 0.5400 | 28.68 |
| Senegal | 35.70 | 0.4081 | 27.34 |
| Botswana | 24.68 | 0.6427 | 24.52 |
| Nicaragua | 24.35 | 0.5240 | 23.62 |
| Thailand | 23.35 | 0.6825 | 23.28 |
| Peru | 23.11 | 0.6621 | 23.00 |
| Sierra Leone | 30.63 | 0.3587 | 22.27 |
| Yemen | 30.05 | 0.4504 | 22.03 |
| Bolivia (Plurinational State of) | 20.94 | 0.5990 | 20.71 |
| Paraguay | 20.45 | 0.6357 | 20.29 |
| Tonga | 20.28 | 0.6263 | 20.09 |
| Haiti | 27.66 | 0.4483 | 19.68 |
| Tajikistan | 19.49 | 0.5415 | 18.91 |
| Cabo Verde | 19.22 | 0.5335 | 18.61 |
| Uganda | 26.84 | 0.4233 | 18.48 |
| China | 16.98 | 0.7216 | 16.93 |
| Mongolia | 17.09 | 0.6176 | 16.89 |
| Lao People's Democratic Republic | 21.47 | 0.4891 | 16.80 |
| Congo | 16.74 | 0.5831 | 16.44 |
| Kyrgyzstan | 16.36 | 0.6040 | 16.12 |
| Madagascar | 24.48 | 0.4002 | 16.12 |
| Honduras | 15.82 | 0.5130 | 14.89 |
| Djibouti | 19.25 | 0.4880 | 14.62 |
| Georgia | 14.59 | 0.7325 | 14.55 |
| American Samoa | 14.04 | 0.7237 | 13.99 |
| Cook Islands | 13.24 | 0.7791 | 13.21 |
| Tuvalu | 13.53 | 0.5766 | 13.18 |
| Northern Mariana Islands | 13.03 | 0.7715 | 12.99 |
| South Africa | 12.87 | 0.6796 | 12.79 |
| Panama | 12.68 | 0.7089 | 12.63 |
| Central African Republic | 20.90 | 0.3092 | 12.54 |
| Guam | 11.86 | 0.8040 | 11.83 |
| Mexico | 10.04 | 0.6646 | 9.93 |
| Uzbekistan | 9.78 | 0.6626 | 9.68 |
| El Salvador | 9.96 | 0.5638 | 9.54 |
| Angola | 17.31 | 0.4537 | 9.35 |
| Democratic People's Republic of Korea | 9.18 | 0.5699 | 8.79 |
| Niue | 8.81 | 0.7262 | 8.76 |
| Burundi | 16.82 | 0.2894 | 8.27 |
| Rwanda | 16.40 | 0.4356 | 8.02 |
| Nauru | 7.91 | 0.6252 | 7.73 |
| Suriname | 7.70 | 0.6337 | 7.53 |
| Kazakhstan | 7.31 | 0.7251 | 7.27 |
| Azerbaijan | 7.15 | 0.6949 | 7.09 |
| Colombia | 7.17 | 0.6554 | 7.05 |
| Hungary | 6.76 | 0.7908 | 6.73 |
| Bosnia and Herzegovina | 6.74 | 0.7231 | 6.69 |
| Montenegro | 6.58 | 0.7958 | 6.55 |
| Brazil | 6.46 | 0.6530 | 6.31 |
| Belize | 6.37 | 0.6102 | 6.15 |
| Costa Rica | 6.17 | 0.7003 | 6.10 |
| Republic of Moldova | 6.01 | 0.7322 | 5.97 |
| Maldives | 5.84 | 0.6509 | 5.69 |
| Latvia | 5.55 | 0.8307 | 5.52 |
| Bhutan | 11.45 | 0.4731 | 5.44 |
| Ecuador | 5.47 | 0.6610 | 5.36 |
| Albania | 5.24 | 0.7068 | 5.19 |
| Jamaica | 5.16 | 0.6833 | 5.09 |
| Gabon | 5.26 | 0.6347 | 5.09 |
| Serbia | 4.99 | 0.7924 | 4.96 |
| Dominican Republic | 4.85 | 0.6194 | 4.66 |
| North Macedonia | 4.54 | 0.7506 | 4.51 |
| Morocco | 4.41 | 0.5627 | 3.98 |
| Dominica | 3.88 | 0.7470 | 3.84 |
| Chile | 3.62 | 0.7715 | 3.58 |
| Poland | 3.45 | 0.8120 | 3.42 |
| Romania | 3.43 | 0.7685 | 3.40 |
| Guyana | 3.41 | 0.6508 | 3.26 |
| Estonia | 3.08 | 0.8449 | 3.05 |
| Armenia | 3.11 | 0.7018 | 3.05 |
| Malaysia | 2.64 | 0.7425 | 2.60 |
| Bulgaria | 2.49 | 0.7682 | 2.46 |
| Saint Lucia | 2.36 | 0.6725 | 2.26 |
| Mauritius | 2.27 | 0.7183 | 2.22 |
| Slovenia | 2.21 | 0.8424 | 2.18 |
| Palestine | 2.31 | 0.6310 | 2.14 |
| Türkiye | 2.07 | 0.7127 | 2.02 |
| Ukraine | 1.96 | 0.7608 | 1.93 |
| Saint Vincent and the Grenadines | 1.99 | 0.6372 | 1.83 |
| Taiwan (Province of China) | 1.75 | 0.8747 | 1.73 |
| Lithuania | 1.75 | 0.8565 | 1.73 |
| Oman | 1.71 | 0.7734 | 1.68 |
| Croatia | 1.54 | 0.7983 | 1.51 |
| Saudi Arabia | 1.52 | 0.8151 | 1.49 |
| Iraq | 1.41 | 0.6626 | 1.31 |
| Grenada | 1.37 | 0.6690 | 1.28 |
| Saint Kitts and Nevis | 1.26 | 0.7550 | 1.23 |
| Cuba | 1.33 | 0.6687 | 1.23 |
| Argentina | 1.26 | 0.7231 | 1.21 |
| Bermuda | 1.16 | 0.8214 | 1.14 |
| Seychelles | 1.12 | 0.7302 | 1.07 |
| Bahrain | 1.03 | 0.7530 | 0.99 |
| Libya | 0.94 | 0.7258 | 0.89 |
| Russian Federation | 0.89 | 0.8085 | 0.86 |
| Bahamas | 0.86 | 0.8050 | 0.82 |
| Spain | 0.73 | 0.7693 | 0.69 |
| Brunei Darussalam | 0.71 | 0.8102 | 0.68 |
| Democratic Republic of the Congo | 9.04 | 0.3832 | 0.67 |
| Antigua and Barbuda | 0.70 | 0.7499 | 0.67 |
| Venezuela (Bolivarian Republic of) | 0.90 | 0.5965 | 0.64 |
| Lebanon | 0.63 | 0.7447 | 0.60 |
| Uruguay | 0.59 | 0.7193 | 0.54 |
| Belarus | 0.51 | 0.7845 | 0.47 |
| Czechia | 0.45 | 0.8285 | 0.42 |
| United States Virgin Islands | 0.44 | 0.8218 | 0.41 |
| Algeria | 0.51 | 0.6595 | 0.40 |
| Slovakia | 0.37 | 0.8106 | 0.34 |
| Kuwait | 0.35 | 0.8467 | 0.32 |
| Tunisia | 0.38 | 0.6824 | 0.31 |
| Iran (Islamic Republic of) | 0.32 | 0.6972 | 0.26 |
| Italy | 0.29 | 0.8058 | 0.25 |
| Turkmenistan | 0.31 | 0.6822 | 0.24 |
| Portugal | 0.25 | 0.7442 | 0.22 |
| Greece | 0.23 | 0.7919 | 0.20 |
| Singapore | 0.21 | 0.8561 | 0.19 |
| Australia | 0.17 | 0.8443 | 0.14 |
| Malta | 0.14 | 0.8016 | 0.11 |
| Palau | 0.13 | 0.7540 | 0.10 |
| New Zealand | 0.12 | 0.8494 | 0.09 |
| Egypt | 0.31 | 0.6068 | 0.09 |
| Austria | 0.10 | 0.8538 | 0.07 |
| Greenland | 0.09 | 0.8262 | 0.07 |
| Japan | 0.09 | 0.8712 | 0.06 |
| Israel | 0.09 | 0.8090 | 0.06 |
| Trinidad and Tobago | 0.08 | 0.7688 | 0.05 |
| France | 0.08 | 0.8384 | 0.05 |
| Ireland | 0.07 | 0.8738 | 0.05 |
| Luxembourg | 0.07 | 0.8844 | 0.05 |
| Republic of Korea | 0.06 | 0.8867 | 0.04 |
| United States of America | 0.06 | 0.8624 | 0.04 |
| Cyprus | 0.07 | 0.8356 | 0.04 |
| Belgium | 0.06 | 0.8537 | 0.03 |
| Tokelau | 0.10 | 0.6864 | 0.03 |
| San Marino | 0.06 | 0.8880 | 0.03 |
| Qatar | 0.06 | 0.8469 | 0.03 |
| Andorra | 0.05 | 0.8694 | 0.03 |
| Finland | 0.05 | 0.8598 | 0.03 |
| Iceland | 0.05 | 0.8764 | 0.03 |
| Syrian Arab Republic | 0.20 | 0.6230 | 0.02 |
| Denmark | 0.05 | 0.8964 | 0.02 |
| Jordan | 0.06 | 0.7253 | 0.01 |
| Canada | 0.04 | 0.8732 | 0.01 |
| Netherlands | 0.04 | 0.8885 | 0.01 |
| United Arab Emirates | 0.04 | 0.8493 | 0.01 |
| Monaco | 0.03 | 0.9083 | 0.01 |
| Puerto Rico | 0.04 | 0.8255 | 0.01 |
| Germany | 0.03 | 0.9030 | 0.01 |
| Norway | 0.03 | 0.9161 | 0.01 |
| Sweden | 0.03 | 0.8869 | 0.0004 |
| Switzerland | 0.03 | 0.9331 | 0.0004 |
| Barbados | 0.03 | 0.7467 | 0.0004 |
| United Kingdom | 0.02 | 0.8590 | 0 |
| Somalia | 47.08 | 0.0777 | 0 |

**Table S6.** The effective difference in ASYR among countries and territories in 2021 for cataract attributable to smoking according to frontier analysis.

| **Location** | **ASYR,**  **per 100,000** | **SDI level** | **Efficiency difference** |
| --- | --- | --- | --- |
| Pakistan | 9.65 | 0.5040 | 9.55 |
| Cambodia | 8.84 | 0.4736 | 8.75 |
| Lebanon | 8.27 | 0.7447 | 8.17 |
| Kiribati | 7.08 | 0.5272 | 6.98 |
| Bangladesh | 6.09 | 0.4924 | 6.00 |
| Palestine | 5.73 | 0.6310 | 5.63 |
| Indonesia | 5.72 | 0.6569 | 5.62 |
| Solomon Islands | 5.23 | 0.4294 | 5.13 |
| India | 5.21 | 0.5754 | 5.12 |
| Myanmar | 5.18 | 0.5339 | 5.09 |
| Iraq | 5.07 | 0.6626 | 4.97 |
| Egypt | 4.97 | 0.6068 | 4.87 |
| Yemen | 4.93 | 0.4504 | 4.83 |
| Papua New Guinea | 4.85 | 0.4178 | 4.75 |
| Algeria | 4.70 | 0.6595 | 4.60 |
| Saudi Arabia | 4.50 | 0.8151 | 4.40 |
| Samoa | 4.38 | 0.5934 | 4.28 |
| Philippines | 4.18 | 0.6512 | 4.08 |
| Syrian Arab Republic | 4.10 | 0.6230 | 4.00 |
| Libya | 4.00 | 0.7258 | 3.90 |
| Kuwait | 3.97 | 0.8467 | 3.88 |
| Nepal | 3.90 | 0.4332 | 3.80 |
| Micronesia (Federated States of) | 3.89 | 0.5875 | 3.79 |
| United Arab Emirates | 3.85 | 0.8493 | 3.75 |
| Tunisia | 3.84 | 0.6824 | 3.74 |
| Malaysia | 3.73 | 0.7425 | 3.63 |
| Bahrain | 3.72 | 0.7530 | 3.62 |
| Oman | 3.70 | 0.7734 | 3.60 |
| Turkmenistan | 3.63 | 0.6822 | 3.53 |
| Kyrgyzstan | 3.44 | 0.6040 | 3.34 |
| Tuvalu | 3.43 | 0.5766 | 3.33 |
| Lesotho | 3.41 | 0.5104 | 3.31 |
| Nauru | 3.40 | 0.6252 | 3.31 |
| Afghanistan | 3.39 | 0.3372 | 3.29 |
| Armenia | 3.26 | 0.7018 | 3.16 |
| Azerbaijan | 3.16 | 0.6949 | 3.06 |
| Timor-Leste | 3.15 | 0.4447 | 3.06 |
| Fiji | 3.13 | 0.6751 | 3.03 |
| Republic of Moldova | 3.06 | 0.7322 | 2.97 |
| Seychelles | 3.06 | 0.7302 | 2.96 |
| Cook Islands | 3.06 | 0.7791 | 2.96 |
| Georgia | 3.04 | 0.7325 | 2.94 |
| Thailand | 2.91 | 0.6825 | 2.81 |
| Zimbabwe | 2.91 | 0.4738 | 2.81 |
| Tokelau | 2.83 | 0.6864 | 2.73 |
| China | 2.83 | 0.7216 | 2.73 |
| Viet Nam | 2.81 | 0.6279 | 2.71 |
| Mauritius | 2.73 | 0.7183 | 2.63 |
| Sudan | 2.72 | 0.5419 | 2.62 |
| American Samoa | 2.66 | 0.7237 | 2.56 |
| Paraguay | 2.64 | 0.6357 | 2.54 |
| Northern Mariana Islands | 2.58 | 0.7715 | 2.48 |
| Iran (Islamic Republic of) | 2.55 | 0.6972 | 2.45 |
| Qatar | 2.51 | 0.8469 | 2.42 |
| Brazil | 2.50 | 0.6530 | 2.40 |
| Guam | 2.49 | 0.8040 | 2.40 |
| Marshall Islands | 2.47 | 0.5741 | 2.37 |
| Mali | 2.45 | 0.2686 | 2.35 |
| Palau | 2.42 | 0.7540 | 2.32 |
| Niue | 2.42 | 0.7262 | 2.32 |
| Jordan | 2.37 | 0.7253 | 2.27 |
| South Sudan | 2.37 | 0.2784 | 2.26 |
| Turkey | 2.36 | 0.7127 | 2.26 |
| Tonga | 2.21 | 0.6263 | 2.11 |
| Botswana | 2.09 | 0.6427 | 1.99 |
| Cuba | 2.09 | 0.6687 | 1.99 |
| Djibouti | 2.06 | 0.4880 | 1.97 |
| Panama | 2.06 | 0.7089 | 1.96 |
| United Republic of Tanzania | 2.00 | 0.4466 | 1.90 |
| Peru | 1.99 | 0.6621 | 1.89 |
| Namibia | 1.95 | 0.6176 | 1.85 |
| Togo | 1.91 | 0.4085 | 1.81 |
| Tajikistan | 1.85 | 0.5415 | 1.75 |
| Spain | 1.84 | 0.7693 | 1.75 |
| Bolivia (Plurinational State of) | 1.81 | 0.5990 | 1.71 |
| Morocco | 1.74 | 0.5627 | 1.65 |
| Gambia | 1.73 | 0.4097 | 1.63 |
| Malawi | 1.72 | 0.3846 | 1.63 |
| Costa Rica | 1.69 | 0.7003 | 1.59 |
| Italy | 1.68 | 0.8058 | 1.58 |
| Kenya | 1.68 | 0.5238 | 1.58 |
| Mongolia | 1.63 | 0.6176 | 1.53 |
| Maldives | 1.59 | 0.6509 | 1.50 |
| Kazakhstan | 1.56 | 0.7251 | 1.46 |
| Albania | 1.55 | 0.7068 | 1.45 |
| Suriname | 1.55 | 0.6337 | 1.45 |
| Vanuatu | 1.53 | 0.4731 | 1.44 |
| Dominican Republic | 1.53 | 0.6194 | 1.43 |
| Lao People's Democratic Republic | 1.50 | 0.4891 | 1.41 |
| Nigeria | 1.49 | 0.5034 | 1.40 |
| Nicaragua | 1.49 | 0.5240 | 1.39 |
| South Africa | 1.48 | 0.6796 | 1.38 |
| Sri Lanka | 1.48 | 0.7015 | 1.38 |
| Guinea | 1.47 | 0.3364 | 1.37 |
| Ethiopia | 1.46 | 0.3588 | 1.36 |
| Denmark | 1.38 | 0.8964 | 1.29 |
| Uzbekistan | 1.34 | 0.6626 | 1.24 |
| Greece | 1.30 | 0.7919 | 1.20 |
| Zambia | 1.30 | 0.5059 | 1.20 |
| Mozambique | 1.30 | 0.3265 | 1.20 |
| Cote d'Ivoire | 1.29 | 0.4259 | 1.19 |
| Belarus | 1.28 | 0.7845 | 1.18 |
| Comoros | 1.23 | 0.4760 | 1.14 |
| Hungary | 1.22 | 0.7908 | 1.12 |
| Mexico | 1.22 | 0.6646 | 1.12 |
| Montenegro | 1.19 | 0.7958 | 1.09 |
| Equatorial Guinea | 1.18 | 0.6579 | 1.09 |
| Ecuador | 1.15 | 0.6610 | 1.05 |
| Bosnia and Herzegovina | 1.14 | 0.7231 | 1.04 |
| Chile | 1.14 | 0.7715 | 1.04 |
| Rwanda | 1.13 | 0.4356 | 1.04 |
| North Macedonia | 1.12 | 0.7506 | 1.02 |
| Cyprus | 1.10 | 0.8356 | 1.00 |
| Greenland | 1.10 | 0.8262 | 1.00 |
| Eswatini | 1.09 | 0.5855 | 0.99 |
| Austria | 1.09 | 0.8538 | 0.99 |
| Chad | 1.25 | 0.2404 | 0.98 |
| Belgium | 1.05 | 0.8537 | 0.95 |
| El Salvador | 1.04 | 0.5638 | 0.95 |
| Argentina | 1.04 | 0.7231 | 0.95 |
| Ukraine | 1.04 | 0.7608 | 0.94 |
| Guatemala | 1.03 | 0.5400 | 0.93 |
| Honduras | 1.02 | 0.5130 | 0.93 |
| Croatia | 1.00 | 0.7983 | 0.90 |
| Iceland | 0.99 | 0.8764 | 0.90 |
| Bhutan | 0.99 | 0.4731 | 0.89 |
| Angola | 0.98 | 0.4537 | 0.88 |
| Venezuela (Bolivarian Republic of) | 0.96 | 0.5965 | 0.86 |
| Benin | 0.96 | 0.3735 | 0.86 |
| Monaco | 0.95 | 0.9083 | 0.85 |
| Russian Federation | 0.94 | 0.8085 | 0.84 |
| San Marino | 0.92 | 0.8880 | 0.82 |
| Mauritania | 0.91 | 0.4989 | 0.82 |
| Andorra | 0.91 | 0.8694 | 0.81 |
| Luxembourg | 0.90 | 0.8844 | 0.81 |
| Latvia | 0.90 | 0.8307 | 0.80 |
| Colombia | 0.90 | 0.6554 | 0.80 |
| Lithuania | 0.89 | 0.8565 | 0.80 |
| Czechia | 0.89 | 0.8285 | 0.79 |
| Ghana | 0.89 | 0.5649 | 0.79 |
| Switzerland | 0.87 | 0.9331 | 0.77 |
| Serbia | 0.87 | 0.7924 | 0.77 |
| Jamaica | 0.87 | 0.6833 | 0.77 |
| United Kingdom | 0.85 | 0.8590 | 0.76 |
| France | 0.85 | 0.8384 | 0.75 |
| Slovenia | 0.84 | 0.8424 | 0.74 |
| Ireland | 0.84 | 0.8738 | 0.74 |
| Bermuda | 0.83 | 0.8214 | 0.73 |
| Malta | 0.83 | 0.8016 | 0.73 |
| Germany | 0.83 | 0.9030 | 0.73 |
| Belize | 0.83 | 0.6102 | 0.73 |
| United States of America | 0.81 | 0.8624 | 0.71 |
| Poland | 0.79 | 0.8120 | 0.69 |
| Estonia | 0.77 | 0.8449 | 0.67 |
| Puerto Rico | 0.77 | 0.8255 | 0.67 |
| Canada | 0.76 | 0.8732 | 0.66 |
| New Zealand | 0.75 | 0.8494 | 0.65 |
| Israel | 0.75 | 0.8090 | 0.65 |
| Sao Tome and Principe | 0.74 | 0.5054 | 0.64 |
| Sweden | 0.74 | 0.8869 | 0.64 |
| Netherlands | 0.72 | 0.8885 | 0.62 |
| Republic of Korea | 0.72 | 0.8867 | 0.62 |
| Liberia | 0.72 | 0.3524 | 0.62 |
| Burkina Faso | 0.72 | 0.2851 | 0.62 |
| United States Virgin Islands | 0.71 | 0.8218 | 0.61 |
| Japan | 0.69 | 0.8712 | 0.59 |
| Senegal | 0.69 | 0.4081 | 0.59 |
| Congo | 0.68 | 0.5831 | 0.59 |
| Slovakia | 0.68 | 0.8106 | 0.58 |
| Bahamas | 0.67 | 0.8050 | 0.57 |
| Guyana | 0.66 | 0.6508 | 0.56 |
| Antigua and Barbuda | 0.65 | 0.7499 | 0.55 |
| Saint Vincent and the Grenadines | 0.65 | 0.6372 | 0.55 |
| Cabo Verde | 0.64 | 0.5335 | 0.55 |
| Brunei Darussalam | 0.64 | 0.8102 | 0.54 |
| Trinidad and Tobago | 0.64 | 0.7688 | 0.54 |
| Eritrea | 0.63 | 0.4039 | 0.53 |
| Norway | 0.63 | 0.9161 | 0.53 |
| Romania | 0.60 | 0.7685 | 0.50 |
| Australia | 0.60 | 0.8443 | 0.50 |
| Finland | 0.60 | 0.8598 | 0.50 |
| Saint Lucia | 0.58 | 0.6725 | 0.48 |
| Cameroon | 0.58 | 0.4797 | 0.48 |
| Haiti | 0.57 | 0.4483 | 0.47 |
| Uganda | 0.55 | 0.4233 | 0.45 |
| Guinea-Bissau | 0.55 | 0.3531 | 0.45 |
| Dominica | 0.53 | 0.7470 | 0.43 |
| Portugal | 0.53 | 0.7442 | 0.43 |
| Sierra Leone | 0.51 | 0.3587 | 0.41 |
| Bulgaria | 0.50 | 0.7682 | 0.40 |
| Uruguay | 0.50 | 0.7193 | 0.40 |
| Grenada | 0.49 | 0.6690 | 0.39 |
| Singapore | 0.45 | 0.8561 | 0.35 |
| Niger | 0.87 | 0.1681 | 0.34 |
| Democratic People's Republic of Korea | 0.43 | 0.5699 | 0.33 |
| Saint Kitts and Nevis | 0.42 | 0.7550 | 0.33 |
| Gabon | 0.41 | 0.6347 | 0.31 |
| Madagascar | 0.36 | 0.4002 | 0.26 |
| Central African Republic | 0.32 | 0.3092 | 0.21 |
| Burundi | 0.28 | 0.2894 | 0.18 |
| Taiwan (Province of China) | 0.24 | 0.8747 | 0.14 |
| Barbados | 0.22 | 0.7467 | 0.12 |
| Democratic Republic of the Congo | 0.10 | 0.3832 | 0.00 |
| Somalia | 1.07 | 0.0777 | 0.00 |

**Table S7.** The future ASYR of cataract attributable to HAP and smoking globally from 2022 to 2050 by ARIMA model.

| **Sex** | **2022** | **2050** |
| --- | --- | --- |
|  | **ASYR**  **per 100 000**  **(95% UI)** | **ASYR**  **per 100 000**  **(95% UI)** |
| **Global (HAP)** |  |  |
| **Both** | 22.71 (22.51–22.91) | 11.18 (–28.34–50.70) |
| **Males** | 20.82 (20.64–20.92) | 14.03 (–20.01–48.07) |
| **Females** | 24.45 (24.22–24.68) | 8.31 (–36.74–53.37) |
| **Global (Smoking)** |  |  |
| **Both** | 2.53 (2.49–2.57) | 1.08 (0.62–1.55) |
| **Males** | 4.42 (4.35–4.50) | 1.81 (1.18–2.44) |
| **Females** | 0.84 (0.82–0.86) | –0.08 (–1.73–1.58) |





**Figure S1:** ACF and PACF plots (A, B) and residual diagnostic panels (C, D) for global ASYR time series of cataract attributable to HAP for males (A, C) and females (B, D), 1990–2021. All residual lags fall within the 95% confidence bounds (blue dashed lines), confirming white noise distribution and adequate model fitting. Residual diagnostics in panels C and D consist of residual time-series plot, residual ACF, residual PACF, and normal Q–Q plot sequentially. ASYR: age-standardised YLD rate. HAP: household air pollution. ACF: autocorrelation function. PACF: partial autocorrelation function.





**Figure S2:** ACF and PACF plots (A, B) and residual diagnostic panels (C, D) for global ASYR time series of cataract attributable to smoking for males (A, C) and females (B, D), 1990–2021. All residual lags fall within the 95% confidence bounds (blue dashed lines), confirming white noise distribution and adequate model fitting. Residual diagnostics in panels C and D consist of residual time-series plot, residual ACF, residual PACF, and normal Q–Q plot sequentially. ASYR: age-standardised YLD rate. ACF: autocorrelation function. PACF: partial autocorrelation function.


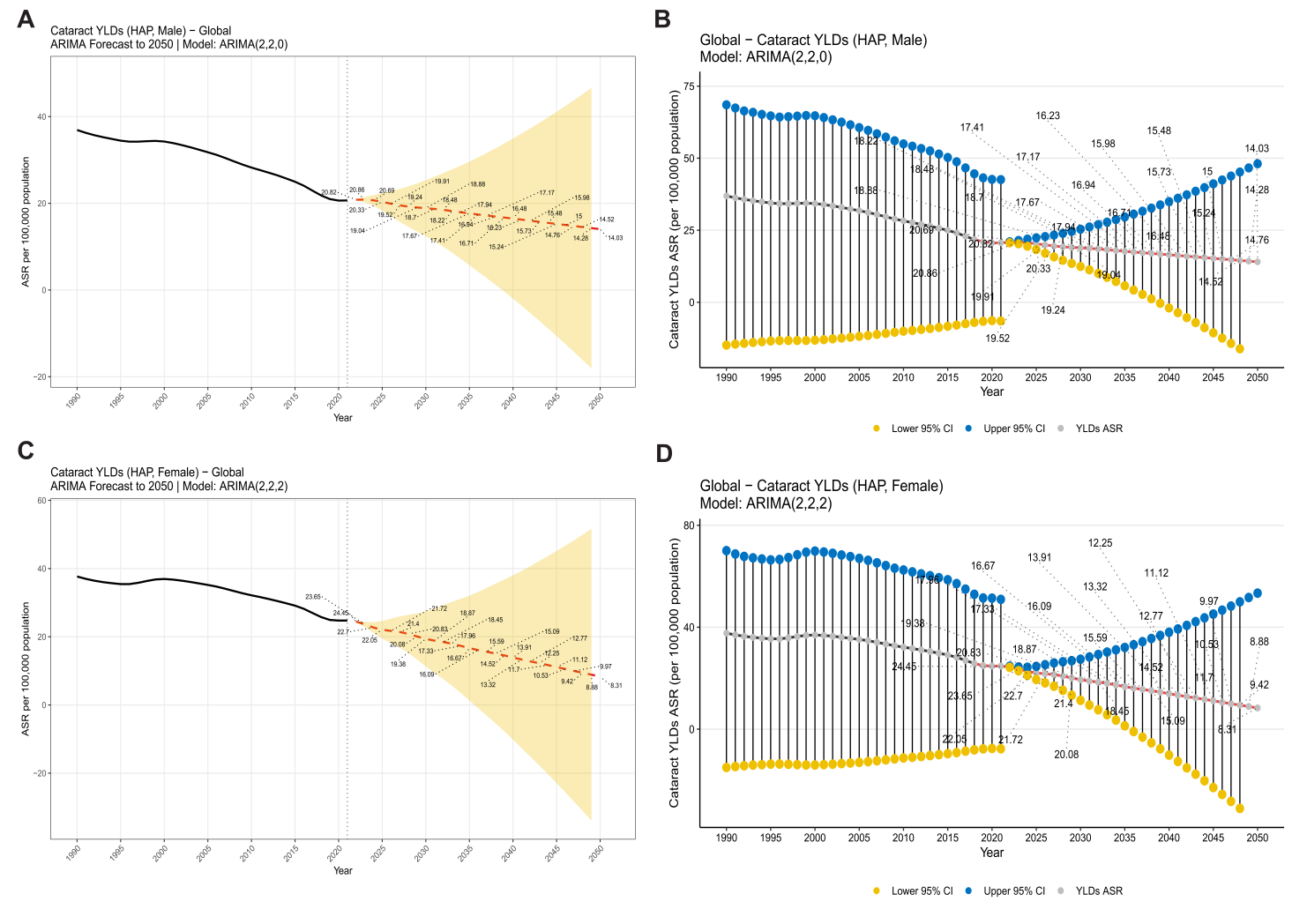


**Figure S3:** ARIMA models were applied to project trends in cataract ASYR attributable to HAP for males (A, B) and females (C, D) from 1990 to 2050. Panels A and C show overall temporal trends, while Panels B and D present detailed uncertainty information. ASYR: age-standardised YLD rate. HAP: household air pollution. ARIMA: autoregressive integrated moving average.





**Figure S4:** ARIMA models were applied to project trends in cataract ASYR attributable to smoking for males (A, B) and females (C, D) from 1990 to 2050. Panels A and C show overall temporal trends, while Panels B and D present detailed uncertainty information. ASYR: age-standardised YLD rate. ARIMA: autoregressive integrated moving average.
